# Gene-Environment Interactions in Stress Response Contribute Additively to a Genotype-Environment Interaction

**DOI:** 10.1371/journal.pgen.1006158

**Published:** 2016-07-20

**Authors:** Takeshi Matsui, Ian M. Ehrenreich

**Affiliations:** Molecular and Computational Biology Section, Department of Biological Sciences, University of Southern California, Los Angeles, California, United States of America; Georgia Institute of Technology, UNITED STATES

## Abstract

How combinations of gene-environment interactions collectively give rise to genotype-environment interactions is not fully understood. To shed light on this problem, we genetically dissected an environment-specific poor growth phenotype in a cross of two budding yeast strains. This phenotype is detectable when certain segregants are grown on ethanol at 37°C (‘E37’), a condition that differs from the standard culturing environment in both its carbon source (ethanol as opposed to glucose) and temperature (37°C as opposed to 30°C). Using recurrent backcrossing with phenotypic selection, we identified 16 contributing loci. To examine how these loci interact with each other and the environment, we focused on a subset of four loci that together can lead to poor growth in E37. We measured the growth of all 16 haploid combinations of alleles at these loci in all four possible combinations of carbon source (ethanol or glucose) and temperature (30 or 37°C) in a nearly isogenic population. This revealed that the four loci act in an almost entirely additive manner in E37. However, we also found that these loci have weaker effects when only carbon source or temperature is altered, suggesting that their effect magnitudes depend on the severity of environmental perturbation. Consistent with such a possibility, cloning of three causal genes identified factors that have unrelated functions in stress response. Thus, our results indicate that polymorphisms in stress response can show effects that are intensified by environmental stress, thereby resulting in major genotype-environment interactions when multiple of these variants co-occur.

## Author Summary

Determining the genetic and molecular mechanisms that give rise to genotype-environment interaction (‘GxE’) is important for many areas of biology, including agriculture, evolution, and medicine. To help advance knowledge regarding this topic, we dissect the genetic basis of an example of GxE in which certain *Saccharomyces cerevisiae* cross progeny show extremely poor growth specifically on ethanol at 37°C. This environment differs from the standard condition used for culturing budding yeast in both its carbon source (ethanol as opposed to glucose) and temperature (37°C as opposed to 30°C). We provide evidence that poor growth on ethanol at 37°C is caused by a number of predominantly additive loci that individually exhibit gene-environment interactions with both carbon source and temperature. These loci show their largest effects when carbon source and temperature are simultaneously modified, indicating their effect magnitudes may be influenced by the severity of environmental stress. Consistent with this possibility, we clone three causal genes and find they encode functionally unrelated components of stress response. Our work suggests that polymorphisms in stress response can contribute additively to genotype-environment interactions that vary in intensity across conditions in a stress level-dependent manner.

## Introduction

Genotype-environment interaction (‘GxE’) occurs when genetically distinct individuals show different phenotypic responses to the environment [1,2]. Although GxE is known to influence many agriculturally, evolutionarily, and medically relevant traits (e.g., [3–6]), our basic knowledge of the genetic and molecular mechanisms that underlie GxE remains incomplete. Recent work on this topic in *Saccharomyces cerevisiae* suggests GxE can arise due to not only individual loci that show gene-environment interactions, but also sets of loci that show environment-dependent epistatic interactions [7–9]. However, because the underlying genetic basis of GxE has only been comprehensively dissected in a small number of cases (e.g., [7–9]), the relative contributions of these different types of genetic effects to GxE is unclear.

**Fig 1:**
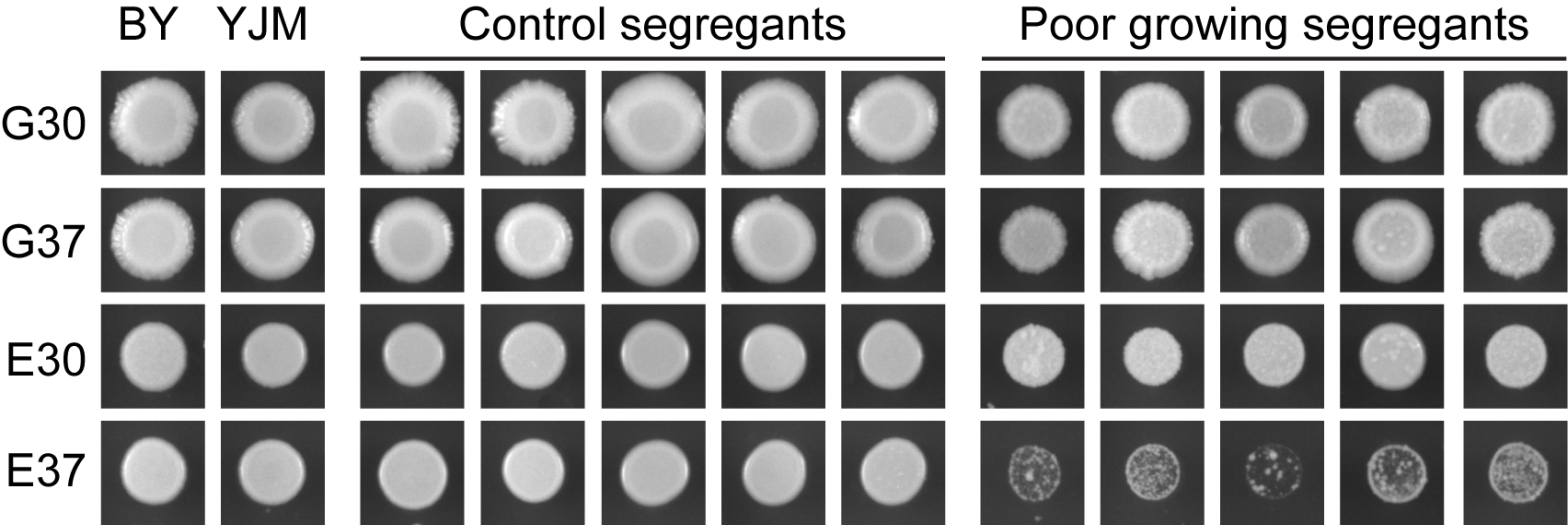
Representative images of BY, YJM, control segregants, and poor growing segregants under four conditions: glucose at 30°C, glucose at 37°C, ethanol at 30°C, and ethanol at 37°C. We refer to these conditions throughout the paper as ‘G30’, ‘G37’, ‘E30’, and ‘E37’, respectively.

Here, we generate an additional, detailed example of the genetic basis of GxE in the budding yeast *Saccharomyces cerevisiae*. We focus on characterizing the genetic basis of a poor growth phenotype that occurs specifically when certain segregants from a cross of the BY4716 (‘BY’) lab strain and the YJM789 (‘YJM’) clinical isolate [10] are cultured on ethanol at 37°C (‘E37’; **Fig 1**). Although yeast is typically grown on glucose as the carbon source and at 30°C as the temperature (‘G30’), it can tolerate a broad range of environmental conditions, including other carbon sources and temperatures [10,11]. Among the different carbon sources that yeast can utilize, ethanol can be particularly stressful because it is metabolized via respiration instead of fermentation, which results in increased oxidative stress [12]. Furthermore, high temperature is known to be a stressor for budding yeast [13], with some isolates incapable of growing at 37°C or above [11,14–20].

**Fig 2:**
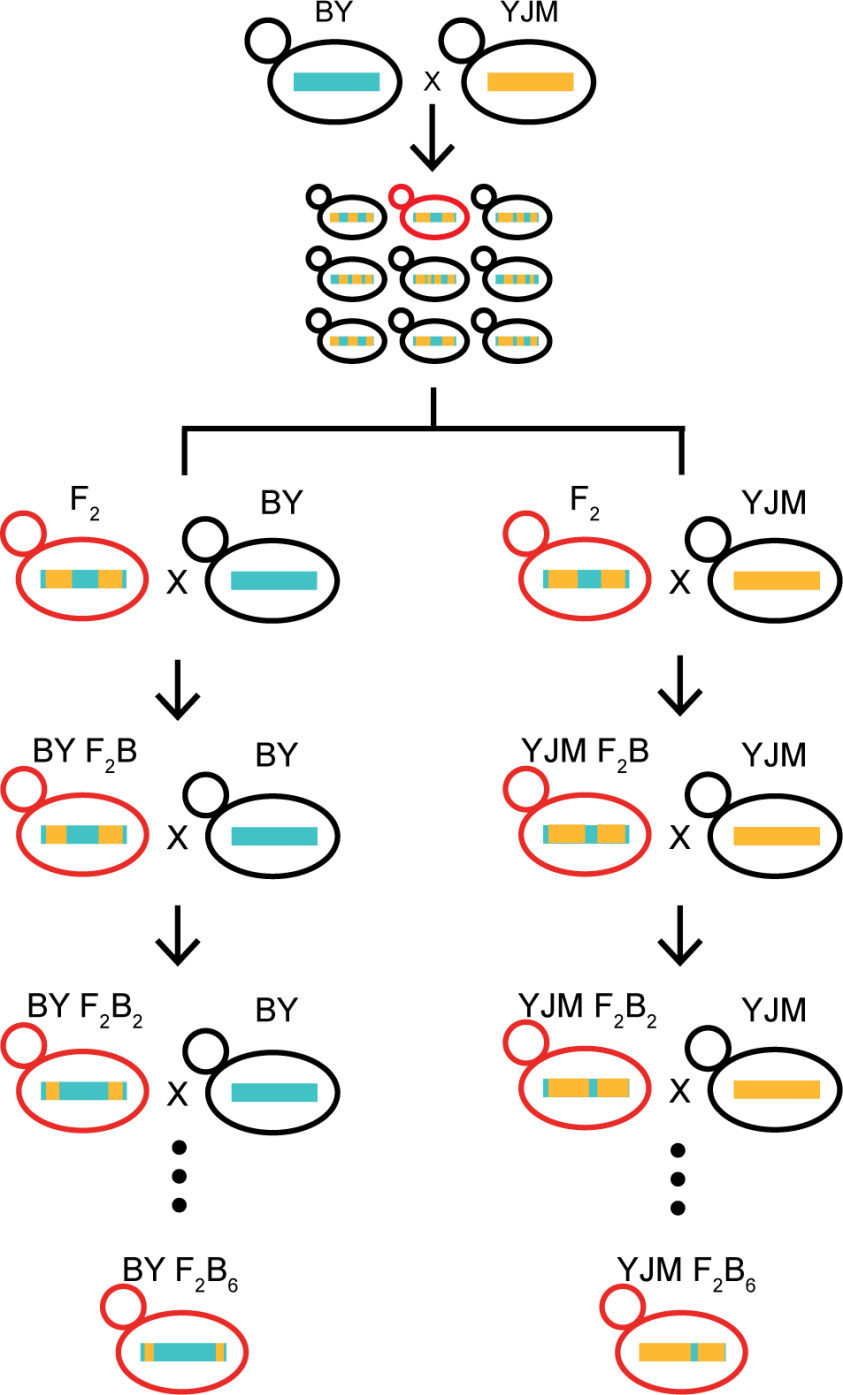
The crossing scheme used to generate BY and YJM F_2_B_6_ NILs. First, haploid versions of BY and YJM were mated, and the resulting F_1_ diploid was sporulated to generate haploid F_2_ segregants. These F_2_s were then screened for growth in E37. A single F_2_ exhibiting poor growth in E37 (shown in red) was chosen to serve as the progenitor for backcrossing. This F_2_ was then backcrossed to both BY and YJM, and the resulting diploids were sporulated to generate haploid F_2_B backcross segregants. Seven BY and seven YJM F_2_Bs that grow poorly in E37 were selected to serve as the progenitors for additional backcrossing. Next, these strains were subjected to five additional rounds of mating to the appropriate parent, sporulation, and selection for the poor growth phenotype to create 14 independent backcross lineages. Finally, a single haploid F_2_B_6_ exhibiting poor growth in E37 was chosen from each backcross lineage and designated as a Nearly Isogenic Line (NIL). These NILs are expected to carry combinations of alleles from one parent that collectively lead to poor growth in E37 when they co-occur in the genetic background of the other parent.

To determine the genetic basis of poor growth in E37, we use a genetic mapping strategy involving recurrent backcrossing with phenotypic selection (**Fig 2**). Through this approach, we identify 16 loci that contribute to poor growth in E37. We then conduct a more detailed study of four of these loci, which collectively result in poor growth in E37 when they co-occur in the YJM background. By analyzing the growth of all 16 haploid multi-locus genotypes involving the loci on all four combinations of two carbon sources (glucose and ethanol) and two temperatures (30 and 37°C), we find that the four loci contribute to poor growth in E37 in a primarily additive manner. Furthermore, we also show that these loci exhibit weaker, negative effects on growth when only carbon source or temperature is altered relative to standard conditions. These results indicate that GxE in our system reflects the composite effect of multiple additive loci that show condition-dependent effect magnitudes. Additionally, by resolving three of these loci to a component of the vacuolar protein sorting machinery (*VPS70*), a stress granule-associated RNA binding protein (*YGR250C*), and a stress responsive kinase (*IKS1*), we implicate genetic variation in stress response as the source of the identified gene- and genotype-environment interactions.

## Results and Discussion

### Genetic mapping of poor growth in E37 by recurrent backcrossing and selection

**Fig 3:**
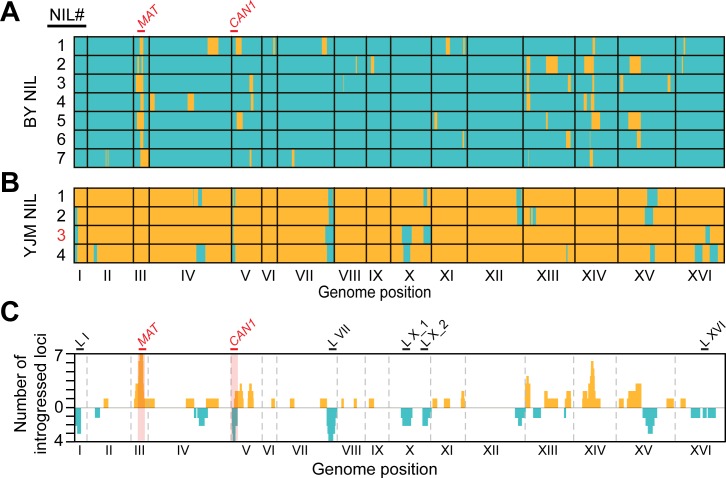
Introgressed genomic regions detected in the NILs. (**A**) Loci from YJM that were introgressed into the BY genetic background are shown as orange boxes against a blue BY genetic background. (**B**) Loci from BY that were introgressed into the YJM genetic background are shown as blue boxes against an orange YJM genetic background. YJM NIL 3, which served as the progenitor of the F_2_B_7_ population described later, is highlighted in red (**C**) The number of times each region was introgressed is shown. Selection markers used to generate haploid progeny—*MAT* and *CAN*—are also highlighted in red. The Chromosome I, VII, X_1, X_2, and XVI loci that segregate in the F_2_B_7_ population are denoted as ‘L I’, ‘L VII’,’L X_1’,’L X_2’,’ and ‘L XVI’, respectively.

We screened 112 haploid BYxYJM F_2_s for growth on both glucose and ethanol at both 30 and 37°C. We found that five of these individuals exhibited noticeably poor growth specifically in E37 (**Fig 1**). To determine the genetic basis of this phenotype, we used a recurrent backcrossing with phenotypic selection strategy (**Fig 2**). In brief, we mated one of the five poor growing F_2_s to both BY and YJM, and generated and phenotyped at least 576 haploid F_2_B recombinants from each backcross (**Methods**). 14 F_2_Bs (seven per backcross) were then used to breed haploid Nearly Isogenic Lines (NILs) that carry alleles that collectively cause poor growth in E37 (**Fig 2**; **Methods**). To identify these alleles, we sequenced the genomes of the NILs to an average per site coverage of 21X and identified genomic regions that had been introgressed (**Fig 3**; **Methods**). Based on these data, we determined that three of the YJM NILs harbored aneuploidies or appeared to be replicates of other NILs (**S1** and **S2 Figs**). We excluded these individuals from all subsequent analyses. Among the remaining 11 NILs, we detected 41 introgressed genomic regions (**Fig 3**).

### Many introgressed loci have biological effects

To verify that the introgressed regions contribute to poor growth in E37, we generated a population of haploid F_2_B_7_s by backcrossing YJM NIL 3 to YJM an additional time. Ignoring a control marker at *CAN1* on Chromosome V, five genomic regions (Chromosome I, VII, X_1, X_2, and XVI), were polymorphic in the F_2_B_7_ population (**Fig 3B** and **3C**). Four of these loci were detected in other YJM NILs (Chromosome I, VII, X_1, and X_2), while the genomic region on Chromosome XVI was unique to this NIL (**Fig 3C**). By screening 864 F_2_B_7_s, we obtained 45 individuals that grow poorly in E37 (**Methods**). These individuals, as well as a distinct population of 192 random F_2_B_7_s, were then genotyped by low coverage whole genome sequencing or restriction enzyme typing (**Methods**). We tested for allelic enrichment among the poor growing individuals relative to the random controls (**Methods**). Fisher’s exact tests indicate that the Chromosome I, VII, X_1, and X_2 loci contribute to YJM NIL 3’s poor growth in E37 (I: p ≤ 3.8 x 10^−8^, VII: p ≤ 4 x 10^−20^, X_1: p ≤ 8.4 x 10^−7^, X_2: p ≤ 1.6 x 10^−20^; **S3 Fig**), while the Chromosome XVI locus does not (XVI: p = 0.34; **S3 Fig**). Given that the former loci were detected in two or more NILs and the latter locus was only identified in a single NIL, these results suggest that loci that were detected independently at least twice among the NILs have biological effects. Extension of this finding to the entire set of introgressed genomic regions conservatively implicates at least 16 loci as contributors to poor growth in E37 (**Fig 3C**; **S1 Table**).

### Loci involved in poor growth in E37 mainly act in an additive manner

**Fig 4:**
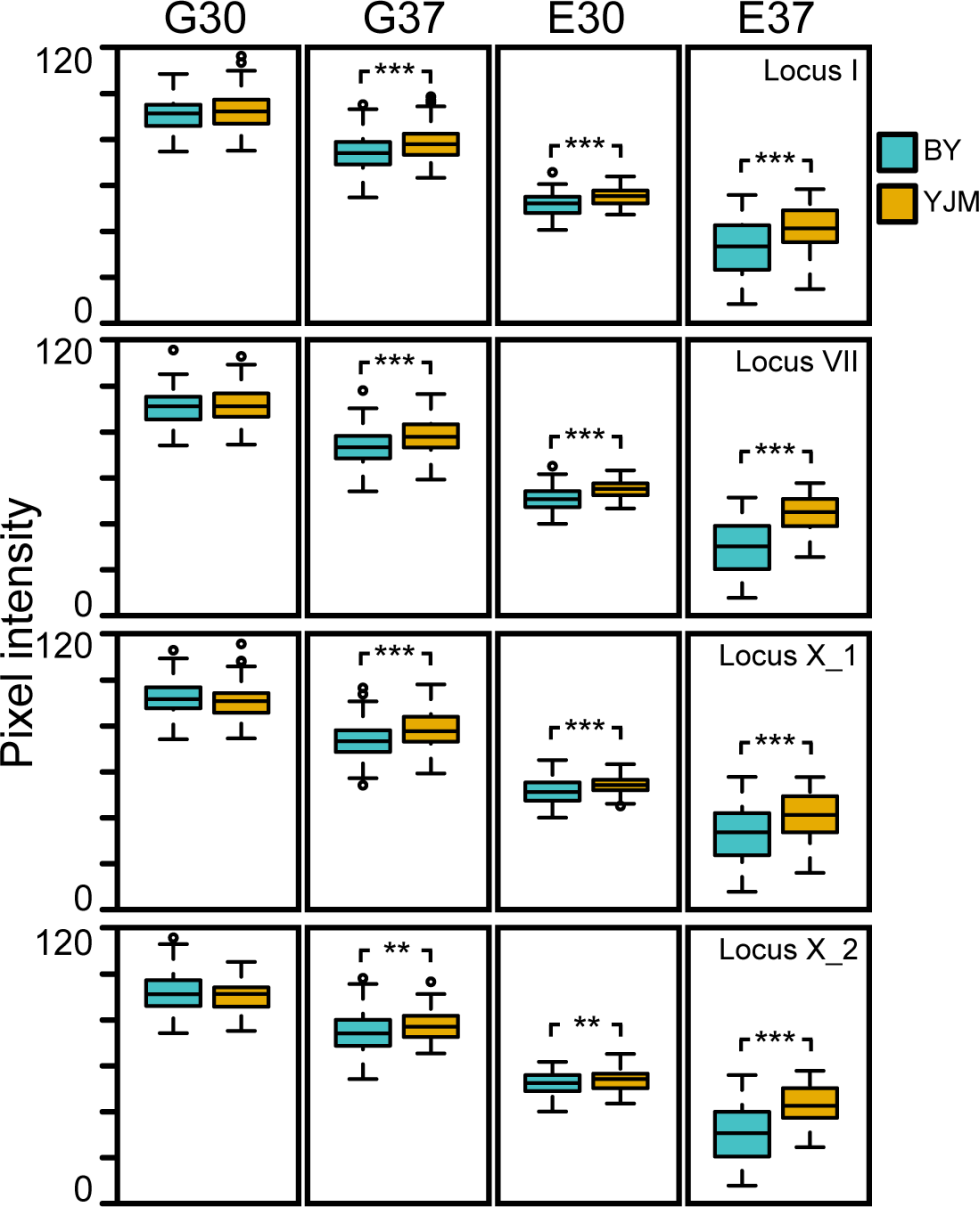
Phenotypic effects of the Chromosome I, VII, X_1, X_2, and XVI loci in the four conditions. Box plots showing the phenotypic effects of the four loci among the F_2_B_7_s in each condition. Individuals carrying the BY and YJM alleles at each locus are shown in blue and orange, respectively. Statistical significance was assessed using factor effect tests obtained from the full factorial ANOVAs described in the main text. All four loci were found to have statistically significant phenotypic effect on growth in G37, E30, and E37. ** and *** denote *p* ≤ 0.01 and *p* ≤ 0.001, respectively.

We analyzed the phenotypic effects of the Chromosome I, VII, X_1, and X_2 loci using the population of 192 random F_2_B_7_s (**Methods**). These strains were quantitatively phenotyped for growth in E37, and the additive and epistatic effects of the four loci were assessed (**Methods**). In a full factorial ANOVA that included all possible additive effects and pairwise or higher-order epistatic interactions (**Methods**), genetic factors explained 79.9% of the phenotypic variance (**Table 1**). 94 and 6% of this genetic contribution to growth was due to additive and epistatic effects, respectively. Furthermore, 7, 11.1, 24.7, and 32.4% of the phenotypic variance was explained by the Chromosome X_1, I, X_2, and VII loci, respectively (**Table 1**). Each of these additive effects were highly significant (F statistic > 60, d.f._numerator_, = 1, d.f._residuals_ = 175, p < 6 x 10^−13^; **Fig 4**; **Table 1**). In contrast, only four epistatic interactions showed significant effects (F statistic > 5.2, d.f._numerator_, = 1, d.f._residuals_ = 175, p < 0.024). These were each pairwise interactions that explained only between 0.6 and 2% of the phenotypic variance (**Table 1**). Thus, our results indicate that extremely poor growth in E37 has a genetic basis that is mostly additive.

**Table 1:** Full factorial ANOVA for E37 condition.

Source	Df	Sum Sq	Mean Sq	F value	Pr(>F)	PVE
I	1	3360.3	3360.3	96.142	<2.2e-16	11.1
VII	1	9836.2	9836.2	281.427	<2.2e-16	32.4
X_1	1	2116.2	2116.2	60.546	5.986e-13	7.0
X_2	1	7492.9	7492.9	214.383	<2.2e-16	24.7
I:VII	1	90.2	90.2	2.579	0.110031	0.3
I:X_1	1	19.1	19.1	0.545	0.460992	0.1
VII:X_1	1	597.4	597.4	17.092	5.516e-05	2.0
I:X_2	1	181.2	181.2	5.185	0.023983	0.6
VII:X_2	1	308	308	8.811	0.003413	1.0
X_1:X_2	1	221.1	221.1	6.326	0.01279	0.7
I:VII:X_1	1	3.1	3.1	0.087	0.767754	0.0
I:VII:X_2	1	28.5	28.5	0.815	0.367612	0.1
I:X_1:X_2	1	8.6	8.6	0.244	0.621402	0
VII:X_1:X_2	1	0.5	0.5	0.013	0.908321	0
I:VII:X_1:X_2	1	0.5	0.5	0.012	0.909587	0
Residuals	175	6116.4	35			

PVE, percent of phenotypic variance explained. Interaction terms are denoted by ‘:’.

### Loci show gene-environment interactions with both carbon source and temperature

We also examined the effects of the Chromosome I, VII, X_1, and X_2 loci in G30, ethanol at 30°C (‘E30’), and glucose at 37°C (‘G37’). As a first step, full factorial ANOVA models were implemented in each of these conditions. In G30, the only nominally significant effect was a higher-order epistatic interaction involving all four loci, which explained 3.3% of the phenotypic variance (F statistic = 6.4, d.f._numerator_, = 1, d.f._residuals_ = 175, p < 0.013; **S2 Table**). In comparison, full factorial models for E30 and G37 revealed that all four loci showed significant additive effects in both conditions (F statistic > 7.3, d.f._numerator_, = 1, d.f._residuals_ = 175, p < 0.004; **Fig 4**; **S3** and **S4 Tables**). The only other significant genetic effect in E30 or G37 occurred in the former condition, with a pairwise epistatic interaction detected between the Chromosome VII and X_1 loci (F statistic = 15.5, d.f._numerator_, = 1, d.f._residuals_ = 175, p = 0.0001; **S3 Table**). These results show that the Chromosome I, VII, X_1, and X_2 loci are influenced by both carbon source and temperature, and act in a largely additive manner within a given non-standard growth condition (**Fig 4**; **Table 1**; **S2** through **S4 Tables**).

### Loci show a negative relationship between average growth level and additive effect size

**Fig 5:**
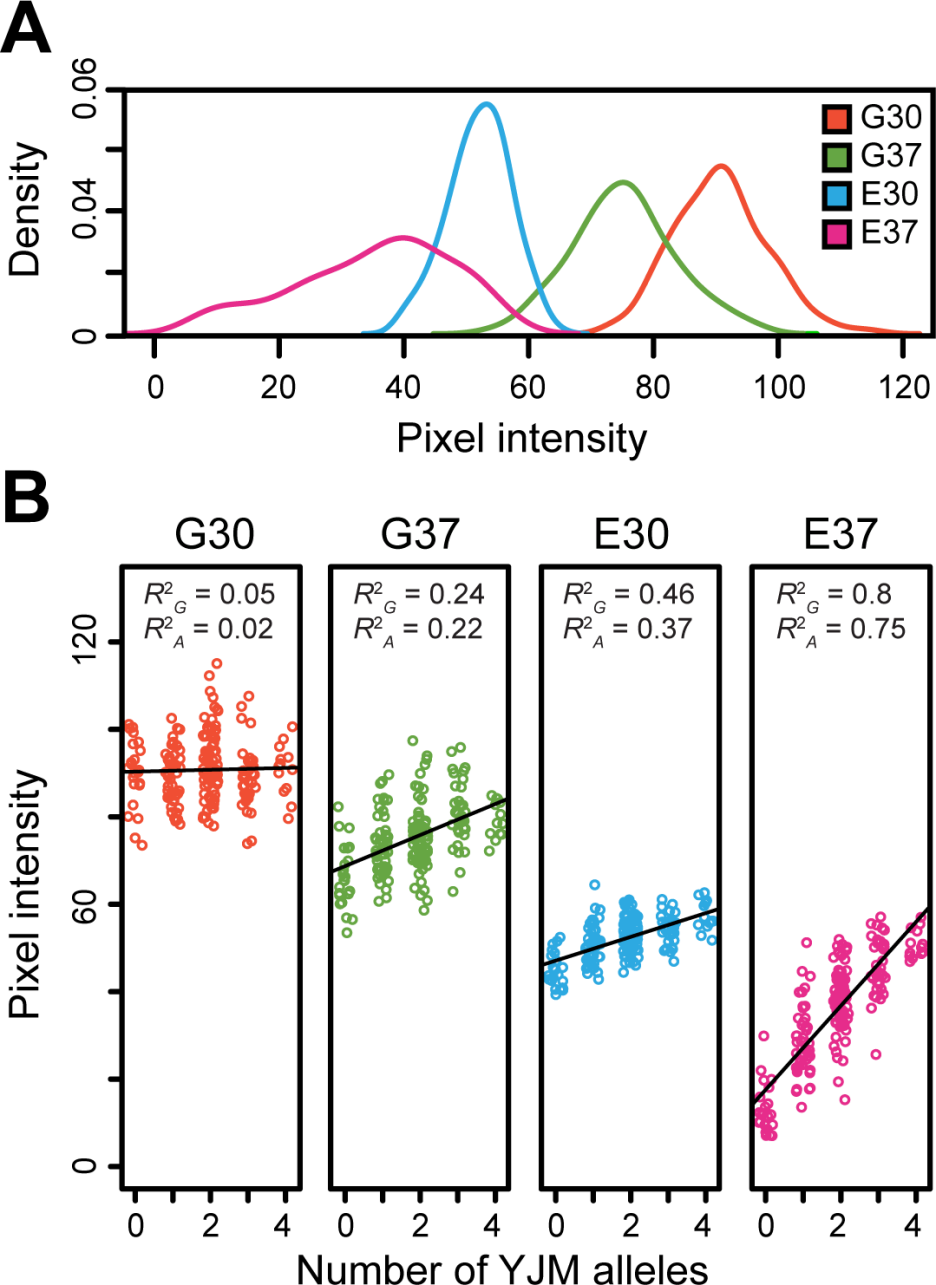
Analysis of growth among 192 random F_2_B_7_s across the four conditions. (**A**) Density plots of the median pixel intensities observed among segregants are plotted for each condition. (**B**) Within each condition, the relationship between number of YJM alleles carried by a segregant across the Chromosome I, VII, X_1, X_2, and XVI loci and phenotype is plotted. The black lines represent equal size, additive effect regression models that were fit to the data for each condition (**Methods**). Despite variability in effect sizes among the four loci, these models were highly significant in G37, E30, and E37 (*p* ≤ 10^−10^), but not G30 (*p* = 0.67). Also, for each condition, the fraction of phenotypic variance explained by all genetic factors and only additive genetic factors are noted by *R*^2^_G_ and *R*^2^_A_, respectively. These values were obtained from the full factorial ANOVA models for each condition, rather than from the simpler regression models illustrated in the plots (**Methods**).

We next assessed the relationship between the effects of the Chromosome I, VII, X_1, and X_2 loci and the different conditions. Based on the aforementioned full factorial models, we found that the average percent phenotypic variance explained by the additive effects of the four loci was 0.48, 5.4, 9.2, and 18.8% in G30, G37, E30, and E37, respectively. These changes in average effect size across conditions show a negative association with the average growth levels seen among F_2_B_7_s in the respective conditions, which exhibit the relationship G30 > G37 > E30 > E37 (**Fig 5A**). These reductions in average growth levels across conditions may reflect increases in environmental stress, suggesting that lower absolute growth, higher stress, or a combination of the two intensifies the effect magnitudes of the loci (**Fig 4**). This finding helps explain how gene- and genotype-environment interactions of varying magnitudes can occur across conditions, while variability in growth can remain predominantly additive in its genetic basis within a condition (**Fig 5B**).

### Causal genes play roles in stress response

**Fig 6:**
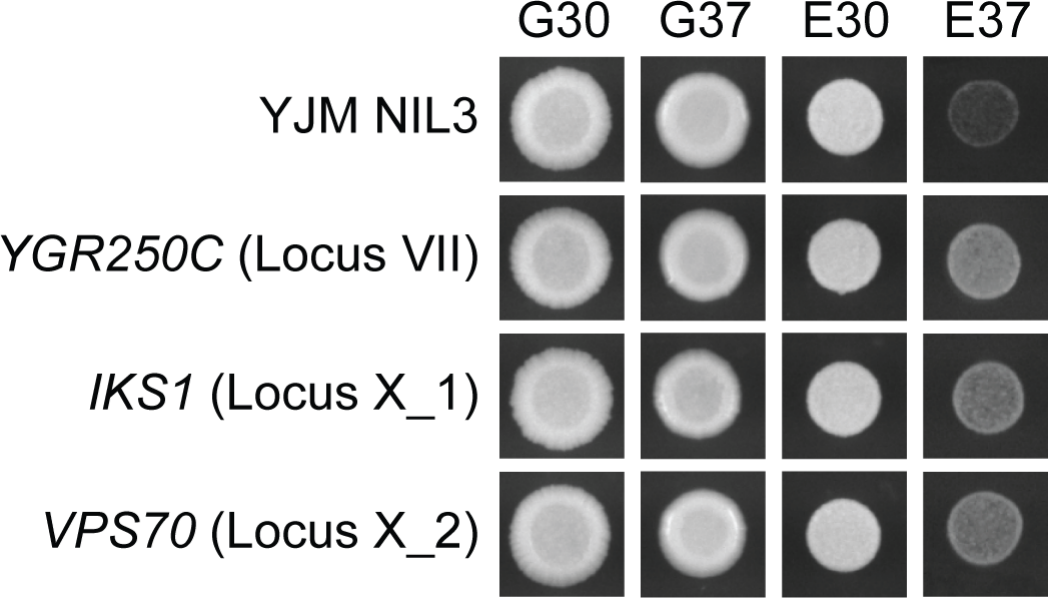
Identification of the causal genes underlying the Chromosome VII, X_1, and X_2 loci. Comparison of YJM NIL 3 to the *YGR250C*^YJM^, *IKS1*^YJM^, and *VPS70*^YJM^ allele replacement strains supports a causal role for these genes in poor growth in E37.

To help determine the mechanism that relates average growth level within a condition to the effect sizes of the four loci, we attempted to clone the causal genes underlying the loci. The F_2_B_7_ data allowed us to resolve the Chromosome I, VII, X_1, and X_2 loci to small intervals containing on average 5,943 bp (**S5 Table**; **S1 Note**; **Methods**). For each candidate gene in each locus, we performed allele replacements that included the promoter and coding region (**Methods**). Specifically, the existing BY allele of each candidate gene was replaced with the YJM allele in YJM NIL 3 (**Methods**). Through these experiments, we were able to resolve the Chromosome VII, X-1, and X-2 loci to *YGR250C*, *IKS1*, and *VPS70*, respectively (**Fig 6**). *YGR250C* encodes a RNA binding protein that localizes to stress granules [21–23]. Stress granules are cytoplasmic messenger ribonucleoprotein (mRNPs) complexes that form in response to stress and are thought to aid in the translation of mRNAs by increasing the local concentration of translation initiation factors [24–26]. We were able to further resolve the *YGR250C* locus to a derived, YJM-specific amino change in a predicted RNA binding motif (**S4 Fig**; **Methods**). As for *IKS1*, this gene encodes an uncharacterized protein kinase that has been shown to be induced during mild heat stress and to alter the sensitivity of yeast to a number of different small molecules [22]. Lastly, *VPS70* encodes an uncharacterized protein involved in vacuolar protein sorting, which is known to mediate cellular response to a wide range of environmental stresses [27–29]. These findings implicate polymorphisms in different components of stress response as major contributors to the heritable growth variation in our study.

### Conclusion

We have determined the genetic basis of an example of GxE in which certain yeast segregants exhibit extremely poor growth in a specific environmental condition. Our results indicate that this poor growth is caused by a number of environmentally responsive loci that individually show additive effects that increase with the severity of environmental stress and collectively result in very poor growth under stressful conditions. This finding provides support for the concept of decanalization, which has been hypothesized to occur when environmental perturbation uncovers sets of deleterious cryptic genetic variants that result in conditional disease phenotypes or other genotype-environment interactions [30]. However, our results are also compatible with recent work illustrating the largely additive genetic basis of quantitative trait variation in yeast [31–33]. Indeed, our work suggests that when many loci show similar gene-environment interactions with environmental stress, decanalization can occur across conditions while trait variation retains an additive genetic architecture within conditions.

The current study also provides a valuable contrast to previous work from our group and others showing a substantial epistatic contribution to GxE [7–9]. Here, we find that epistasis does not meaningfully contribute to GxE in growth variation under our assay conditions. Although we have could have underestimated the contribution of epistasis to our study by focusing on a particular set of four loci, our results might also reflect a major difference in the molecular mechanisms that give rise to the focal phenotypes in the present and past studies. In particular, in previous work on colony morphology [9] and sporulation [8], the examined phenotypes were controlled by specific gene regulatory networks involving multiple polymorphic transcription factors. Genetic variability in such networks is known to be an important source of pairwise and higher-order epistatic interactions [34–39]. In contrast, our current effort is focused on growth, which unlike colony morphology or sporulation, is not a phenotype that arises due to a single predominant gene regulatory network. Thus, our past [9] and current findings suggest that GxE can show a range of genetic architectures from largely additive to largely epistatic. Where the genetic architecture of GxE in a particular trait lies along this spectrum likely depends on the molecular mechanisms that give rise to the phenotype.

## Materials and Methods

### Generation of initial mapping population

Using the synthetic genetic array marker system [40], 112 recombinant BYxYJM *MAT****a*** segregants were generated. The BY parent of our cross was *MATα can1Δ*::*STE2pr-SpHIS5 lyp1Δ his3Δ*, while the YJM parent was *MAT****a*** *his3Δ*::*NatMX ho*::*HphMX*. The BY and YJM haploids were mated to produce a diploid, which was then sporulated using standard techniques [41]. *MAT****a*** segregants were obtained using random spore plating on minimal media containing canavanine, as previously described [36,42].

### Examination of growth among F_2_ segregants

Strains were phenotyped on 2% agar plates containing yeast extract and peptone (YP) with either 2% glucose (dextrose) or 2% ethanol as the carbon source (YPD and YPE, respectively) at 30°C or 37°C. Prior to pinning onto the agar plates, strains were grown overnight to stationary phase in liquid YPD. After this culturing step, strains were then pinned onto agar plates and allowed to grow in the appropriate condition for five days. Individuals were considered poor growing in E37 based on three replicate phenotyping experiments that were performed using randomized designs. Qualitatively poor growth was never observed in G30, G37, or E30.

### Generation of BY and YJM NILs

Similar to our past work [36,43], F_2_B backcross segregants that grow poorly in E37 were obtained by screening haploid progeny from backcrosses of a relevant BYxYJM F_2_ segregant to *MATα ho his3Δ* versions of BY and YJM. Seven BY and seven YJM F_2_Bs were then subjected to five additional rounds of backcrossing with selection for maintenance of poor growth in E37. Each round of backcrossing was performed using *MATα his3Δ* versions of BY and YJM. Sporulation and selection for *MAT****a*** segregants was performed as described for the initial F_2_ population.

### Genotyping of BY and YJM NILs

The NILs were genotyped by Illumina sequencing. Whole genome libraries were constructed using the Illumina Nextera kit, with each library tagged with a unique barcode for multiplexing. Each library was sequenced to an average per site genomic coverage of at least 21X on a NextSeq with 100 base pair (bp) x 100 bp reads. The BY and YJM parent strains were also sequenced to an average per site genomic coverage of ~100X, and these data were used to identify 57,402 high confidence SNPs. Reads for the NILs were mapped to the S288c genome (version S288C_reference_sequence_R64-2-1_20150113.fsa from SGD [http://downloads.yeastgenome.org]) using Burrows-Wheeler Aligner (BWA) version 0.7.7-r441 [44] and mpileup files were generated with SAMTOOLS [45] version 0.1.19-44428cd. The default parameters for BWA and SAMTOOLS were used for mapping Illumina reads to the genome. Genotypes for each individual were called by taking the fraction of BY allele calls at each of the SNPs and employing a Hidden Markov Model by chromosome, using the HMM() package version 1.0 in R, as described in [36]. The parameters used for transition and emission probabilities were transProbs = matrix(c(.9999,.0001,.0001,.9999),2) and emissionProbs = matrix(c(.0.5,0.5,0.5,0.5),2), respectively. We also used the sequencing data to screen the NILs for aneuploidies. If the average sequence coverage for any individual chromosome was 1.5 times higher or lower than the average genome-wide sequencing coverage for a given individual, that strain was classified as aneuploid. Two YJM NILs were found to be aneuploid and thus were excluded from all analyses described in the paper (**S1 Fig**). Additionally, we found that two YJM NILs possessed nearly identical sets of introgressed regions, suggesting a technical error on our part during the recurrent backcrossing process. Only one of these NILs was included in our analyses (**S2 Fig**).

### Generation of YJM NIL 3 F_2_B_7_ segregants

YJM F_2_B_7_ segregants were created by backcrossing YJM NIL 3 (**Fig 3B**) to a *MATα hoΔ his3Δ* version of YJM. Sporulation and selection for *MAT****a*** segregants was performed as described for the initial F_2_ population.

### Genotyping of the F_2_B_7_ population

96 YJM F_2_B_7_ random segregants and 45 additional F_2_B_7_s that grew poorly on E37 were genotyped by sequencing to an average per site coverage of at least 5X using the same method described for the BY and YJM NILs. An additional 96 YJM F_2_B_7_ random segregants were genotyped at the five loci that had been introgressed into YJM NIL 3 using PCR and restriction enzyme typing. All reactions are provided in **S6 Table**. Fisher’s exact tests were then performed in R, using two-by-two matrices in which the first row contained the counts of BY and YJM alleles among the 45 F_2_B_7_s showing poor growth in E37, and the second row contained the counts of BY and YJM alleles among the 192 YJM F_2_B_7_ random population. Allele counts were measured at a single site for each locus that showed maximal allelic enrichment among the 45 F_2_B_7_s that grow poorly in E37.

### Phenotyping of the F_2_B_7_ population

To further analyze growth in the F_2_B_7_ population, we grew each of these individuals on all possible combinations of carbon sources—glucose and ethanol—and temperatures—30 and 37°C. Individuals were pinned onto agar plates and then grown in the appropriate condition for three days. The plates were then imaged using the BioRAD Gel Doc XR+ Molecular Imager. The dimensions of all the images were set at 13.4x10 cm (WxL) and imaged under white Epi illumination with an exposure time of 0.5 seconds. The images were then exported as tiff files with a publishing resolution of 300dpi. To measure the pixel intensity of each colony, ImageJ [46] was used. The total pixel intensity within a circle (spot radius = 50 pixels) surrounding each colony in the image was measured using the Plate Analysis JRU v1 plugin for ImageJ, which was downloaded from the Stowers Institute ImageJ Plugins page (http://research.stowers.org/imagejplugins/index.html; **S5 Fig**). The Circ Background option was used to control for background noise. The average pixel intensity was determined by dividing the total pixel intensity by the area of the circle examined (7845 pixels^2^). Five biological replicate measurements using different randomized designs were taken for each F_2_B_7_ in each condition (**S7 Table**). The median pixel intensity among these five replicates was then used in downstream analyses (**S8 Table**).

### Quantitative analysis of the effect of the causal loci on growth

To measure the additive and epistatic effects of the Chromosome I, VII, X_1, and X_2 loci among the F_2_B_7_s in a particular condition, we implemented full factorial ANOVAs in R. Specifically, we modeled the median pixel intensity of the F_2_B_7_ segregants in each condition as a function of all possible additive and epistatic effects involving the four loci. The model was specified using the statement: lm(median_pixel_intensity_for_each_condition ~ genotype_at_locus_I * genotype_at_locus_VII * genotype_at_locus_X_1 * genotype_at_locus_X_2). ANOVA tables were then obtained using the anova() function. In addition to the terms provided by R, we computed the percent of phenotypic variance explained for each locus by dividing the sum of squares associated with a particular term by the sum of squares total (**Table 1** and **S2**, **S3** and **S4 Tables**). Respectively, the fractions of phenotypic variance explained by all genetic effects (*R*^2^_G_) or only additive genetic effects (*R*^2^_A_) were computed by summing the fractions of phenotypic variance explained by all genetic terms or only additive genetic terms in a given model.

### Modeling of growth as a function of the number of YJM alleles an individual carries

Within each condition, we modeled the median pixel intensities of the F_2_B_7_s as a function of how many YJM alleles they carried. This model assumes complete additivity with loci showing equal effect sizes. These linear models were fit in R using the lm() function in R with the statement lm(median_pixel_intensity_for_each_condition ~ number_of_YJM_alleles_at_four_loci).

### Genetic engineering

All transformations were conducted using standard PCR-based techniques [47]. Allele replacement strains were constructed using the co-transformation of two partially overlapping PCR products as described in [43]. One product contained the promoter and coding region of the gene to be replaced, while the other included (in order) 60 bp of overlap with the 3’ end of the gene PCR product, *kanMX*, and 60 bp of the genomic region downstream of the transcribed portion of the gene, such that the entire coding and the promoter region of a given gene was replaced (**S9 Table**). All engineerings were performed in YJM NIL 3 and involved replacement of the BY allele of a given gene with the YJM allele. Each putative allele replacement was verified by Sanger sequencing. Controls were also generated to ensure that inserting *kanMX* near each gene was not responsible for our findings.

### Population, phylogenetic, and functional analysis of the causal polymorphism in *YGR250C*

DNA sequences for other *S*. *cerevisiae* strains were downloaded from the *Saccharomyces* Genome Database (http://www.yeastgenome.org; **S10 Table**), as well as from different *S*. *cerevisiae* resequencing projects [10,48]. DNA sequence alignments were then generated using Geneious v7.0.6 and the amino acid sequences of these other isolates was determined by translating the DNA sequence alignment. The amino acid sequences of other closely related fungal species were obtained using WU-BLAST2 with default settings (http://www.yeastgenome.org/blast-fungal). The putative RNA binding motifs of *YGR250C* were then identified from domain predictions available through InterPro (http://www.ebi.ac.uk/interpro/protein/P53316) [49].

## Supporting Information

S1 Fig Whole genome sequencing reveals two YJM NILs are aneuploid. (TIF)

S2 Fig YJM NIL 2 and another YJM NIL show similar introgressed genomic regions. One YJM NIL, which is denoted as YJM NIL 2*, was excluded from further study as it appears to be a replicate of YJM NIL 2.
(TIF)

S3 Fig Four of the five introgressed genomic regions in YJM NIL 3 contribute to poor growth in E37. Frequencies of the BY alleles at each locus in the populations of poorly growing and control F_2_B_7_s are plotted. The Chromosome I, VII, X_1, and X_2 loci show statistically significant differences in their frequencies between the two populations (Fisher’s exact tests: I: *p* ≤ 3.84 x 10^−8^, VII: *p* ≤ 3.98 x 10^−20^, X_1: *p* ≤ 8.38 x 10^−7^, X_2: *p* ≤ 1.56 x 10^−20^), while the locus on Chromosome XVI did not (XVI: p ≤ 0.341). The significant loci are denoted with ‘***’.
(TIF)

S4 Fig *YGR250C*^YJM^ contains an amino acid change in a highly conserved site. (**A**) Amino acid differences between BY and YJM are shown with either a black line (non-causal) or a red line (causal). The three predicted RNA recognition motifs within *YGR250C* are labeled in purple. (**B**) The causal amino acid polymorphism in YJM is highlighted in red and other sites that differ from *S*. *cerevisiae* are highlighted in grey. Based on presently available genomes from recent resequencing projects [10,48] or the Saccharomyces Genome Database [22], YJM is the only budding yeast that harbors an amino acid at position 542 that is not a leucine or an isoleucine.
(TIF)

S5 Fig Sample image of how growth in the F_2_B_7_ population was measured using the Plate Analysis plugin for ImageJ. (TIF)

S1 Table Genomic intervals that were introgressed in at least 2 NILs. (DOCX)

S2 Table Full factorial ANOVA for G30 condition. (DOCX)

S3 Table Full factorial ANOVA for E30 condition. (DOCX)

S4 Table Full factorial ANOVA for G37 condition. (DOCX)

S5 Table Genetic intervals identified in the 45 F_2_B_7_s segregants with poor growth in E37. (DOCX)

S6 Table PCR primers and restriction enzymes used for genotyping F_2_B_7_s. (DOCX)

S7 Table Pixel intensity measurements and genotypes of F_2_B_7_s. Column 2 shows the replicate number, and Columns 3 through 6 labeled ‘G30’,’G37’,’E30’, and ‘E37’ are the average pixel intensity of the colonies measured under the four conditions. The genotype at a locus is labeled as ‘0’ if the individual carries the BY allele or ‘1’ if it carries the YJM allele.
(XLS)

S8 Table Median pixel intensity measurements of F_2_B_7_s and their genotypes at the four loci. Columns 2 through 5 labeled ‘G30_med’, ‘G37_med’, ‘E30_med’, and ‘E37_med’ are the median pixel intensity measurements from the five replicates, which were used in the analyses described in the paper. The genotype at the four loci are labeled as ‘0’ if the individual carries the BY allele or ‘1’ if it carries the YJM allele.
(XLSX)

S9 Table PCR primers used to make replacement strains in YJM NIL 3. The first 11 rows show how the primes were designed to make allele replacement primers. Base pairs shown in black are sequences found in the *kanMX* drug resistance cassette, which is used for drug selection and are same in all of the PCR primers. Base pairs shown in green varies depends on which genes are being replaced. For allele replacements on the positive strand, primers labeled GeneX MAR1 and GeneX MAR2 were used to amplify the promoter and the coding region of the gene being replaced with a 60bp tail overlapping the first 60bp of the *kanMX* cassette. *KanMX* amplification primer 3 and GeneX MAR4 were used to amplify the *kanMX* cassette with a 60 bp tail that is homologous to the genomic region downstream of the transcribed portion of the gene. The two PCR products were then co-transformed into the yeast cell and selected for G418 resistance. For allele replacements on the negative strand, primers labeled GeneX MAR1 and *kanMX* amplification primer 2 were used to amplify the *kanMX* cassette with a 60 bp tail that is homologous to the genomic region downstream of the transcribed portion of the gene. GeneX MAR3 and GeneX MAR4 were used to amplify the promoter and the coding region of the gene being replaced with a 60bp tail overlapping the last 60bp of the *kanMX* cassette. Expected PCR amplicons are provided in **S2 Note**.
(XLSX)

S10 Table Strain name and the genome version used for study in this paper. All the genomes were downloaded from SGD.
(XLSX)

S1 Note Attempt to clone causal gene underlying the Chromosome I locus. (DOCX)

S2 Note Sequences amplified by the PCR primers described in S9 Table. Sequences corresponding to the target locus are in green, while sequences corresponding to *kanMX* are in black.
(DOCX)
