# Supplementary figures and images for: Gene-Environment Interactions in Stress Response Contribute Additively to a Genotype-Environment Interaction
Source: PLoS Genet. 2016 Jul 20;12(7):e1006158. doi: 10.1371/journal.pgen.1006158 (PMC4954657; doi:10.1371/journal.pgen.1006158)

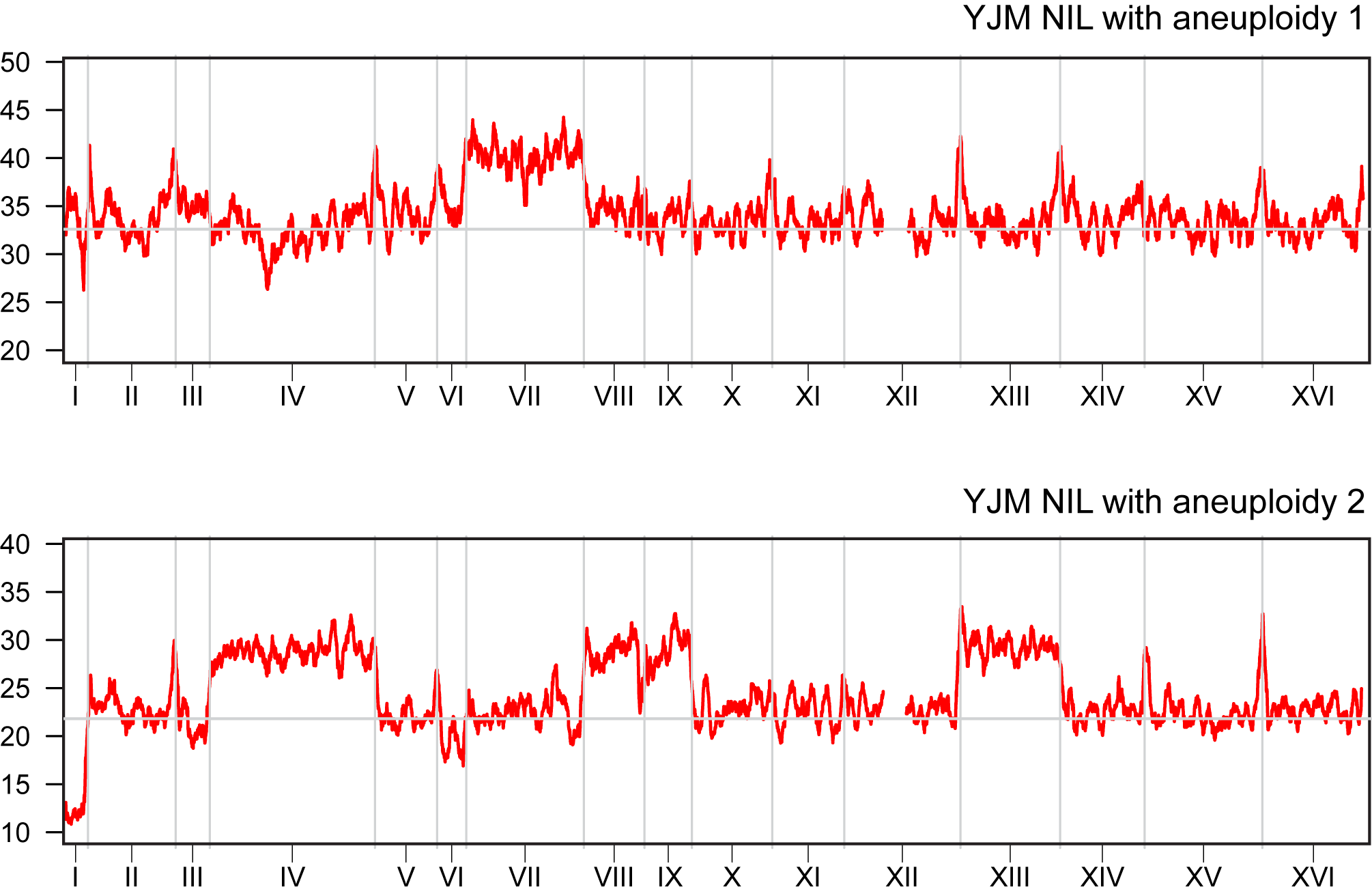

Supplement: S1 Fig — (TIF) [file pgen.1006158.s001.tif]

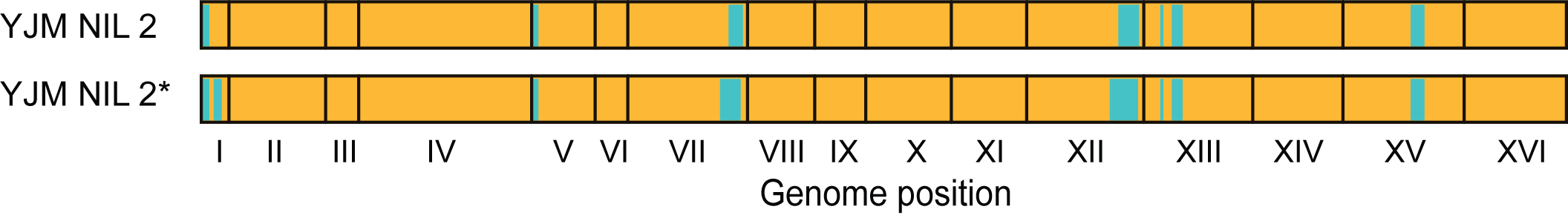

Supplement: S2 Fig — One YJM NIL, which is denoted as YJM NIL 2*, was excluded from further study as it appears to be a replicate of YJM NIL 2. (TIF) [file pgen.1006158.s002.tif]

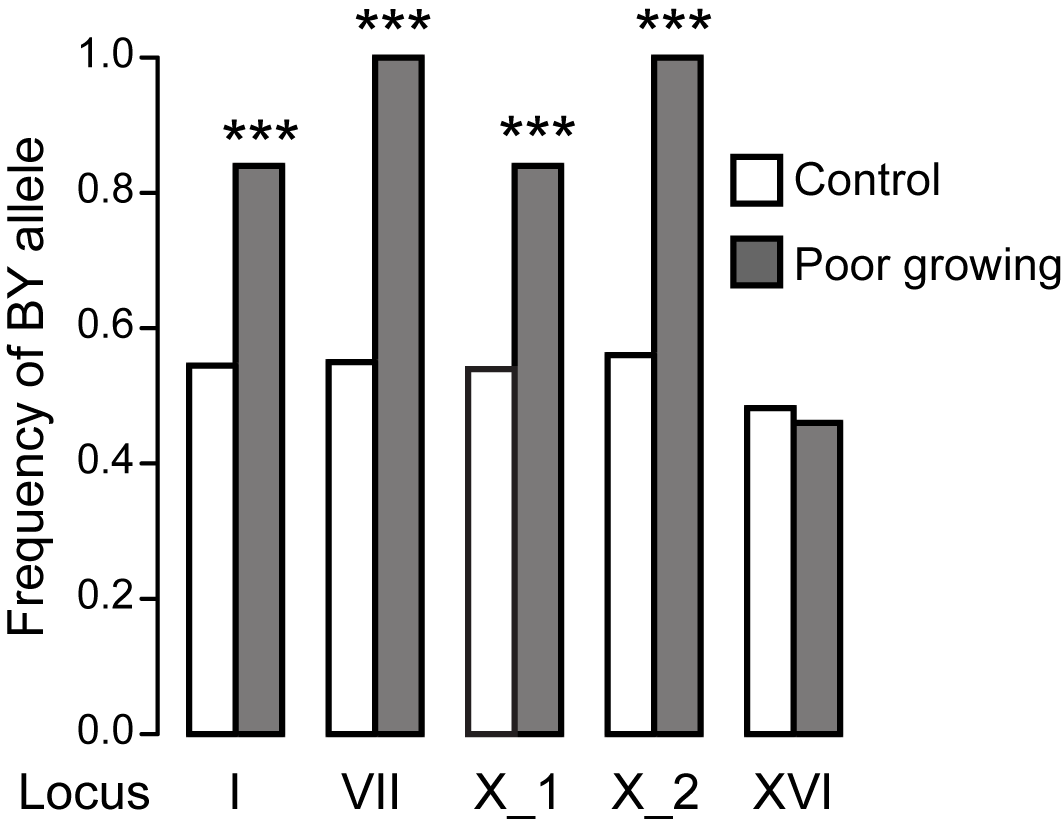

Supplement: S3 Fig — Frequencies of the BY alleles at each locus in the populations of poorly growing and control F2B7s are plotted. The Chromosome I, VII, X_1, and X_2 loci show statistically significant differences in their frequencies between the two populations (Fisher’s exact tests: I: p ≤ 3.84 x 10^−8^, VII: p ≤ 3.98 x 10^−20^, X_1: p ≤ 8.38 x 10^−7^, X_2: p ≤ 1.56 x 10^−20^), while the locus on Chromosome XVI did not (XVI: p ≤ 0.341). The significant loci are denoted with ‘***’. (TIF) [file pgen.1006158.s003.tif]

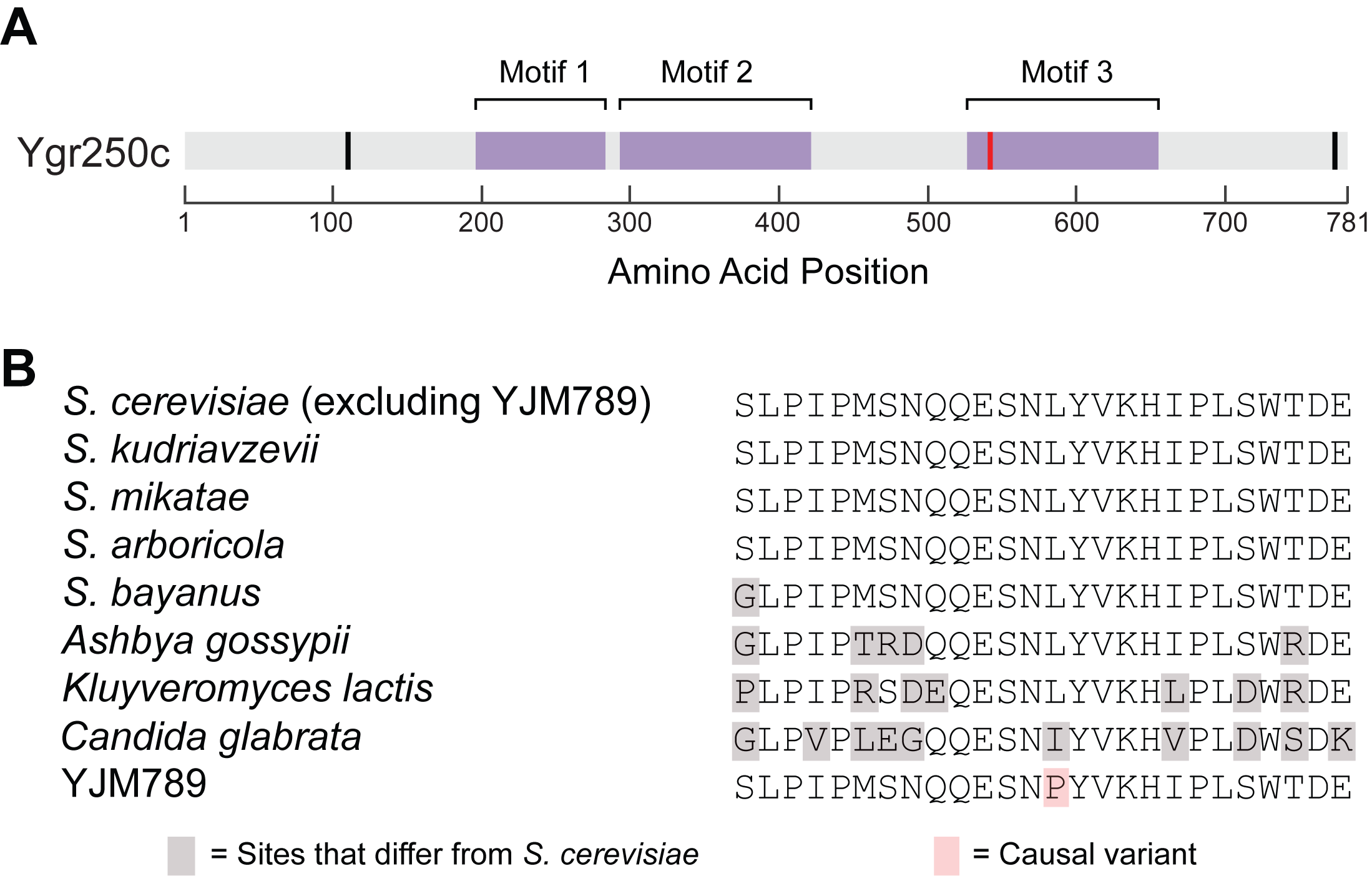

Supplement: S4 Fig — (A) Amino acid differences between BY and YJM are shown with either a black line (non-causal) or a red line (causal). The three predicted RNA recognition motifs within YGR250C are labeled in purple. (B) The causal amino acid polymorphism in YJM is highlighted in red and other sites that differ from S. cerevisiae are highlighted in grey. Based on presently available genomes from recent resequencing projects [10,48] or the Saccharomyces Genome Database [22], YJM is the only budding yeast that harbors an amino acid at position 542 that is not a leucine or an isoleucine. (TIF) [file pgen.1006158.s004.tif]

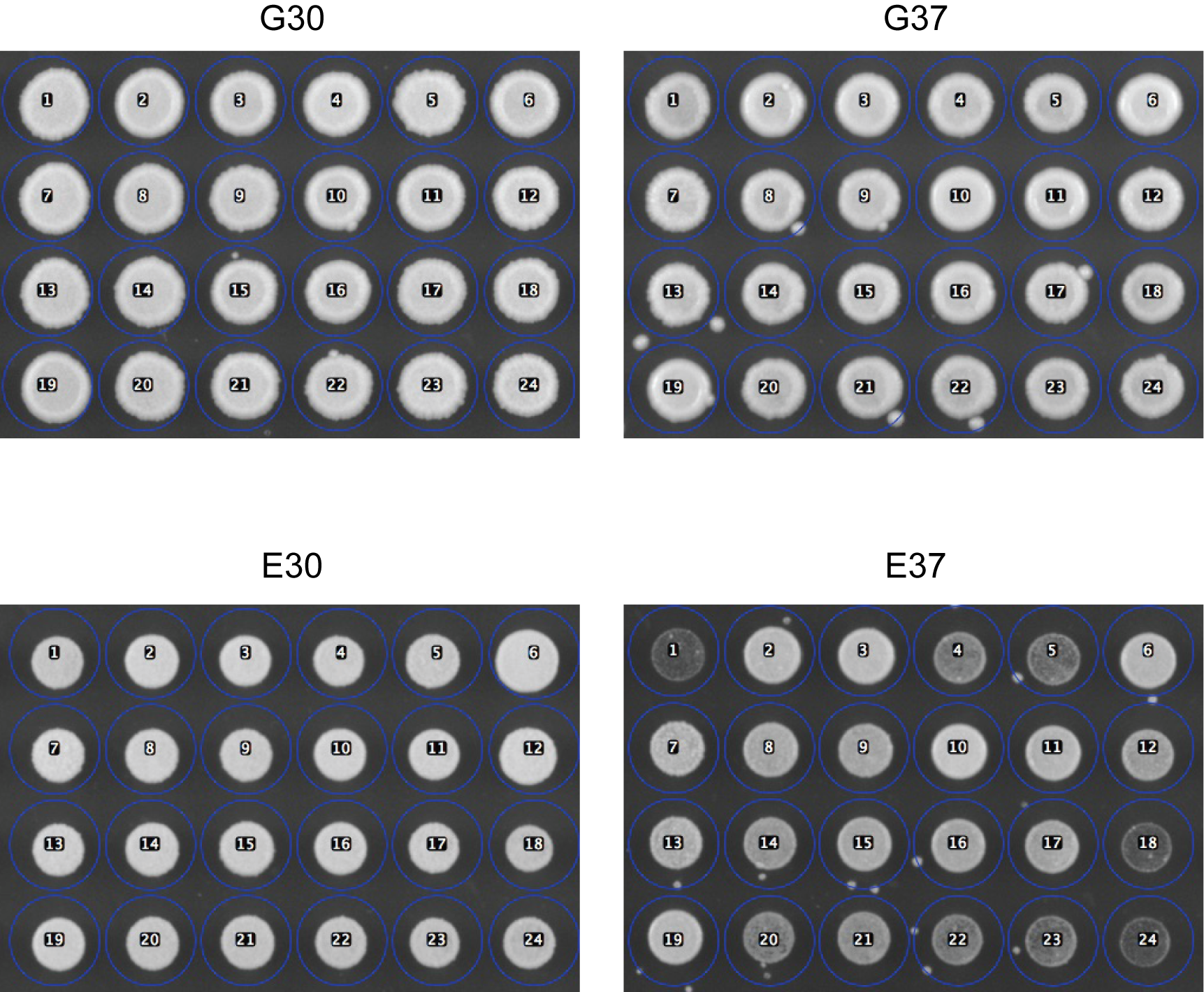

Supplement: S5 Fig — (TIF) [file pgen.1006158.s005.tif]
