# Supplementary material for: Gene-Environment Interactions in Stress Response Contribute Additively to a Genotype-Environment Interaction
Source: PLoS Genet. 2016 Jul 20;12(7):e1006158. doi: 10.1371/journal.pgen.1006158 (PMC4954657; doi:10.1371/journal.pgen.1006158)
Supplement: S1 Table — (DOCX) [file pgen.1006158.s006.docx]

**S1 Table. Genomic intervals that were introgressed in at least 2 NILs**

| **Contributing parent** | **Chromosome** | **Start Position** | **End Position** |
| --- | --- | --- | --- |
| BY | V-1 | 97416 | 208850 |
| BY | V-2 | 361243 | 371216 |
| BY | XI | 617954 | 632869 |
| BY | XIII-1 | 103752 | 116112 |
| BY | XIII-2 | 409643 | 434776 |
| BY | XIII-3 | 817457 | 864535 |
| BY | XIV-1 | 196326 | 242111 |
| BY | XIV-2 | 349812 | 356084 |
| BY | XV | 388886 | 459980 |
| YJM | I | 35751 | 58166 |
| YJM | IV | 928114 | 997571 |
| YJM | VII | 956516 | 1009525 |
| YJM | X-1 | 237551 | 363087 |
| YJM | X-2 | 609577 | 673602 |
| YJM | XII | 967268 | 1001139 |
| YJM | XV | 585644 | 638733 |
