# Supplementary material for: Gene-Environment Interactions in Stress Response Contribute Additively to a Genotype-Environment Interaction
Source: PLoS Genet. 2016 Jul 20;12(7):e1006158. doi: 10.1371/journal.pgen.1006158 (PMC4954657; doi:10.1371/journal.pgen.1006158)
Supplement: S2 Table — (DOCX) [file pgen.1006158.s007.docx]

**S2 Table. Full factorial ANOVA for G30 condition.**

| **Source** | **Df** | **Sum Sq** | **Mean Sq** | **F value** | **Pr(>F)** | **PVE** |
| --- | --- | --- | --- | --- | --- | --- |
| I | 1 | 114.5 | 114.54 | 2.0715 | 0.15186 | 1.1 |
| VII | 1 | 21.6 | 21.64 | 0.3914 | 0.53236 | 0.2 |
| X_1 | 1 | 25.2 | 25.17 | 0.4552 | 0.50076 | 0.2 |
| X_2 | 1 | 44.6 | 44.63 | 0.8072 | 0.37018 | 0.4 |
| I:VII | 1 | 4.3 | 4.33 | 0.0784 | 0.77985 | 0 |
| I:X_1 | 1 | 4.3 | 4.26 | 0.0771 | 0.78163 | 0 |
| VII:X_1 | 1 | 171.3 | 171.35 | 3.0989 | 0.08009 | 1.6 |
| I:X_2 | 1 | 23.7 | 23.74 | 0.4293 | 0.5132 | 0.2 |
| VII:X_2 | 1 | 14.7 | 14.68 | 0.2656 | 0.60698 | 0.1 |
| X_1:X_2 | 1 | 0.1 | 0.13 | 0.0023 | 0.96178 | 0 |
| I:VII:X_1 | 1 | 2.9 | 2.92 | 0.0527 | 0.81862 | 0 |
| I:VII:X_2 | 1 | 26.9 | 26.93 | 0.487 | 0.48618 | 0.3 |
| I:X_1:X_2 | 1 | 73.9 | 73.93 | 1.3371 | 0.24912 | 0.7 |
| VII:X_1:X_2 | 1 | 14.3 | 14.27 | 0.258 | 0.61212 | 0.1 |
| I:VII:X_1:X_2 | 1 | 351.8 | 351.84 | 6.363 | 0.01254 | 3.3 |
| Residuals | 175 | 9676.4 | 55.29 |  |  |  |
