# Supplementary material for: Gene-Environment Interactions in Stress Response Contribute Additively to a Genotype-Environment Interaction
Source: PLoS Genet. 2016 Jul 20;12(7):e1006158. doi: 10.1371/journal.pgen.1006158 (PMC4954657; doi:10.1371/journal.pgen.1006158)
Supplement: S3 Table — (DOCX) [file pgen.1006158.s008.docx]

**S3 Table. Full factorial ANOVA for E30 condition.**

| **Source** | **Df** | **Sum Sq** | **Mean Sq** | **F value** | **Pr(>F)** | **PVE** |
| --- | --- | --- | --- | --- | --- | --- |
| I | 1 | 633.57 | 633.57 | 43.2163 | 5.435e-10 | 13.3 |
| VII | 1 | 727.81 | 727.81 | 49.644 | 4.068e-11 | 15.3 |
| X_1 | 1 | 264.69 | 264.69 | 18.0548 | 3.483e-05 | 5.6 |
| X_2 | 1 | 126.27 | 126.27 | 8.6129 | 0.0037859 | 2.7 |
| I:VII | 1 | 49.21 | 49.21 | 3.3569 | 0.0686245 | 1.0 |
| I:X_1 | 1 | 14.74 | 14.74 | 1.0055 | 0.3173638 | 0.3 |
| VII:X_1 | 1 | 227.52 | 227.52 | 15.5191 | 0.0001178 | 4.8 |
| I:X_2 | 1 | 0.14 | 0.14 | 0.0092 | 0.9235038 | 0 |
| VII:X_2 | 1 | 13.24 | 13.24 | 0.903 | 0.343296 | 0.3 |
| X_1:X_2 | 1 | 0.34 | 0.34 | 0.0234 | 0.8787173 | 0 |
| I:VII:X_1 | 1 | 18.75 | 18.75 | 1.2791 | 0.2596155 | 0.4 |
| I:VII:X_2 | 1 | 53.92 | 53.92 | 3.678 | 0.0567623 | 1.1 |
| I:X_1:X_2 | 1 | 38.06 | 38.06 | 2.5961 | 0.1089287 | 0.8 |
| VII:X_1:X_2 | 1 | 17.01 | 17.01 | 1.1606 | 0.2828289 | 0.4 |
| I:VII:X_1:X_2 | 1 | 0.4 | 0.4 | 0.0275 | 0.8684786 | 0 |
| Residuals | 175 | 2565.59 | 14.66 |  |  |  |
