# Supplementary material for: Gene-Environment Interactions in Stress Response Contribute Additively to a Genotype-Environment Interaction
Source: PLoS Genet. 2016 Jul 20;12(7):e1006158. doi: 10.1371/journal.pgen.1006158 (PMC4954657; doi:10.1371/journal.pgen.1006158)
Supplement: S4 Table — (DOCX) [file pgen.1006158.s009.docx]

**S4 Table. Full factorial ANOVA for G37 condition.**

| **Source** | **Df** | **Sum Sq** | **Mean Sq** | **F value** | **Pr(>F)** | **PVE** |
| --- | --- | --- | --- | --- | --- | --- |
| I | 1 | 829.1 | 829.05 | 15.0553 | 0.0001477 | 6.4 |
| VII | 1 | 797.3 | 797.29 | 14.4786 | 0.0001958 | 6.1 |
| X_1 | 1 | 802.8 | 802.84 | 14.5792 | 0.0001863 | 6.2 |
| X_2 | 1 | 404.2 | 404.18 | 7.3398 | 0.0074147 | 3.1 |
| I:VII | 1 | 1.2 | 1.19 | 0.0216 | 0.8833552 | 0 |
| I:X_1 | 1 | 49.6 | 49.57 | 0.9001 | 0.3440591 | 0.4 |
| VII:X_1 | 1 | 66.5 | 66.48 | 1.2073 | 0.2733816 | 0.5 |
| I:X_2 | 1 | 46.1 | 46.1 | 0.8372 | 0.3614623 | 0.4 |
| VII:X_2 | 1 | 5.9 | 5.94 | 0.1078 | 0.7430638 | 0 |
| X_1:X_2 | 1 | 125.3 | 125.31 | 2.2756 | 0.1332302 | 1.0 |
| I:VII:X_1 | 1 | 21.8 | 21.8 | 0.3958 | 0.5300677 | 0.2 |
| I:VII:X_2 | 1 | 8.6 | 8.58 | 0.1558 | 0.6935174 | 0.1 |
| I:X_1:X_2 | 1 | 0.1 | 0.08 | 0.0015 | 0.968715 | 0 |
| VII:X_1:X_2 | 1 | 3.3 | 3.33 | 0.0605 | 0.8060051 | 0 |
| I:VII:X_1:X_2 | 1 | 210.6 | 210.64 | 3.8252 | 0.0520784 | 1.6 |
| Residuals | 175 | 9636.7 | 55.07 |  |  |  |
