# Supplementary material for: Gene-Environment Interactions in Stress Response Contribute Additively to a Genotype-Environment Interaction
Source: PLoS Genet. 2016 Jul 20;12(7):e1006158. doi: 10.1371/journal.pgen.1006158 (PMC4954657; doi:10.1371/journal.pgen.1006158)
Supplement: S5 Table — (DOCX) [file pgen.1006158.s010.docx]

**S5 Table. Genomic intervals identified in the 45 F_2_B_7_s segregants with poor growth in E37.**

| Chromosome | Start position | End Position | Causal Gene |
| --- | --- | --- | --- |
| I | 52603 | 53266 | ? |
| VII | 987973 | 996630 | *YGR250C* |
| X_1 | 326422 | 329730 | *IKS1* |
| X_2 | 651274 | 662418 | *VPS70* |
