# Supplementary material for: Gene-Environment Interactions in Stress Response Contribute Additively to a Genotype-Environment Interaction
Source: PLoS Genet. 2016 Jul 20;12(7):e1006158. doi: 10.1371/journal.pgen.1006158 (PMC4954657; doi:10.1371/journal.pgen.1006158)
Supplement: S6 Table — (DOCX) [file pgen.1006158.s011.docx]

| **Introgressed Loci** | **Primer Sequence** | **Restriction enzyme used** | **Allele that is cut** |
| --- | --- | --- | --- |
| Chr I F primer | TGATATGTTTGGTTTTGCTTATAGA | HpyCH4III | BY |
| Chr I R primer | AAGGTTGGGGTACGAATTGC | HpyCH4III | BY |
| Chr VII F primer | AATGTCCCAGATGGTTCTGC | MnlI | BY |
| Chr VII R primer | TGATTGAACATGCGCGTACT | MnlI | BY |
| Chr X-1 F primer | CCAAAGTTGTTTTCTTAATCATCGT | KpnI | BY |
| Chr X-1 R primer | AAGGAAAGCGTTGAAAAGCA | KpnI | BY |
| Chr X-2 F primer | CCAATCTTTGTTGCTCACACC | BanI | YJM |
| Chr X-2 R primer | GACACACGAGGAAGTACAACCA | BanI | YJM |
| Chr XVI F primer | GGGGCGCTCTTGTATAAGTAA | BslI | YJM |
| Chr XVI R primer | ACAACTACGGTGGCCATACC | BslI | YJM |

**S6 Table. PCR primers and restriction enzymes used for genotyping F2B7s.**
