# Supplementary material for: Gene-Environment Interactions in Stress Response Contribute Additively to a Genotype-Environment Interaction
Source: PLoS Genet. 2016 Jul 20;12(7):e1006158. doi: 10.1371/journal.pgen.1006158 (PMC4954657; doi:10.1371/journal.pgen.1006158)
Supplement: S1 Note — (DOCX) [file pgen.1006158.s016.docx]

**S1 Note. Attempt to clone causal gene underlying the Chromosome I locus.**

Even though we were able to resolve the genomic interval for the Chromosome I locus down to ~600bp containing one gene, *GEM1,* the replacement of this gene with the YJM allele in the YJM NIL3 genetic background did not restore growth (**S10 Table**). To identify the causal gene, we next expanded our search to genomic intervals where the BY allele was second and third most enriched in the 45 F_2_B_7_s segregants with poor growth in E37. This decreased the resolution to ~19kb, containing 8 genes (**S10 Table**). We again tried replacing all the genes in the interval. However, none of the replacements had any effect on growth. This failure to resolve the causal gene could be caused by several reasons. One possibility is that we may need to expand our search even further. Another possibility is that there may be multiple genes with effects on growth in the Chromosome I locus. In this case, replacement of one gene may not be sufficient to restore growth. A further possibility is that the marked allele replacement strategy we employed disrupted a functional element necessary for detecting the effect of the causal polymorphism in the Chromosome I locus, such as a 3’ UTR or a transcription factor binding site.
