# Supplementary material for: Gene-Environment Interactions in Stress Response Contribute Additively to a Genotype-Environment Interaction
Source: PLoS Genet. 2016 Jul 20;12(7):e1006158. doi: 10.1371/journal.pgen.1006158 (PMC4954657; doi:10.1371/journal.pgen.1006158)
Supplement: S2 Note — Sequences corresponding to the target locus are in green, while sequences corresponding to kanMX are in black. (DOCX) [file pgen.1006158.s017.docx]

**S2 Note.** PCR products of the primers shown in table S9.

*GEM1* MAR 1and *kanMX* amplification primer 2:

AGTGGAAAACTTTAAGTCTACTGAGTCTTGAGACCTTATCACCCTTTGAAGGTTTCCTTGACAGTCTTGACGTGCGCAGCTCAGGGGCATGATGTGACTGTCGCCCGTACATTTAGCCCATACATCCCCATGTATAATCATTTGCATCCATACATTTTGATGGCCGCACGGCGCGAAGCAAAAATTACGGCTCCTCGCTGCAGACCTGCGAGCAGGGAAACGCTCCCCTCACAGACGCGTTGAATTGTCCCCACGCCGCGCCCCTGTAGAGAAATATAAAAGGTTAGGATTTGCCACTGAGGTTCTTCTTTCATATACTTCCTTTTAAAATCTTGCTAGGATACAGTTCTCACATCACATCCGAACATAAACAACCATGGGTAAGGAAAAGACTCACGTTTCGAGGCCGCGATTAAATTCCAACATGGATGCTGATTTATATGGGTATAAATGGGCTCGCGATAATGTCGGGCAATCAGGTGCGACAATCTATCGATTGTATGGGAAGCCCGATGCGCCAGAGTTGTTTCTGAAACATGGCAAAGGTAGCGTTGCCAATGATGTTACAGATGAGATGGTCAGACTAAACTGGCTGACGGAATTTATGCCTCTTCCGACCATCAAGCATTTTATCCGTACTCCTGATGATGCATGGTTACTCACCACTGCGATCCCCGGCAAAACAGCATTCCAGGTATTAGAAGAATATCCTGATTCAGGTGAAAATATTGTTGATGCGCTGGCAGTGTTCCTGCGCCGGTTGCATTCGATTCCTGTTTGTAATTGTCCTTTTAACAGCGATCGCGTATTTCGTCTCGCTCAGGCGCAATCACGAATGAATAACGGTTTGGTTGATGCGAGTGATTTTGATGACGAGCGTAATGGCTGGCCTGTTGAACAAGTCTGGAAAGAAATGCATAAGCTTTTGCCATTCTCACCGGATTCAGTCGTCACTCATGGTGATTTCTCACTTGATAACCTTATTTTTGACGAGGGGAAATTAATAGGTTGTATTGATGTTGGACGAGTCGGAATCGCAGACCGATACCAGGATCTTGCCATCCTATGGAACTGCCTCGGTGAGTTTTCTCCTTCATTACAGAAACGGCTTTTTCAAAAATATGGTATTGATAATCCTGATATGAATAAATTGCAGTTTCATTTGATGCTCGATGAGTTTTTCTAATCAGTACTGACAATAAAAAGATTCTTGTTTTCAAGAACTTGTCATTTGTATAGTTTTTTTATATTGTAGTTGTTCTATTTTAATCAAATGTTAGCGTGATTTATATTTTTTTTCGCCTCGACATCATCTGCCCAGATGCGAAGTTAAGTGCG

*GEM1* MAR 3 and *GEM1* MAR 4:

AGCGTGATTTATATTTTTTTTCGCCTCGACATCATCTGCCCAGATGCGAAGTTAAGTGCGCTTGCAAATGAGCGTGGTTTGGCATTTTTTATCGGAAAGAAAAAAgGGGCTCCGCCTTAGGCCAGATATCATAGAAATGCAACACTTCCCTAATATAGAAATTTGGGCATTAATTATTTTGAGAATTTTGATGATTTGAATAATTTCATTAACGTAAAaGAgCATAGTGCcACGAATCCAACAGTGGACCCAAAAATGAGAGCCGTTTGTCTGTAGTCGACATCTTTTGCTGCTGTTTCTTCTGGCAATCCtGGAGTGTTTTTtCCAGGATCAAGAGCAGCTTCTGTGATTTTAATgAACAATTCATTgAGGGAACTTAGCCATCTGGATGATATGTGCAGTGGGTGGTTCACAAATAGCTCGTCTGCCAGTTCATCTGGTTGGATTTGACACCTTTGTTGCTGCTTATCCAAATCTGCCTTAGAAGCTACAAATACCAACGGTAGATCTTGTAAATGgGTGAATTTGTCTAGAAGCGAgACTAAGTAGGAGAATGATTCTGGGTCGCTGGAATCGTATGTTAGACAGATTACGTCACATTCTTTTAACTTATCCTTATTCTCTAGTATGGCGTATTCtTGTTCTCCAAGTTCTTGCAAAATCAAATAGTACTGTTTCCCACCTTTGAGTTCTAAACTATTGACTGCAATTCTTGGTTTGATTGTCGGAGAATACTCCTCCGAGAAAGATCTGCCCAAGAAGGCCTCTAGCAAAGAGCTTTTGCCGCAACATGGCTTTCCAATGACAAAGCAATTGAACACTTTTCTGTCATTGATATTGGATCTGTAAAGTTTCCCGGAACGGCGTCTCATTTTCCTTGGCTTGGTTACTTGTAGGGCTAGTCTTGCATCTTCtTGAAAGCCAAAATACACCAAGTAAGCGGTAGTTGTGCTATAGTTCAAGAAAGTCGTCATACTCCATTGTGCTAGCCAGCCTTGTAAGGTGATGCAACCCTTGTTGTTTACGACAGTGGAGAAGGGGAAATTCGTTGAGGTCCATAGTTTAGGCAGCCCTGGTGTGCACTTAAATAGACGATGTAATTCTTGATTATTCAAACCACCATCATTGTCGATATCAAACTTCAAAAAAATATCTACAAGAAATCTGTAGCCCTTGGGGCTCAATTCCACACTGGAAGTGTCAGGGACgACCAACtTCGGATGaAGAATTTTGTCATTAATACACAAGGAATCTGTGTAATGGAAAGTTCTTAGGATAGCCCATGTAGTTTCGTGTCTCCCCCTTTCtGCGTATATTTTGTTCAGTACAAGGAAACCATCTTTGGTGATGCCTTTTCCCGGTACGTATAGCTTGCGGTTAATGTACTCTTGATCGTGCTTGGAAATATCCAAAAGCAAATCTTTAATAAAATTCAGTTCGTTTACATCGATACTCTTATTGAAGCACTTTTTTTGTAAGCCCAAGATTTCGTTGTCATCTAAATATGAGTCCTGGTTTAAATCGCTTAAAAGAAAAATTCTTTTTAAgGCCATGACAGCCAATGGCTTTAaTTCACCTACCATGGCATCAAATAAAGGTGATATTGGGTGTGTTATgGCCCTTTGGCAAAGATAAAACGCTTGGTTAAGATCAAACTGTGTCTTGGCACTTGTCTTAATGCAAGTGTCGATcTCTTTAAACTCCATTAATATTGGGATAAATTCTTCATCCTCCACTTTGGTATCGATATCATCATCACTGTTCTCTGACACGACCATTGCATTGGCATTAACATTCGATATGGAATCACATTTATTTTTGCAGAGAATGACAGGAATATTCAACCCCAGaGAcCTGAAATGAGGCAACCAAAAGAGAGAgACATGGTCATACGATTCGTGATCGCAATACACAAGCCAAATTACGTCGGCGGACTTCAACTCATGGTCTAAAGCTATGAGGTCCGAATCTGAAGTGTCTATAAGTACTGTATTCTTAGGAGAATATGTAGGTGATGATGAGAAATCTCTTGGtATACTGATGGGTGGCAGtACGTCCTGTATGGTCGGTATGAATTCAGCTTTTGTTAATGATACAATCAGACTGGATTTACCcACCCCTTCATCACCGCAAATAACTACCCGAATCGTTTCTTTgGTCATTGTGTTGTTCAACACAcTAGTATTTAGAAGTCCGCTATTTTTGTTTTCA

*SPC72* MAR 1and *kanMX* amplification primer 2:

GTTTTGAGGGTAAACCACTATACCAAATGTTGAAGATCTAAAGGTATCGATCAAATATGTCCTTGACAGTCTTGACGTGCGCAGCTCAGGGGCATGATGTGACTGTCGCCCGTACATTTAGCCCATACATCCCCATGTATAATCATTTGCATCCATACATTTTGATGGCCGCACGGCGCGAAGCAAAAATTACGGCTCCTCGCTGCAGACCTGCGAGCAGGGAAACGCTCCCCTCACAGACGCGTTGAATTGTCCCCACGCCGCGCCCCTGTAGAGAAATATAAAAGGTTAGGATTTGCCACTGAGGTTCTTCTTTCATATACTTCCTTTTAAAATCTTGCTAGGATACAGTTCTCACATCACATCCGAACATAAACAACCATGGGTAAGGAAAAGACTCACGTTTCGAGGCCGCGATTAAATTCCAACATGGATGCTGATTTATATGGGTATAAATGGGCTCGCGATAATGTCGGGCAATCAGGTGCGACAATCTATCGATTGTATGGGAAGCCCGATGCGCCAGAGTTGTTTCTGAAACATGGCAAAGGTAGCGTTGCCAATGATGTTACAGATGAGATGGTCAGACTAAACTGGCTGACGGAATTTATGCCTCTTCCGACCATCAAGCATTTTATCCGTACTCCTGATGATGCATGGTTACTCACCACTGCGATCCCCGGCAAAACAGCATTCCAGGTATTAGAAGAATATCCTGATTCAGGTGAAAATATTGTTGATGCGCTGGCAGTGTTCCTGCGCCGGTTGCATTCGATTCCTGTTTGTAATTGTCCTTTTAACAGCGATCGCGTATTTCGTCTCGCTCAGGCGCAATCACGAATGAATAACGGTTTGGTTGATGCGAGTGATTTTGATGACGAGCGTAATGGCTGGCCTGTTGAACAAGTCTGGAAAGAAATGCATAAGCTTTTGCCATTCTCACCGGATTCAGTCGTCACTCATGGTGATTTCTCACTTGATAACCTTATTTTTGACGAGGGGAAATTAATAGGTTGTATTGATGTTGGACGAGTCGGAATCGCAGACCGATACCAGGATCTTGCCATCCTATGGAACTGCCTCGGTGAGTTTTCTCCTTCATTACAGAAACGGCTTTTTCAAAAATATGGTATTGATAATCCTGATATGAATAAATTGCAGTTTCATTTGATGCTCGATGAGTTTTTCTAATCAGTACTGACAATAAAAAGATTCTTGTTTTCAAGAACTTGTCATTTGTATAGTTTTTTTATATTGTAGTTGTTCTATTTTAATCAAATGTTAGCGTGATTTATATTTTTTTTCGCCTCGACATCATCTGCCCAGATGCGAAGTTAAGTGCG

*SPC72* MAR 3 and *SPC72* MAR 4:

AGCGTGATTTATATTTTTTTTCGCCTCGACATCATCTGCCCAGATGCGAAGTTAAGTGCG**TGCTAGAGAGTGACTGAGTGTTACATTAAATATATTTATATATAAACGTATGATATTTAGGGATTGTTGATTGATAGGTTGAAAAGTTTCGATCTCAATGACTCATTTTCCTGTTCTAAAGCCTTGATTCGAGCTTCTGAAGCGTTAGCATCGAGCTTCCGTCTTTCTCTTTCGGATATCCATCTTCTTTGTAACTCCTCTATTCGTAAGGTtAGTTCTTTATTTGGAGTTGCCGTTAgTTCATTGCCCTGTTGATGTGGCTGCTCTGGGGTTTCCATGGcAATCAGTGAAGAGATATATGAGTTTATTATGGACTCTAATGCgGTCTCTATAAAAGTGTAGAGCGATTCTAGTTTGGGCTGAATCAAATTCAAGTTTTTCAACGCATTTGGTAaAGATTTTATGGATTTCATTTTCCTGTCAAATTGGGCAATAGAACTTTCTTGTAAGATTTTCTGCAGAATATTGAAAATACTGTCAAGGTGTAGTAAAAGGTTCTGATTAAATTTGATAAAATTGCTTTCATATTGGGATAGTATTAACTTTTGGTTGTCCAAAGTATTTAACTGATTTTTcAATTGGTAATTCTCCAAGTCTAATTTGTCTAACTGATTTTGTATTACGTGGAATTCCTCTGATTTATCGATTTGAAGGTCATTGATTTGTTTTTCCAAGTCATTGATGTAGCTGTCCCAATTATTTTCCTTTAGTTTATTGATTTTGGTCTGAGTTTCCAGTTCTTTGGTTAAAACTTTcTCATTTTGTTTCATTTTAATGATGTCTTCCTTCAATTTTTCCAAATTGTTTATCAAGACAGATTGCGATTCGATCTTTTCCTTCAGTTGGGAAATCAAATGATCcTGTTTTTCTATAACGGAACTAGAATTTTCTTCCAATTCTAAGGAATCTAGTAGATGAGACTGCTCATTTAGTTTGGACGCTATTATTTTTTCTAATTTTTGCGATTTCTCGAATTTCAATCTAATGGAATTTATAAATTGATCATATTCTTTGTGCAAATTTTCTATGACAATCTCTAATTGAGTGTCTAAGGTTTTCTCAAAACGAGACTCGgCGGCTGAAATTGGTAAGGAAGTACTGTTTGCCATTACATTTTCTGTATCAcTGTGGTTGTTACTGTCATGAATTTCGTCATTCTGGTATCCACCATCAGcATTCTCCTCTTTACTTGATGGcGAGTCCCTACTTTCgAACTGTGATCCTGCTGGTGAGGACTGGGCCAGTGAAAGAAATTCTTCCTTgTCCTGCTTTGATTCCGCTCGACTTTTGGAATGCAAAAAGTCCTGCAAGAATTGGATGATGAACTTGGACAAAGTATCCATTTTTTCAAGAACATAATCCGAGCTCAGCTCTAAAGTTTCCTCCAGGGTCTCCCTCTCTTCTTTATCAAGAAGATGaGCATTTTCATCTTGCTCATTAAAATGCGTCAATATAAAGGACAATAGGTGATTGATCGTATTCACGATACTTTCCGAATTTTCTAAATTCTCTTGAACGAATTGTAGCGTATCTTGGTACTCGACTTCCTTCGCCTTTAAATCCTGTTTCAATTTGTTTATTTCAAGATTTAGACCCTCGATAATCGAATTTCTAAAATCAGTGTCATTGCCCAGCGATGGTGCATTGCCGTCTTTATTAGGGATTCTGCGAATGTATTCATAGAGTACTTGAATCTTGATTTTGGCATTAGTCAACTCCTTCTCCAAATTTTTGACTTTGTTGGAATCGTTCATCAGAGCAGGCTTGATGGGATCGTTATGAGATGACCTCGTGGTCATGGAGTCCCTGAGTGATGGTATGGACATCCCAGAATCCATCGAGTTTGTGAACTCaCTGTCGTCATCATCACCAGTGTTGTCATTATTGCGAAGATGCCTGCCACTAGGAATCCATCGACGTACCATGGCTATAACTTTCtTTATGTcGTTTGCTTAGTTTTTTGATATTAGTcTTGCTTATGTGAAATTTCGCGATTTCAATTAAAATAATAAATACATATATAAAGAATATACACAGAGGGAAGCAAAAGT**

*AIM2* MAR 1and *kanMX* amplification primer 2:

CCAAACTCCTACCTATTCATTTCATCAATTAATTAATATTATATAGCCACGAATTTATGACCTTGACAGTCTTGACGTGCGCAGCTCAGGGGCATGATGTGACTGTCGCCCGTACATTTAGCCCATACATCCCCATGTATAATCATTTGCATCCATACATTTTGATGGCCGCACGGCGCGAAGCAAAAATTACGGCTCCTCGCTGCAGACCTGCGAGCAGGGAAACGCTCCCCTCACAGACGCGTTGAATTGTCCCCACGCCGCGCCCCTGTAGAGAAATATAAAAGGTTAGGATTTGCCACTGAGGTTCTTCTTTCATATACTTCCTTTTAAAATCTTGCTAGGATACAGTTCTCACATCACATCCGAACATAAACAACCATGGGTAAGGAAAAGACTCACGTTTCGAGGCCGCGATTAAATTCCAACATGGATGCTGATTTATATGGGTATAAATGGGCTCGCGATAATGTCGGGCAATCAGGTGCGACAATCTATCGATTGTATGGGAAGCCCGATGCGCCAGAGTTGTTTCTGAAACATGGCAAAGGTAGCGTTGCCAATGATGTTACAGATGAGATGGTCAGACTAAACTGGCTGACGGAATTTATGCCTCTTCCGACCATCAAGCATTTTATCCGTACTCCTGATGATGCATGGTTACTCACCACTGCGATCCCCGGCAAAACAGCATTCCAGGTATTAGAAGAATATCCTGATTCAGGTGAAAATATTGTTGATGCGCTGGCAGTGTTCCTGCGCCGGTTGCATTCGATTCCTGTTTGTAATTGTCCTTTTAACAGCGATCGCGTATTTCGTCTCGCTCAGGCGCAATCACGAATGAATAACGGTTTGGTTGATGCGAGTGATTTTGATGACGAGCGTAATGGCTGGCCTGTTGAACAAGTCTGGAAAGAAATGCATAAGCTTTTGCCATTCTCACCGGATTCAGTCGTCACTCATGGTGATTTCTCACTTGATAACCTTATTTTTGACGAGGGGAAATTAATAGGTTGTATTGATGTTGGACGAGTCGGAATCGCAGACCGATACCAGGATCTTGCCATCCTATGGAACTGCCTCGGTGAGTTTTCTCCTTCATTACAGAAACGGCTTTTTCAAAAATATGGTATTGATAATCCTGATATGAATAAATTGCAGTTTCATTTGATGCTCGATGAGTTTTTCTAATCAGTACTGACAATAAAAAGATTCTTGTTTTCAAGAACTTGTCATTTGTATAGTTTTTTTATATTGTAGTTGTTCTATTTTAATCAAATGTTAGCGTGATTTATATTTTTTTTCGCCTCGACATCATCTGCCCAGATGCGAAGTTAAGTGCG

*AIM2* MAR 3 and *AIM2* MAR 4:

AGCGTGATTTATATTTTTTTTCGCCTCGACATCATCTGCCCAGATGCGAAGTTAAGTGCGAACTGACCGATAATATAAAGTGCTCAATATATATATATATGTATATAACGGTTAACGTAAGAAGAGCTCTTCCCTCTTAAACATTCGAAAAATGATTGAACCAGTATATTTGGTCGAGCAAGACTTTCTCCTTCGCATATTTTACGGCAGGTATGGATATATCGCCTCTTGCgGCAAACCCGTGAGCCACACCACTGAAGAGGTCTAACTGGTAAGTAGCGTGATTATCCTTTAATTTTTCCTCCGTTAAGTGTCTTAAGTTTGCCGGAAAGATGTGATCCTCTTCCGCTGCTGAAATCAATATTGGTTTCTTGCTATCAATTGCTTCAATTTCCTCGATGCTGACGAAAGATGGATGTGCAATGGCTGCAGCATTGGCAAGACCtCCGTCGCCACTAATGTGTTGGACGGCAAACTTTGCACCAAAACAGTAACCCACAACGCCAATAAACTTTGGGTCATATTCAAGTTTTAACAACTTCATGAATCCATCAACAATTTTCTTGGTGACTTCAGGAGAATGTCTTTGAAACCAGGCATCACGATCAATTGGTTTGTCCGATGAGATAGCATCGCCGAATAAAATATCGGGAACAAAGACCATGTACCCAGCACTAGCAAATTTGTCGGCCGTTAATAAAACATTGTTGAATTTATTGCCATACACATCTGTCAAGATAACTATAACTTTTTCCTTGGGAGATGTAGAGCCTGCTGCATAAGTATCTAAACCGAAGATTTCTTCACGACGACCCTTGGGTGTTCCATCGTGACAAACTCCTTCAAAGCAACACTTGCCAGGTTGATTAGATGCCATTTGATTGtAATAATTTTaTTCTGCTGTAGTTAGACGcAGTGGAAAACTTTAAGTCTACTGAGTCTTGAGACCTTATCACCCTTTGAAGGTTTCTTGCAAATGAGCGTGGTTTGGCATTTTTTATCGGAAAGAAAAAAgGGGCTCCGCCTTAGGCCAGATATCATAGAAATGCAACACTTCCCTA

*ACS1* MAR 1and *kanMX* amplification primer 2:

TGTAAAGAATAAATAATTATAAGTATAAATAAAAAGAGAAGGTGAAATAATAATAAGTAACCTTGACAGTCTTGACGTGCGCAGCTCAGGGGCATGATGTGACTGTCGCCCGTACATTTAGCCCATACATCCCCATGTATAATCATTTGCATCCATACATTTTGATGGCCGCACGGCGCGAAGCAAAAATTACGGCTCCTCGCTGCAGACCTGCGAGCAGGGAAACGCTCCCCTCACAGACGCGTTGAATTGTCCCCACGCCGCGCCCCTGTAGAGAAATATAAAAGGTTAGGATTTGCCACTGAGGTTCTTCTTTCATATACTTCCTTTTAAAATCTTGCTAGGATACAGTTCTCACATCACATCCGAACATAAACAACCATGGGTAAGGAAAAGACTCACGTTTCGAGGCCGCGATTAAATTCCAACATGGATGCTGATTTATATGGGTATAAATGGGCTCGCGATAATGTCGGGCAATCAGGTGCGACAATCTATCGATTGTATGGGAAGCCCGATGCGCCAGAGTTGTTTCTGAAACATGGCAAAGGTAGCGTTGCCAATGATGTTACAGATGAGATGGTCAGACTAAACTGGCTGACGGAATTTATGCCTCTTCCGACCATCAAGCATTTTATCCGTACTCCTGATGATGCATGGTTACTCACCACTGCGATCCCCGGCAAAACAGCATTCCAGGTATTAGAAGAATATCCTGATTCAGGTGAAAATATTGTTGATGCGCTGGCAGTGTTCCTGCGCCGGTTGCATTCGATTCCTGTTTGTAATTGTCCTTTTAACAGCGATCGCGTATTTCGTCTCGCTCAGGCGCAATCACGAATGAATAACGGTTTGGTTGATGCGAGTGATTTTGATGACGAGCGTAATGGCTGGCCTGTTGAACAAGTCTGGAAAGAAATGCATAAGCTTTTGCCATTCTCACCGGATTCAGTCGTCACTCATGGTGATTTCTCACTTGATAACCTTATTTTTGACGAGGGGAAATTAATAGGTTGTATTGATGTTGGACGAGTCGGAATCGCAGACCGATACCAGGATCTTGCCATCCTATGGAACTGCCTCGGTGAGTTTTCTCCTTCATTACAGAAACGGCTTTTTCAAAAATATGGTATTGATAATCCTGATATGAATAAATTGCAGTTTCATTTGATGCTCGATGAGTTTTTCTAATCAGTACTGACAATAAAAAGATTCTTGTTTTCAAGAACTTGTCATTTGTATAGTTTTTTTATATTGTAGTTGTTCTATTTTAATCAAATGTTAGCGTGATTTATATTTTTTTTCGCCTCGACATCATCTGCCCAGATGCGAAGTTAAGTGCG

*ACS1* MAR 3 and *ACS1* MAR 4:

AGCGTGATTTATATTTTTTTTCGCCTCGACATCATCTGCCCAGATGCGAAGTTAAGTGCGGCAGCTCGGTTATAAGAGAACAAAAACACACGAAAAAAAAAAAGTCGTCAATATAAAAAGGAAAGAAATCATCATTACAACTTGACCGAATCAATTAGATGTCTAACAATGCCAGGGTTTGACAATGTAGAAACGTCGCCTAGTTGGTCACTTTCTCCTGCTAGGATTTTTCTTAAAATACGTCTCATAATTTTGCCaGATCTTGTCTTGGGCAAGTCATCCACTAAAATGATCAATTTTGGTGCGGCAAATGGCCCGATGTCTTTTCTAACAGTAAAGACCAAATGCTTCTTGATATCTTGTAATTCATCATCTGTTGCGGTGGACCAACTAGATTTGTTTTTCAACACCACAAATGCAGCAACTGCTTGACCAGTCAAGTCATCGTTGAATCCGACAACAGCACACTCGGCCACAATTGGATCTTCGATAATAGCAGCCTCAATTTCAGCGGTAGACAGACGGTGACCAGAGACGTTCACCACATCGTCTACACGACCCAAAATCCAGATATAACCATCCTTATCCTTTGCAGCACCATCACCAGTGAAATAGTAGCCAGGGTAAGGGTTCAAATAAGTGTCTAGATACCTATCATGATTTTTCCAAATAGTTCTTGCAAATGATGGCCATGCAGCTTTGACGGCAAGGACACCCTCTGCGTGGCTGGTaTTAAGTTCTTCACCAGTGTTAGGGTCAAGAACAACTGCATCAATACCGAAGAAtGGGAATGAGGCAGAACCCGGTTTCATTGGTGTgACACCACCAGCCAGCGGGGTGACCAGATGCGAACCAGATTCTGTTTGCCAGTAGGTGTCTACAATGGGGATTTCATTTTTACCTATTTTTTCAGAGTACCACTCCCAAACTTCAGCAGCAATTGGCTCACCGACCGAACCCAAGCAACGCAAAGATTTTAAGGAATGATTTTCGATGTAGGAATCACCAGCTCTTTTCAACAAACGCAAAGCAGTTGGgGCAACATAAAATTGGGTGACTTTGTGTTCATCAATAATATCCCAATAACGGGAGTAATTTGGGTACGCAGGAGTCCCTTCAAAGACCAAAGTGGCACAACCATATAGTAAGGGACCATAAACCACATAAGTGTGGCCTGTAATCCAGCCAATGTCTCCAGCTGTGAAGAAAACGTCTTCTTGGTGAGTGTCAAAAGTGTAGCGCATGGTCAACAAAGCTCCCAGCAAGTAACCTGCGGTAGAATGTTGAACACCCTTGGGGGCACCAGTAGAACCAGACGTATACAACAAGAATAATGGATCCTCAGAATCAACGGGTGTGCATGGATAGTAGGTCTTGTATTTCTTCTTTTCTGTTGCCCAATCCAAATCTCTGGGGGCATGGAAAGCAACAGATGGATTGTTGGTCTTTCTATAAACCAAGACGTGTCTCACGCCTGGGGTCTCTCTTAGCGCGTCATCAACAATTCTTTTAGTCTCAATGACTTTACCACCTCTGTTGGATTCATCTGTAGTGATGACAACTTTAGAGTCCCCATCGTTGATACGATCTCTCAAGGAGTTGGAAGAAAACCCGGCAAAGACTACGGAGTGAATGGCgCCGATACGGGAAATGGCCAACAAGGTTATGATTGCTTCTGGGACCATAGGCATGTACACGGCAACAGTATCGCCCTTGCGAACGCCCATAGAGTAAGTCAGCACTTGTGCCACTTGACAAACTTCTTCAAGTAGTTCCTTGTAGGTAATGGAATAGCCTTGGCCAGGCTCGTCACCTTCGAAAATAATGGCTTTCTTGTTAGGgGTCTTCAAGGCATGTCTGTCAACACAGTTGTAACAGGCGTTTAATTGGCCGTTGAGGAACCATGCATTGTTCTGGAAGGAGGGCCTGCCCGTTTTAGGGTCTGGGATGAACACCTTATCGAATGGCTTAGACCAGTTTAAAAATTGGGTAGCTTTAGAACCGAAGAACTTAGCAGGGTCTTCAATAGACTCCTTGTGCAAGCGCTGATAGTCCTGCAACCCGTCCAAGTGTGGAGAATAGTGGGTAGCAATTGCGGGCTGCAGTCTATCTGAGATGGGCCGTTGTGGCACGATCTTGACCGAgGTCAAATGTTCATACTCATGTTCCTTCTTCTGCTGCGCAGTGGaGGCAGACTGGGACATTTTTGCTTTCAACTTGTCAATTTCACTTGACTGTTCTTCTAGTTTTGATGATTGTACGGCAGAGGGCGACATAaCACAGTGGGCAATGTCTTTCTAGTAGTTTTGATATGTTTGGTTTTGCTTATAGATAGAAAATATAAGAACAAGATATAACGTACTACCAGATAACCTAAGGGAGAAATATGCTTAGAATAGCCGCCCAGTTTATATACAAAATGAAGGGAGAACTATTTGCCACCGAGGAACTaTACCCCAACTGCAATACCCATTGAATAATGGCATCGGAGGCTCGGCGGCAATTCGTACCCCAACCTTTTTTTTTTACTTTTCTTTGaATCTTAGAGATAACAGAAAAAAAGGATGACCCCAATCATTTGCCACGGCATGTCAACAGGTGAGTGCCTTTTGAGGGGGGGGGGTCATCTCGACATCCGGCGAAATGGAGCAGTCACACGTGAACATTTTTAGGGGATGGAGAGTGCTACGCCGTTCGTCCGAGATGATTATCATATTTACACAGCCGTACATACACGTGCCATTTATCTTGATATCATTCTGGACGTATGTGCACATGTGATTTGCTTTTGTTTTTTTAcGAATGTCGGGTAATAAACAGATTGTTTTTCTGGGAGGATAATCTTTTCTTTTTTCCTGTTGGTATTCTAAAATTAACCTTGCTGTTTCTTTTTTTTTTTTTTTTCGCGCGACTACTCAGCCATCTTGCATTTTTAAAGAAAAAGATAATCATgAATGCCTTCACGGGAATACGTATAGAACATTATTAAAAGTATATGAATGGCATATATATATAGAACACCACCCTTGGAAAACATTTATACCCCTTAAACTAAAACAATTTGCTGCGCTATACCGTGTTTCAcTGTATTATAATACATTCATTTCTGTTTCATTACGATTATATTGACGTG

*OAF1* MAR 1and *OAF1* MAR 2:

TGTACGCAATTGCCGCTTTTTTCTGACATCTTTTTTGACGTGTAGAGAAGGAAACAGATCCTCCAGAAGGGATTTACTGTTGGCTATTTTGTGcTAGAAGCAGGTTAATAATAGATTAGGTTGCGTAAGTCATGGTCGAAAATAGTACGCAGAAGGCCCCACATGCCGGAAATGATGATAATAGCTCTACCAAGCCATATTCGGAGGCGTTTTTCTTAGGGTTCAATAACCCAACGCCTGGgTTAGAAGCTGAGCACTCAAGCACATCGCCTGCCCCCGAGAACTCCGAAACACATAATAGGAAAAGAAATAGAATATcGTTcGTCTGCCAGGtTTGTAGGAAGTCAAAAACAAAGTGTGATAGAGAAAAACCTGAATGTGGTCGATGCGTCAAGCATGGGTTAAAATGTGTTTATGACGTATCAAAACAGCCAGCACCACGAATTCCGAGTAAAGAtGCCATTATATCAAGGTTaGAAAAAGATATGTTTTATTGGAAAGATAAAGCTATGAAGCTACTAACAGAGAGAGAGGTGAATGAATCAGGCAAGAGATCAGCAAGTCCGATCAATACAAACAATGCTAGCGGGGACAGTCCTGATACCAAGAAGCAGCATAAAATGGAACCTATATATGAACAAAGTGGTAACGGGGATATAAACAATGGTACtAGAAATGATATTGAAATCAACTTGTATAGAAGTCATCCAACCATGATCATGAGTAAAGTCATGAAAAGAGAAGTTAAGCCGTTATCTGAAAATTATATTATAATTCAGGACTGTTTTCTAAAAATCCTGGTCACTTCAGTGTTCCTTGACACTTCAAAGAACACGATGATACCGGCATTGACGGCAAACGCGAATATTACAAGAGCCCAGCCTAGCGTAGCAAATAACCTTTTGAAATTGAAGGAAATGCTAATCAGACAGTGTCAAACCGAAGATGAAAAAAATCGTGTAAACGAATTtACTGATAGAATACTACAAAATACAAATTCAAATAGAAACTTGAAAATCGGTATGCTATTATCAATGCTTTACAATTCTGTCGGTTACCAATATCTGGAGGATCATTGCCCTCAAGGTGGCGAATATTCGGATTTATTGAGAAATTTGATCAATGAATGTGAAGCTATTTTGCCATCTTACGAAATcATTGAACGCTACAAGAACCACTTTTATGAGTACGTTTATCCAAGTCTACCTTTCATCGAATTAGAAATTTTTGAAGAATCATTAAGTCAAACAATTTTTCCGGACCCAAACAACCCCTCCAAGGTGCAAATACGTATGGGTAGCACACATTTGAGAGCTAAGGTGGAAAACTTGAGTCTTCTATTGGTTATCTTGAAACTCTCATACATGTCAATAAGGcTTTTAGATCATAGTACAGCAGACTCGAGTTTTTATCTTTCAAAGGAAATAATTGATAAATAtCCAATACCGAACGATTTTATTTTATTGAGTCAAAGATGTCTAGCATCGGAAAATTGGTGTGCATGCGCTAATGAAAACATCATATCATGTTTACTATATATCTGGTCTTTTTTTGCTTTTTCTCCTGAAGAGGGTGATTTCTTTCTCGAGCACCCCACCGATGTTATCAGTAGTTTGATAATGATGCTTTCCACCTCGATTGGTCTCCACAGAGATCCTTCAGATTTCCCTCAATTGATTTCCCCGTCCACCTCAGATAAAAGAACCTTGAATCACAGAAGAATACTCTGGTTGAGTATCGTTACCGTTTGTTCGTTTGAAGCAAGTCTCAAgGGTAGACATTCTGTCTCACCGATATCTTTAATGGCCTTATTCCTAAATATTAAGGATCCTGATTCcCTGACGGTATATATGAACCGAGTTAGGGGCGATCTAAGCGATATCAATAATCACAAgCTTTTGAGAATTCATgAATTTACATTtAAGAGAGCCCAGCTTGCGTTACTCCTGTCaGACTTAGATAACTTGACGATGACATAtTATGGTAGTTTCCATTTGCATTCAATTGAATTCATAAGAGAAAAaATTGAGATTTTTGTGGAGGAAAACTTTCCCATAGTACCATTgAAAAGTGTCGCACAGGATAAGTCAGACCTTGATGACATGAATGTGATTTCAGAAATGAATATATTATCTTCAGAAAATTCTTCTTCATTTCACAATCGAATAATGAATAAACTATTGATGTTGAGAACTTCAATGGCCGTATTCTTGCATTTTGAAACACTTATCACTAAGGATAAAAGTATCTTCCCATTCTACAAaAAATACTTTATGGTTAGCTGTATGGATGCGTTGTCACTAATAAATTATTTCAATAAGTTTTTCAACGGAGAATATCGACACGCAATATCTTCTTTAACCAGTTTTAATGTTACAAAATTTATTCAGTTAGCACTATCCAGCACAATCTTCAGCCTATTAGGGATTATACTAAGAATAGGTTTAGCCATCCATATGTTATCTTCTGAAGTACAAAAGTTATCGGGAACGACAGATCCAAGAATAAAGGAGTTAAATACCAAAGTtGAAAAATTTAGTACCCTGCAAAGAGATCTCGAGTCTGCTTTAGAAGGTATATATTGCTCTGCTTCGGAACATTTAAGATTCACATACTTCCCCGTTTTTAAGATGTTGGCTTTATTCGATGTCATTGTACAAAGaATGAGAAAGGGTGAATTATGGCACGGCATATTTACGATGATTCAAATGGAACAAATGCATTCTAGGATAATCAAGACATTAAGCATTACCTTAGGAGTCAAACTGGACAAAAAGGATAGGCTATTAGAGGAATTGATGGCATGCAATCACGTTGCGAATTTTAGCGTTGAAGATATAGATGAGCTGAACCGcAATATtAAAAAAGAGATTCAAATTTCTTCAGGAcTGAAGCCGCCTGTAAACACAATTGACTTgACCAACGGCGAACCAaTCGGcAATGCTGTTCCTACCTTCACAAAGACATGGAGTTCATCCTTAGATAATTTAGAAAAACTATCgTCGGCCGCTGCAGTTGGTCAGAGCTTGGACTACAACAGTGGTTTACGTCAGGGaCCTTTGGCGGGTGGTGGTTCAAAAGAGCAAACGCCAATAGCCGGGATGAATAACTTGAACAATTCAATCAATGCTACACCAATTGTCGATAACTCATCTGGATCACAACTTCCTAATGGTTTCGATAGAGGCCAAGCGAATAATACTCCTTTTCCAGGTTATTTTGGAGGTTTGGATTTATTTGATTATGACTTTTTGTTTGGCAATGACTTTGCTTAAAAATTTTCTTTCCAAACTCCTACCTATTCATTTCATCAATTAATTAATATTATATAGCCACGAATTTATGAAACTGACCGACCTTGACAGTCTTGACGTGCGCAGCTCAGGGGCATGATGTGACTGTCGCCCGTACATTTA

*KanMX* amplification primer 3 and *OAF1* MAR 4:

CCTTGACAGTCTTGACGTGCGCAGCTCAGGGGCATGATGTGACTGTCGCCCGTACATTTAGCCCATACATCCCCATGTATAATCATTTGCATCCATACATTTTGATGGCCGCACGGCGCGAAGCAAAAATTACGGCTCCTCGCTGCAGACCTGCGAGCAGGGAAACGCTCCCCTCACAGACGCGTTGAATTGTCCCCACGCCGCGCCCCTGTAGAGAAATATAAAAGGTTAGGATTTGCCACTGAGGTTCTTCTTTCATATACTTCCTTTTAAAATCTTGCTAGGATACAGTTCTCACATCACATCCGAACATAAACAACCATGGGTAAGGAAAAGACTCACGTTTCGAGGCCGCGATTAAATTCCAACATGGATGCTGATTTATATGGGTATAAATGGGCTCGCGATAATGTCGGGCAATCAGGTGCGACAATCTATCGATTGTATGGGAAGCCCGATGCGCCAGAGTTGTTTCTGAAACATGGCAAAGGTAGCGTTGCCAATGATGTTACAGATGAGATGGTCAGACTAAACTGGCTGACGGAATTTATGCCTCTTCCGACCATCAAGCATTTTATCCGTACTCCTGATGATGCATGGTTACTCACCACTGCGATCCCCGGCAAAACAGCATTCCAGGTATTAGAAGAATATCCTGATTCAGGTGAAAATATTGTTGATGCGCTGGCAGTGTTCCTGCGCCGGTTGCATTCGATTCCTGTTTGTAATTGTCCTTTTAACAGCGATCGCGTATTTCGTCTCGCTCAGGCGCAATCACGAATGAATAACGGTTTGGTTGATGCGAGTGATTTTGATGACGAGCGTAATGGCTGGCCTGTTGAACAAGTCTGGAAAGAAATGCATAAGCTTTTGCCATTCTCACCGGATTCAGTCGTCACTCATGGTGATTTCTCACTTGATAACCTTATTTTTGACGAGGGGAAATTAATAGGTTGTATTGATGTTGGACGAGTCGGAATCGCAGACCGATACCAGGATCTTGCCATCCTATGGAACTGCCTCGGTGAGTTTTCTCCTTCATTACAGAAACGGCTTTTTCAAAAATATGGTATTGATAATCCTGATATGAATAAATTGCAGTTTCATTTGATGCTCGATGAGTTTTTCTAATCAGTACTGACAATAAAAAGATTCTTGTTTTCAAGAACTTGTCATTTGTATAGTTTTTTTATATTGTAGTTGTTCTATTTTAATCAAATGTTAGCGTGATTTATATTTTTTTTCGCCTCGACATCATCTGCCCAGATGCGAAGTTAAGTGCGTAATATAAAGTGCTCAATATATATATATATGTATATAACGGTTAACGTAAGAAGAGCTCT

*GPB2* MAR 1and *GPB2* MAR 2:

CCACAACGGCTTCTCTTTTATAGATGGTTAACAcTATAGTATCAATATTATCATCATGATTAAATGATGATGTATAATACTTACCCGATGTTgAATCTTATTTTTTCATGtAGTAAGTAATCATGCAACAAGAAAAACCCGTAATTAAGgGAACATAGAACAACTAGCATCCCCGATAAGACGGAATAGAATAGTAAAGATTGTGATTCATTGGCAGGTCCATTGTCGCATTACTAAATCATAGGCATGGAAATTTCCAGTTCACCATGGAACGACGGTGGATACAGCCCCTATGAGAGAAACAGAGTCGCTGTATCACCATTTTCATCAGCGTTGGAAGGCGAAGAACGAATAGAAACCTCTCGATCTTTGGGTaATCATTGCTTTGAACCTTTGCCATACGTGACGAATTATCTTTCTATTTTCGCGCTTTTTGGTAAAGAGATATTTGGTGACAAGGGAAATGTGAGCTCAAGAAATGAATATTTGCTAAAAAAATACTACTCTTTGAAAAAGCCATTTGTATTGCGACATAATGGGCATGCGTTGAAGAATCCCGACATGCCACTCCAGAGGAATGACATATTGCAAACCAATTTCATGGTTGACAAATTTCTGAATCGTACTGTGCGGTCAGTGAATTTTAATAATTTCAAGATAATATCAGATATGCAAAGTAAAAGCGGTCGAGGAACAAAGTCAGGCACAAATCAGAATCAAAGTGCCGACGCTATTCAAAATATTTGTCTACCATCTATACCGTCGGCGTTGCCTTATTTCCAaTATTATAGGAAGCTATTGACAGTTAATACCAAAGAATGGGATATTTTAAAACTGCACAGTTTATGGGTACCAAAGCTAAGGAAGGATTTTAAAGATTTTTCGTTGcATGGTGATAAAAACTCTccAAAGCCGATCGATAGTCACTATGATGAGGATAATACCATGAAaAAAAATTTATTTTTTGAAAGATCTCCAAGTCGACAGACTCTAGATGGTAAAGGGTGTGCCTCTAAGGGGTATGACATTTCTTCCGGTAATATGATTATCCtATCCCTATTTTCTGAAGATAAGCTGCCGGCTTTAACTTATCATTGTTCCGTAGAATTAAATGGAAACATTTACATATTTGGGGGATTGATGCCATGCTACAGCTATGAGGAGGATGCGCCGATGCTGAACGATTTTTTTGTAGACGGAATAAAGAACTTACCTCCGCCTTTAtTACCTCAAGTGATTAATAATCCATCAATGGTCAATAATCCTCATCTTTATGTCGCTTCTATACCATCATGCCGGTTTAGCAAACCTAAAATGGGGGGTTATATACCGCCTCCATTGCTATGTGTTCAAGGATCCAAATTAACaGACCGACATATTTTCTTTTATGGCGGATTTGAAATCAGGACAGAAACCCGTGGTGATGAAAATGGGAAGTATCATCTCAAGAAAAGATTATATGTGAATAACACTGGTTACATACTCGATATTATGTCGTTCAAGTTCACTAAAATAGATATCATAGTACAACCTTCCAAATATAATGCATATCCGACAATGTCATCGAGGTTTGGTCACTTACAAATTTCTATTGATAATCCAAATAGGAGAGCTAGCGTTCATTCTTCAAGCATGAACGAAATTCATAAAATGGGGAGTGCTTCCATGAAACAAGGTAGCAGCATCACTTCCGGGCGGCTTGAAAAAGCAGCAGTACTTTCATCATTACCTCATAATACTGTGCACACGGTTATAATATTTGGTGGTTACAGACAAACCGGTGATGATCGTTACGAAGCAATGAATGATTTGTGGAAGATAGAGATACCCGTGATACGTCGCGGTAAgAAAGGcTATTGTAAGTTTTCAGAGACAGCTAACGCGATACTACTGACGCCAAGgGAAAAGGACAAATCGGATTGGCCCGAAGAAAGAGCCTTTTCTGCCTTTTCTGTTCATGGGACTTCGTTAATGGATAGGAGTTCTCTTGACATGAGACTATTGAACAACTTAAAAAACCATTTTGTTTTAAAACCGTCATATATATCACAGGATCGCGTTGTTAGTCCTAAACCGGTTTTCCCCATGATGGTTCATGGCACGCATCAAGATCTTTTCAATAGTGGCTCTGCGGCACAAGAATCGCCCAAAGCTGGTGCCTCGGCCAGCAGCGCAAGTGCTGCGAGCTTTGATCCCGATATGGACGATAATTTGGAAAATTATATAGTCAATCCAGGGAGAAAATCGTCATCTATTCCAATGACTaCGATAGGGAGACAGAGATTAATTTTAAGCCAAGAGAAGCCAGTAGGTAAAACTGTTGTATTGCATGGTGGGTCTAACGGTCTCAACGTTCTTGATGATATGTGGTTGATGGACTTAGAGTGTGAGACATGGACTCCAATAGAGACATTTGCAAAGGCAGATTCGAGCGAAGACGGTGATGAAAAATTGGATAGTGTGAACGTgGGTCTCGTTGGCCACAGgATGGAAAGTATTGGACGAATATGTGTATGTATAGGTGGTATGGTACAAGAGGATGTTGACCAATTTTACTCGGAGAATGATGATGAGtCTCCTCGAAAACGCAAGGTCGATACATTACCGTTGGGTGGTAATTTTTTGAACACAATTGATTTAAGCACGCAGTGTTGGGAAGAACATAAAATTACTCTGTCCAAGAAGGAAGACGATGAGGACAGACAAGATAGCGAAAATGAAGAcACgAATTCAAATATAGTAGTTGGTGTCGGTGGCACTTCTTTGCAATGTGACAAAAGTATTATTTTaATTGGCGGATTGATATCTAGACGGAGCAATGTAAAAGAAATATATTTACATGGTACCATAACGAAAAGTATTTTTCCTAGtGTAAATCCTAGTGCATAAAAAGGCAGTTTTCAATGCTTTCACTTTGTAAACTTTGTTTAGTAGTAGAATATAATATATTCAGTTTTGTTTTATAGTCACATAAtACTTTGTCTTTCAAAGAATAATCTCCTTCGCAATACCAGCGAAATATTTTGGCACCTTGACAGTCTTGACGTGCGCAGCTCAGGGGCATGATGTGACTGTCGCCCGTACATTTA

*KanMX* amplification primer 3 and *GPB2* MAR 4:

CCTTGACAGTCTTGACGTGCGCAGCTCAGGGGCATGATGTGACTGTCGCCCGTACATTTAGCCCATACATCCCCATGTATAATCATTTGCATCCATACATTTTGATGGCCGCACGGCGCGAAGCAAAAATTACGGCTCCTCGCTGCAGACCTGCGAGCAGGGAAACGCTCCCCTCACAGACGCGTTGAATTGTCCCCACGCCGCGCCCCTGTAGAGAAATATAAAAGGTTAGGATTTGCCACTGAGGTTCTTCTTTCATATACTTCCTTTTAAAATCTTGCTAGGATACAGTTCTCACATCACATCCGAACATAAACAACCATGGGTAAGGAAAAGACTCACGTTTCGAGGCCGCGATTAAATTCCAACATGGATGCTGATTTATATGGGTATAAATGGGCTCGCGATAATGTCGGGCAATCAGGTGCGACAATCTATCGATTGTATGGGAAGCCCGATGCGCCAGAGTTGTTTCTGAAACATGGCAAAGGTAGCGTTGCCAATGATGTTACAGATGAGATGGTCAGACTAAACTGGCTGACGGAATTTATGCCTCTTCCGACCATCAAGCATTTTATCCGTACTCCTGATGATGCATGGTTACTCACCACTGCGATCCCCGGCAAAACAGCATTCCAGGTATTAGAAGAATATCCTGATTCAGGTGAAAATATTGTTGATGCGCTGGCAGTGTTCCTGCGCCGGTTGCATTCGATTCCTGTTTGTAATTGTCCTTTTAACAGCGATCGCGTATTTCGTCTCGCTCAGGCGCAATCACGAATGAATAACGGTTTGGTTGATGCGAGTGATTTTGATGACGAGCGTAATGGCTGGCCTGTTGAACAAGTCTGGAAAGAAATGCATAAGCTTTTGCCATTCTCACCGGATTCAGTCGTCACTCATGGTGATTTCTCACTTGATAACCTTATTTTTGACGAGGGGAAATTAATAGGTTGTATTGATGTTGGACGAGTCGGAATCGCAGACCGATACCAGGATCTTGCCATCCTATGGAACTGCCTCGGTGAGTTTTCTCCTTCATTACAGAAACGGCTTTTTCAAAAATATGGTATTGATAATCCTGATATGAATAAATTGCAGTTTCATTTGATGCTCGATGAGTTTTTCTAATCAGTACTGACAATAAAAAGATTCTTGTTTTCAAGAACTTGTCATTTGTATAGTTTTTTTATATTGTAGTTGTTCTATTTTAATCAAATGTTAGCGTGATTTATATTTTTTTTCGCCTCGACATCATCTGCCCAGATGCGAAGTTAAGTGCG

AAAAATTAACAATTAGGTTCATAGTCCCCTAATTCAATTAATCGAAAAAAAAAAAATAAA

*PEX22* MAR 1and *PEX22* MAR 2:

CTTTCACTTTGTAAACTTTGTTTAGTAGTAGAATATAATATATTCAGTTTTGTTTTATAGTCACATAAtACTTTGTCTTTCAAAGAATAATCTCCTTCGCAATACCAGCGAAATATTTTGGCAAAAAATTAACAATTAGGTTCATAGTCCCCTAATTCAATTAATCGAAAAAAAAAAAATAAAATATAAGGGAAGATTGTGCTGATGAAATAGACAATGAAACAATAATGAAGAATAAAGAAGAAGAAGATATAAAACATGCCACCACCATCAAGAAGTAGAATAAACAAAACAAGAACATTAGGAATAGTGGGTACAGCTATAGCAGTGTTGGTCACaTCCTACTATATATATCAAAAGGTGACAAGTGCAAAGGAAGATAATGGGGCACGACCTCCAGAGGGTGATTCAGTAAAAGAGAACAAAAAGGCAAGGAAGAGCAAATGTATTATAATGAGCAAGTCGATACAAGGACTGCCCATAAAGTGGGAGGAGTACGCCGCTGATGAAGTGGTTTTGCTGGTACCTACGAGCCACACTGATGGATCAATGAAACAAGCCATTGaGGATGCCTTTCGCAAGACGAAAAACGAACACAAAATCATATAcTGCGATAGCATGGATGGATTATGGTCtTGTGTAAaACGGCTAGGTAAATTTCAGTGCATATTaAACTCCAGGGACTTCACAAGTAGTGGTGGTAGCGATGCgGCAGTtGTTCCTGAAGATATAGGCAGGTTTGTgAAATTTGTTGTTGATAGCGATaTAGAGGATGTGCTGATTGACACTTTATGCAATTAATGTAGAAAAGAGTTTCTTGTAACAGTATGTAAAGAATAAATAATTATAAGTATAAATAAAAAGAGAAGGTGAAATAATAATAAGTAAGCAGCTCGGTCCTTGACAGTCTTGACGTGCGCAGCTCAGGGGCATGATGTGACTGTCGCCCGTACATTTA

*KanMX* amplification primer 3 and *PEX22* MAR 4:

CCTTGACAGTCTTGACGTGCGCAGCTCAGGGGCATGATGTGACTGTCGCCCGTACATTTAGCCCATACATCCCCATGTATAATCATTTGCATCCATACATTTTGATGGCCGCACGGCGCGAAGCAAAAATTACGGCTCCTCGCTGCAGACCTGCGAGCAGGGAAACGCTCCCCTCACAGACGCGTTGAATTGTCCCCACGCCGCGCCCCTGTAGAGAAATATAAAAGGTTAGGATTTGCCACTGAGGTTCTTCTTTCATATACTTCCTTTTAAAATCTTGCTAGGATACAGTTCTCACATCACATCCGAACATAAACAACCATGGGTAAGGAAAAGACTCACGTTTCGAGGCCGCGATTAAATTCCAACATGGATGCTGATTTATATGGGTATAAATGGGCTCGCGATAATGTCGGGCAATCAGGTGCGACAATCTATCGATTGTATGGGAAGCCCGATGCGCCAGAGTTGTTTCTGAAACATGGCAAAGGTAGCGTTGCCAATGATGTTACAGATGAGATGGTCAGACTAAACTGGCTGACGGAATTTATGCCTCTTCCGACCATCAAGCATTTTATCCGTACTCCTGATGATGCATGGTTACTCACCACTGCGATCCCCGGCAAAACAGCATTCCAGGTATTAGAAGAATATCCTGATTCAGGTGAAAATATTGTTGATGCGCTGGCAGTGTTCCTGCGCCGGTTGCATTCGATTCCTGTTTGTAATTGTCCTTTTAACAGCGATCGCGTATTTCGTCTCGCTCAGGCGCAATCACGAATGAATAACGGTTTGGTTGATGCGAGTGATTTTGATGACGAGCGTAATGGCTGGCCTGTTGAACAAGTCTGGAAAGAAATGCATAAGCTTTTGCCATTCTCACCGGATTCAGTCGTCACTCATGGTGATTTCTCACTTGATAACCTTATTTTTGACGAGGGGAAATTAATAGGTTGTATTGATGTTGGACGAGTCGGAATCGCAGACCGATACCAGGATCTTGCCATCCTATGGAACTGCCTCGGTGAGTTTTCTCCTTCATTACAGAAACGGCTTTTTCAAAAATATGGTATTGATAATCCTGATATGAATAAATTGCAGTTTCATTTGATGCTCGATGAGTTTTTCTAATCAGTACTGACAATAAAAAGATTCTTGTTTTCAAGAACTTGTCATTTGTATAGTTTTTTTATATTGTAGTTGTTCTATTTTAATCAAATGTTAGCGTGATTTATATTTTTTTTCGCCTCGACATCATCTGCCCAGATGCGAAGTTAAGTGCG

TATAAGAGAACAAAAACACACGAAAAAAAAAAAGTCGTCAATATAAAAAGGAAAGAAATC

*FLC2* MAR 1and *FLC2* MAR 2:

GCTTAGAATAGCCGCCCAGTTTATATACAAAATGAAGGGAGAACTATTTGCCACCGAGGAACTaTACCCCAACTGCAATACCCATTGAATAATGGCATCGGAGGCTCGGCGGCAATTCGTACCCCAACCTTTTTTTTTTACTTTTCTTTGaATCTTAGAGATAACAGAAAAAAAGGATGACCCCAATCATTTGCCACGGCATGTCAACAGGTGAGTGCCTTTTGAGGGGGGGGGGTCATCTCGACATCCGGCGAAATGGAGCAGTCACACGTGAACATTTTTAGGGGATGGAGAGTGCTACGCCGTTCGTCCGAGATGATTATCATATTTACACAGCCGTACATACACGTGCCATTTATCTTGATATCATTCTGGACGTATGTGCACATGTGATTTGCTTTTGTTTTTTTAcGAATGTCGGGTAATAAACAGATTGTTTTTCTGGGAGGATAATCTTTTCTTTTTTCCTGTTGGTATTCTAAAATTAACCTTGCTGTTTCTTTTTTTTTTTTTTTTCGCGCGACTACTCAGCCATCTTGCATTTTTAAAGAAAAAGATAATCATgAATGCCTTCACGGGAATACGTATAGAACATTATTAAAAGTATATGAATGGCATATATATATAGAACACCACCCTTGGAAAACATTTATACCCCTTAAACTAAAACAATTTGCTGCGCTATACCGTGTTTCAcTGTATTATAATACATTCATTTCTGTTTCATTACGATTATATTGACGTGATAAAAAGATTATATAGCCATGATCTTCCTAAACACCTTCGCAAGGTGCCTTTTAACGTGTTTCGTACTGTGCAGCGGTACAGCACGTTCCTCTGACACAAACGACACTACTCCGGCGTCTGCAAAGCATTTGCAGACCACTTCTTTATTGACGTGTATGGACAATTCGCAATTAACGGCATCATTCTTTGATGTGAAATTTTACCCCGATAATAATACTGTTATCTTTGATATTGAtGCTACGACGACGCTTAATGGGAACGTCACTGTGAAGGCTGAGCTGCTTACTTACGGACTGAAAGTCCTGGATAAGACTTTTGATTTATGTTCCTTGGGCCAAGTATCGCTTTGCCCCCTAAGTGCTGGGCGTATTGATGTCATGTCCACACAGGTGATCGAATCATCCATTACCAAGCAATTTCCCGGCATTGCTTACACCATTCCAGATTTGGACGCACAAGTACGTGTGGTGGCATACGCTCAGAATGACACGGAATTCGAAACTCCGCTGGCTTGTGTCCAGGCTATCTTGAGTAACGGGAAGACAGTGCAAACAAAGTATGCGGCCTGGCCCATTGCCGCTATCTCAGGTGTCGGTGTACTTACCTCAGGGTTTGTGTCTGTGATCGGTTACTCAGCCACTGCTGCTCACATTGCGTCCAACTCCATCTCATTGTTCATATACTTCCAAAATCTAGCTATCACTGCAATGATGGGTGTCTCAAGGGTTCCACCCATTGCTGCCGCGTGGACGCAGAATTTCCAATGGTCCATGGGTATCATCAATACgAACTTCATGCAAAAGATTTTTGATTGGTACGTACAGGCCACTAATGGTGTCTCAAATGTTGTGGTAGCTAACAAGGACGTCTTGTCCATTAGTGTGCAAAAACGTGCTATCTCTATGGCATCGTCTAGTGATTACAATTTTGACACCATTTTAGACGATTCGAATCTGTACACCACTTCTGAGAAGGATCCAAGCAATTACTCAGCCAAGATTCTCGTGTTAAGAGGTATAGAAAGAGTTGCTTATTTGGCTAATATcGAGCTATCTAATTTCTTTTTGACCGGTATTGTGTTTTTcCTATTCTTCCTATTTGTAGTTGTCGTCTCTTTGATTTTCTTTAAGGCGCTATTGGAAGTTCTTACAAGAGCAAGAATcTTGAAAGAGACTTCCAATTTCTTCCAATATAGGAAGAACTGGGGGAGTATTATCAAAGGCACCCTTTTCAGATTATCTATCATCGCCTTCCCTCAAGTTTCTCTTCTGGCGATTTGGGAATTTACTCAGGTCAACTCTCCAGCGATTGTTGTTGATGCGGTgGTAATATTACTGATCATCACGGGACTTCTGGTTTATGGAACTATAAGGGTTTTCATCAAGGGAAGAGAGTCTCTCAGATTATACAAGAATCCTGCGTACCTACTTTACAGTGATACCTACTTCTTGAACAAGTTTGGGTTCTTATACGTTCAATTCAAAGCAGATAAGTTTTGGTGGCTTTTACCCTTATTAAGTTATGCGTTCTTAAGATCCCTGTTTGTTGCCGTTTTACAAAACCAAGGTAAGGCTCAAGCAATGATCATCTTTGTCATTGAACTAGCTTACTTCGTTTGTCTCTGTTGGATAAGACCgTATTTGGACAAGAGAACTAATGTTTTCAATATTGCTATTCATTTGGTGAATTTGATCAATGCATTTTTCTTTTTGTTTTTCAGTAATTTGTTCAAGCAACCAGCAGTGGTTTCGTCAGTGATGGCGGTTATTCTGTTCGTTTTGAACGCGGTGTTTGCTCTATTCCTATTATTGTTCACTATTGTCACCTGTACACTGGCATTACTACACAGAAACCCAGATGTCCGTTACCAACCAATGAAAGATGACCGTGTGTCATTCATTCCTAAGATTCAAAATGATTTCGATGGCAAAAACAAAAATGATTCTGAACTGTTTGAATTGAGgAAAGCTGTTATGGACACCAATGAAAATGAGGAAGAAAAAATGTTCCGTGAtGACACTTTCGGtAAGAACCTGAATGCAAACACAAATACAGCAAGACTCTTTGATGATGAGACTAGTTCATCCTCTTTTAAGCAAAATTCCTCTCCCTTCGATGCCTCGGAAGTAACGGAGCAACCTGTGCAACCAACCTCCGCTGTCATGGGTACGGGTGGCAGCTTCTTGTCTCCACAGTACCAACGTGCGTCATCTGCTTCTCGTACTAATCTAGCcCCGAATAATACAAGCACCTCCAGTTTAATGAAGCCTGAATCAAGTCTCTACCTGGGGAATTCCAATAAATCATATTCGCATTTTAACAACAACGGCAGCAACGAAAACGCCCGCAACAACAACCCATATTTGTAATCCAATATATACTCACATGTAACAACTTATTATATAAATATTTAAGGGCAAaGATATCCTACATTATATTTCATAGAAAACCGCTCAAAAAGGTGTATTATCTCCATTACATCCCAACACCACACCCTTGACAGTCTTGACGTGCGCAGCTCAGGGGCATGATGTGACTGTCGCCCGTACATTTA

*KanMX* amplification primer 3 and *FLC2* MAR 4:

CCTTGACAGTCTTGACGTGCGCAGCTCAGGGGCATGATGTGACTGTCGCCCGTACATTTAGCCCATACATCCCCATGTATAATCATTTGCATCCATACATTTTGATGGCCGCACGGCGCGAAGCAAAAATTACGGCTCCTCGCTGCAGACCTGCGAGCAGGGAAACGCTCCCCTCACAGACGCGTTGAATTGTCCCCACGCCGCGCCCCTGTAGAGAAATATAAAAGGTTAGGATTTGCCACTGAGGTTCTTCTTTCATATACTTCCTTTTAAAATCTTGCTAGGATACAGTTCTCACATCACATCCGAACATAAACAACCATGGGTAAGGAAAAGACTCACGTTTCGAGGCCGCGATTAAATTCCAACATGGATGCTGATTTATATGGGTATAAATGGGCTCGCGATAATGTCGGGCAATCAGGTGCGACAATCTATCGATTGTATGGGAAGCCCGATGCGCCAGAGTTGTTTCTGAAACATGGCAAAGGTAGCGTTGCCAATGATGTTACAGATGAGATGGTCAGACTAAACTGGCTGACGGAATTTATGCCTCTTCCGACCATCAAGCATTTTATCCGTACTCCTGATGATGCATGGTTACTCACCACTGCGATCCCCGGCAAAACAGCATTCCAGGTATTAGAAGAATATCCTGATTCAGGTGAAAATATTGTTGATGCGCTGGCAGTGTTCCTGCGCCGGTTGCATTCGATTCCTGTTTGTAATTGTCCTTTTAACAGCGATCGCGTATTTCGTCTCGCTCAGGCGCAATCACGAATGAATAACGGTTTGGTTGATGCGAGTGATTTTGATGACGAGCGTAATGGCTGGCCTGTTGAACAAGTCTGGAAAGAAATGCATAAGCTTTTGCCATTCTCACCGGATTCAGTCGTCACTCATGGTGATTTCTCACTTGATAACCTTATTTTTGACGAGGGGAAATTAATAGGTTGTATTGATGTTGGACGAGTCGGAATCGCAGACCGATACCAGGATCTTGCCATCCTATGGAACTGCCTCGGTGAGTTTTCTCCTTCATTACAGAAACGGCTTTTTCAAAAATATGGTATTGATAATCCTGATATGAATAAATTGCAGTTTCATTTGATGCTCGATGAGTTTTTCTAATCAGTACTGACAATAAAAAGATTCTTGTTTTCAAGAACTTGTCATTTGTATAGTTTTTTTATATTGTAGTTGTTCTATTTTAATCAAATGTTAGCGTGATTTATATTTTTTTTCGCCTCGACATCATCTGCCCAGATGCGAAGTTAAGTGCGATATTTCAGCGATAAAAACCTTAAATGTGAAATTCGCTTTGGCTCTGCTTCCTTAAATGT

*MGA1* MAR 1and *MGA1* MAR 2:

TTTCACCCACGCCATAAAATACAATAGCTCAGAGAACCGGTGGtCACATGCAACCTAAAACTTTCGTCCACCAACTTCATGCAATTCTTCTGGAACCGGAAGTAAAcAAATGGATATACTGGTCTCCGACTGATAACACGaTATTTTTTTTGAAGCCATAcGACCCTAATTTTAGTACACATGTTCTGAAGCGcTATTTCAAACATGGAAATGTCAATAGTTTTGTTCGTCAACTACACATGTACGGGTTTCATAAACTATCTCATCCTTCCCCAGACCAATCTTCTGCCAACAATGGTAACGTTAAAGAACTTGTTGAATGGAAATTTACtCACCCATCTGGATTCTTCTTCAAAaAGGCCAACGCCGGTATCTTGAATAAAATtCAAAGAAAGAGCACTGGTGTGGGAAAAGATGGTAAAAGAAAGAATATACTGTCACCAATATCTGTCAGTTACGTTGATGCTTCGAGGTTAAATGTTCTCTCTCAGCAATCCGGACCAGTTTCTGCAAGAGAACCATCAAAtATGTTTATGGGCAGTCCCGTCCATTACTCAACATCCCAAAGTCCTCCACATATCAGTATACCGCAACAGCAACAAAGTAGTGGACCTTACCTAATATCTTCACTGCCCCCTCAACAACCCACGGTGAACATGATGCGAAGGCAAAGCATCTCGGCGAGGATGATGAATTCTTATGACTATCCCAATCAATTTTCCACCCAAGATAGCATAGTCCAGCCTCAGCAGCCGCAACAAGTACTTTCTCCACAAGCATTATCTGGACCTCCGATGAAGAAATCAGGAACACTATCTTCGACAGATGATCTGAAAACAACTTCCTTGCCAATTGTTAATTACCCAATGCCGTATCATCCTGGAGCTTTTGCCCAGCAGCAGCAGCAGCAGCAGCAACCGCTCCCTACAGTCCCGCCATATAGCAGTTACTCCACTCCATTTCCCTCAATGATGAACTCTCTTTCGAACTCTGCTTCTAACTCACCTGCTTTAGGAGTTTGTAATAATAATGTAACACTACCCAAAAAAAGCAATATAAGTGAAAGGCAAGCTTTGGATAACCATATACAAACTTTAAAGAACTCTCTATCAACAATCACTGATTTGATAGAAAAACATATTAACAGCGCATCGCAAGATGAAAATAAAACTCTAACTAATGATGCCATGAACAAAGACCTTCGAACAAGCTTATCCCTATTACAGAATTCCAAGGAGGAAATCATTCAACTTGAAAGTAAATGGATGTCTATGCAATCTGTTAAAACAACTGCCCTACCCCTTCAAGAGACTACGAATACATCATCGACCTTAACTTCTCTGACGTCCAGCATAATTCCCAAGAGTATACCTATAATCACGAAAGGTGAAGTCGCCACTAAACCAGCATCTTACTGAATTATTTTCAACAGAACACATCGCATCCAACTGAACAAACTGTTACCGCTGTTGATACCAAGGCCTTGACAGTCTTGACGTGCGCAGCTCAGGGGCATGATGTGACTGTCGCCCGTACATTTA

*KanMX* amplification primer 3 and *MGA1* MAR 4:

CCTTGACAGTCTTGACGTGCGCAGCTCAGGGGCATGATGTGACTGTCGCCCGTACATTTAGCCCATACATCCCCATGTATAATCATTTGCATCCATACATTTTGATGGCCGCACGGCGCGAAGCAAAAATTACGGCTCCTCGCTGCAGACCTGCGAGCAGGGAAACGCTCCCCTCACAGACGCGTTGAATTGTCCCCACGCCGCGCCCCTGTAGAGAAATATAAAAGGTTAGGATTTGCCACTGAGGTTCTTCTTTCATATACTTCCTTTTAAAATCTTGCTAGGATACAGTTCTCACATCACATCCGAACATAAACAACCATGGGTAAGGAAAAGACTCACGTTTCGAGGCCGCGATTAAATTCCAACATGGATGCTGATTTATATGGGTATAAATGGGCTCGCGATAATGTCGGGCAATCAGGTGCGACAATCTATCGATTGTATGGGAAGCCCGATGCGCCAGAGTTGTTTCTGAAACATGGCAAAGGTAGCGTTGCCAATGATGTTACAGATGAGATGGTCAGACTAAACTGGCTGACGGAATTTATGCCTCTTCCGACCATCAAGCATTTTATCCGTACTCCTGATGATGCATGGTTACTCACCACTGCGATCCCCGGCAAAACAGCATTCCAGGTATTAGAAGAATATCCTGATTCAGGTGAAAATATTGTTGATGCGCTGGCAGTGTTCCTGCGCCGGTTGCATTCGATTCCTGTTTGTAATTGTCCTTTTAACAGCGATCGCGTATTTCGTCTCGCTCAGGCGCAATCACGAATGAATAACGGTTTGGTTGATGCGAGTGATTTTGATGACGAGCGTAATGGCTGGCCTGTTGAACAAGTCTGGAAAGAAATGCATAAGCTTTTGCCATTCTCACCGGATTCAGTCGTCACTCATGGTGATTTCTCACTTGATAACCTTATTTTTGACGAGGGGAAATTAATAGGTTGTATTGATGTTGGACGAGTCGGAATCGCAGACCGATACCAGGATCTTGCCATCCTATGGAACTGCCTCGGTGAGTTTTCTCCTTCATTACAGAAACGGCTTTTTCAAAAATATGGTATTGATAATCCTGATATGAATAAATTGCAGTTTCATTTGATGCTCGATGAGTTTTTCTAATCAGTACTGACAATAAAAAGATTCTTGTTTTCAAGAACTTGTCATTTGTATAGTTTTTTTATATTGTAGTTGTTCTATTTTAATCAAATGTTAGCGTGATTTATATTTTTTTTCGCCTCGACATCATCTGCCCAGATGCGAAGTTAAGTGCGAACATTCAGTGAACGTAGGGAAGAACGAACATTCTCCAT

*NOP19* MAR 1and *NOP19* MAR 2:

TCTTTCCTCGGTAGTGTTCAGAATGAGTAGGGCCAAAGAATTACAAGAGAAGCTCAACTTGCAAGCGAAACTCCAGTCTACTTTCAGTAACAATACTGCTGCcGTTTTGGACTGGCTGAAAGAATCAGATGAAACCGGCATTAGCAATGATACAGAGCGTAACAAACAGTTAAAAGATCACAAGGAGTTGGAGGATGGTAAGAAGGCATTTTTCAAGCTTCCCGTTTTGCAGATTGGATCGGGCCTACACTTCCGCACACAAGATGACGCTTCCGCCAAAGAAGATATACATACGATTGGTGAGTTTATTGAGGGTGATAAAAAGGTAAGTTCGTTGGCAAAGAAAAAGAAAAGAAGTGACCCAGGGTTGCAACGAAATAATATGTACAGGATCACTAAAGATGATACGAAAGCCATGATTGCGCTTAAAAGAAAAATGAGGAAAGGtGAGAAGGAAGGACTAAGGAAGAAACAAGAGCATAGCAAAAGCAaCGTTTCGAACTCATATAGTGCCAGTGATGAAGAGGATGAAGATGCAGGGACAATGCCGCAAAAGTCCACAAAAAAAAAATTTGGTTTACTTTTTGATAAAAAAAAGAAGGCACGTAAATAATCTTAAACACTTATGGGCAGCAAAAAATGCGTCTTTCTTCCCTCGTCTGTTGTTTTATGTAGGGCGTAATGATGTTTGC CCTTGACAGTCTTGACGTGCGCAGCTCAGGGGCATGATGTGACTGTCGCCCGTACATTTA

*KanMX* amplification primer 3 and *NOP19* MAR 4:

CCTTGACAGTCTTGACGTGCGCAGCTCAGGGGCATGATGTGACTGTCGCCCGTACATTTAGCCCATACATCCCCATGTATAATCATTTGCATCCATACATTTTGATGGCCGCACGGCGCGAAGCAAAAATTACGGCTCCTCGCTGCAGACCTGCGAGCAGGGAAACGCTCCCCTCACAGACGCGTTGAATTGTCCCCACGCCGCGCCCCTGTAGAGAAATATAAAAGGTTAGGATTTGCCACTGAGGTTCTTCTTTCATATACTTCCTTTTAAAATCTTGCTAGGATACAGTTCTCACATCACATCCGAACATAAACAACCATGGGTAAGGAAAAGACTCACGTTTCGAGGCCGCGATTAAATTCCAACATGGATGCTGATTTATATGGGTATAAATGGGCTCGCGATAATGTCGGGCAATCAGGTGCGACAATCTATCGATTGTATGGGAAGCCCGATGCGCCAGAGTTGTTTCTGAAACATGGCAAAGGTAGCGTTGCCAATGATGTTACAGATGAGATGGTCAGACTAAACTGGCTGACGGAATTTATGCCTCTTCCGACCATCAAGCATTTTATCCGTACTCCTGATGATGCATGGTTACTCACCACTGCGATCCCCGGCAAAACAGCATTCCAGGTATTAGAAGAATATCCTGATTCAGGTGAAAATATTGTTGATGCGCTGGCAGTGTTCCTGCGCCGGTTGCATTCGATTCCTGTTTGTAATTGTCCTTTTAACAGCGATCGCGTATTTCGTCTCGCTCAGGCGCAATCACGAATGAATAACGGTTTGGTTGATGCGAGTGATTTTGATGACGAGCGTAATGGCTGGCCTGTTGAACAAGTCTGGAAAGAAATGCATAAGCTTTTGCCATTCTCACCGGATTCAGTCGTCACTCATGGTGATTTCTCACTTGATAACCTTATTTTTGACGAGGGGAAATTAATAGGTTGTATTGATGTTGGACGAGTCGGAATCGCAGACCGATACCAGGATCTTGCCATCCTATGGAACTGCCTCGGTGAGTTTTCTCCTTCATTACAGAAACGGCTTTTTCAAAAATATGGTATTGATAATCCTGATATGAATAAATTGCAGTTTCATTTGATGCTCGATGAGTTTTTCTAATCAGTACTGACAATAAAAAGATTCTTGTTTTCAAGAACTTGTCATTTGTATAGTTTTTTTATATTGTAGTTGTTCTATTTTAATCAAATGTTAGCGTGATTTATATTTTTTTTCGCCTCGACATCATCTGCCCAGATGCGAAGTTAAGTGCGTTGTCAACAAATGAATACGTACAGAAGAGAATTCTAGCCAAGGCAATTATTGCATACTGC

*YGR250C* MAR 1and *kanMX* amplification primer 2:

GTAAAGAATGAAGAAAAAAGGAGTAAAAAGTATGAATAAGATAAATGAAAATATAAAAATCCTTGACAGTCTTGACGTGCGCAGCTCAGGGGCATGATGTGACTGTCGCCCGTACATTTAGCCCATACATCCCCATGTATAATCATTTGCATCCATACATTTTGATGGCCGCACGGCGCGAAGCAAAAATTACGGCTCCTCGCTGCAGACCTGCGAGCAGGGAAACGCTCCCCTCACAGACGCGTTGAATTGTCCCCACGCCGCGCCCCTGTAGAGAAATATAAAAGGTTAGGATTTGCCACTGAGGTTCTTCTTTCATATACTTCCTTTTAAAATCTTGCTAGGATACAGTTCTCACATCACATCCGAACATAAACAACCATGGGTAAGGAAAAGACTCACGTTTCGAGGCCGCGATTAAATTCCAACATGGATGCTGATTTATATGGGTATAAATGGGCTCGCGATAATGTCGGGCAATCAGGTGCGACAATCTATCGATTGTATGGGAAGCCCGATGCGCCAGAGTTGTTTCTGAAACATGGCAAAGGTAGCGTTGCCAATGATGTTACAGATGAGATGGTCAGACTAAACTGGCTGACGGAATTTATGCCTCTTCCGACCATCAAGCATTTTATCCGTACTCCTGATGATGCATGGTTACTCACCACTGCGATCCCCGGCAAAACAGCATTCCAGGTATTAGAAGAATATCCTGATTCAGGTGAAAATATTGTTGATGCGCTGGCAGTGTTCCTGCGCCGGTTGCATTCGATTCCTGTTTGTAATTGTCCTTTTAACAGCGATCGCGTATTTCGTCTCGCTCAGGCGCAATCACGAATGAATAACGGTTTGGTTGATGCGAGTGATTTTGATGACGAGCGTAATGGCTGGCCTGTTGAACAAGTCTGGAAAGAAATGCATAAGCTTTTGCCATTCTCACCGGATTCAGTCGTCACTCATGGTGATTTCTCACTTGATAACCTTATTTTTGACGAGGGGAAATTAATAGGTTGTATTGATGTTGGACGAGTCGGAATCGCAGACCGATACCAGGATCTTGCCATCCTATGGAACTGCCTCGGTGAGTTTTCTCCTTCATTACAGAAACGGCTTTTTCAAAAATATGGTATTGATAATCCTGATATGAATAAATTGCAGTTTCATTTGATGCTCGATGAGTTTTTCTAATCAGTACTGACAATAAAAAGATTCTTGTTTTCAAGAACTTGTCATTTGTATAGTTTTTTTATATTGTAGTTGTTCTATTTTAATCAAATGTTAGCGTGATTTATATTTTTTTTCGCCTCGACATCATCTGCCCAGATGCGAAGTTAAGTGCG

*YGR250C* MAR 3 and *YGR250C* MAR 4:

AGCGTGATTTATATTTTTTTTCGCCTCGACATCATCTGCCCAGATGCGAAGTTAAGTGCGAAAAACCAACTAATACATGAAGAAAAAAAAGCAGACAAAAACATTTTATGGACCTGATGCAATCTAGTAGTCCATAGAATAATCACCACcAGAAAATTCTTCCTCTTCATTACTACCGTTTGCCATTATAGGAATATGATTTGCTGCAGGATTCTGCGGAGGTATTATATAGGGCACTGGCGGCACCTGTGGAATAAACCCAAATGATGGGAACATTGGCATCATCCAGTTAGCGTTATTTTGGTTTGCACTTATTAAGTTGTAACTGTTCACGGGCTTTGTGTTGGTATTAGGGTACTGCAGTGGTATGAAATAATTTTCCCTCGAGACTTGCTGTTGCGATTGGTTCTGAGTCATCAAGGCATTGATAAATTTCTTATTATACTTTGTTGGATAAGCCTTATGATAATCATTATGTGGCTGAAAATTTTGGAACTTTGATGAGTTATCAGTTTGGGATTGATCATCATCATCACTaGAAGATAAATTACCACGTTTCTGAGCGAAAGAAACAGATAACACTTGATCTTTGCTCACTTGATACCCGTCTGTATTCAAAATTGCCTTAGCAGCATCTAATGGGCTTTCAAAAGAAACAAAACCATAACCTCTTGATGATCCCACTGGCAGATCATTATCTGATGAGCTATCATTCGATTGTTGACGATACTTGTTCTTACTACCCCCAACAGTAATGACCTTAACGCTGATTATTTCACCGAAAGATTTGTAGAAATCATATAAATCTTCATCTGTCCAAGAAAGAGGGATGTGCTTGACATATgGGTTTGATTCTTGCTGATTACTCATTGGTATAGGTAAGCTGTAAGAAGTTGATGGGTACTGGTACATGTCAAAtTTGTTGCCCATGAATCTAGGCACACGTTGACCgCGACCTTCACTGAAAGATTCCCTTTGAGGATAGAACAGATCCATTTTCTCTGCCACAGGGGTTGATCTTACAGAATCAAAATACCGTACATTGGAGGTATCGTTGCTGTTCCCGTTTTTATTATTGTTGAAGTAATATCTATCATATGTATAATTATTGTAATCATACATTGGGTTATTTGCAAATTCCAAAACCTCCATATCATTGTGGTTACTGATGGAAGGGTGCCTGTCATGGCTATTGTGGTTGTTGTAAACTTTATGTTGAACCGCCTTATTAACAACGAGCCTGTTTCCATGCCACATGAAtCCATTGAAGGTCTCGATGGCTGCTAGTGCTTGTTCATGGTTGATAAGCTTGATGAAACCATAACCCTTCAACATATCTTCATCTTGGGCATTTCCATTAGTATTATTGGAAGATTTGTTTGATTCTACTGACCCCTCCTCATTGAAACTTACTGAACTACTGCTTCTTGTATTACTTCTCTTCGGAAAGTAATATGAAATGATATCAAAGTCCGGAAACTTTTTTGATAACTCCTTCTTGATTACTTCTATGACTTCTTTAGGTGTAATCAAAGTCTCTACTTTTTCAGGATTGTGATAAGGCAAGTTGCCTATAAAGAGACACCTGAAATTATCATCATTGTTGTTTTCTTTGACATGGTCCCAATGGATTCTTTCTCTCTCCTTTCGCTCAACGTGATAGTTGATAAATAGTGTGGAGCCATTTACCGTCCTTCCATTAAGTTCCTTGATGCAAAGTGAAGCTTGAGAACCCAAGGGGTAGGAGATGAACCCGTATCCGTTAGGTTCGCCTTTCGTTTTATCATATATCAATTTCATTGATAAAATTGGTCCATATTTTGAAAATAAGAAACTcAGTTCACCAATAGAAAGGCTCTTTGCTATTCCCCCGACAAAAATATTcCCAGGATAGGTTAGTGCATGCTTGTTCACACCGATAGACCATTTATCAATATGCTGACCAATAGGTTCGCTCTTGAGTAATTCaGAGATATCTGTACATGCATTTTCTAATGACTTGGGGTACTTGAATTGTGCTTGCAAAACGGTATCGTATCTCCAATCATCTTCCTCTTCGTTATCTTTGTAAGATTTCTCATACTCTTGACTTCCATGCTCGTAAAGATAGATGTCTTTATTTTGTTCGAGAACCTGTAACCTCtTTGTATCCGGAAGAAATTCATATGTTTCTGTATCAGAGTTGTTAACGGAGATACCTTTGTAGTGCTGTATTGTGTCCATTATTTGATCGGTTATTCTAGAGTTGCAGTGATCATCTTCTTCTTCTTGTAACTGCCATTTTATTCTTATGGTTAATAAATTTGTGTTGGCCAATTGTTCCAGTTGAAGACCAGTGGAAGTTTTCCTTTCAATTTTTATTGATTGGTCTCCAGCTTCTGCGCTCGCTGATGTCTGCTGGCTGCAGATATCTGTATCCTCGGGGCCACTAGA

*BIT61* MAR 1and *kanMX* amplification primer 2:

GACAGACCTTGGTGTAGGATGGAATAAAGTTTTGTTGTGATATTAAATACCTTGACAGTCTTGACGTGCGCAGCTCAGGGGCATGATGTGACTGTCGCCCGTACATTTAGCCCATACATCCCCATGTATAATCATTTGCATCCATACATTTTGATGGCCGCACGGCGCGAAGCAAAAATTACGGCTCCTCGCTGCAGACCTGCGAGCAGGGAAACGCTCCCCTCACAGACGCGTTGAATTGTCCCCACGCCGCGCCCCTGTAGAGAAATATAAAAGGTTAGGATTTGCCACTGAGGTTCTTCTTTCATATACTTCCTTTTAAAATCTTGCTAGGATACAGTTCTCACATCACATCCGAACATAAACAACCATGGGTAAGGAAAAGACTCACGTTTCGAGGCCGCGATTAAATTCCAACATGGATGCTGATTTATATGGGTATAAATGGGCTCGCGATAATGTCGGGCAATCAGGTGCGACAATCTATCGATTGTATGGGAAGCCCGATGCGCCAGAGTTGTTTCTGAAACATGGCAAAGGTAGCGTTGCCAATGATGTTACAGATGAGATGGTCAGACTAAACTGGCTGACGGAATTTATGCCTCTTCCGACCATCAAGCATTTTATCCGTACTCCTGATGATGCATGGTTACTCACCACTGCGATCCCCGGCAAAACAGCATTCCAGGTATTAGAAGAATATCCTGATTCAGGTGAAAATATTGTTGATGCGCTGGCAGTGTTCCTGCGCCGGTTGCATTCGATTCCTGTTTGTAATTGTCCTTTTAACAGCGATCGCGTATTTCGTCTCGCTCAGGCGCAATCACGAATGAATAACGGTTTGGTTGATGCGAGTGATTTTGATGACGAGCGTAATGGCTGGCCTGTTGAACAAGTCTGGAAAGAAATGCATAAGCTTTTGCCATTCTCACCGGATTCAGTCGTCACTCATGGTGATTTCTCACTTGATAACCTTATTTTTGACGAGGGGAAATTAATAGGTTGTATTGATGTTGGACGAGTCGGAATCGCAGACCGATACCAGGATCTTGCCATCCTATGGAACTGCCTCGGTGAGTTTTCTCCTTCATTACAGAAACGGCTTTTTCAAAAATATGGTATTGATAATCCTGATATGAATAAATTGCAGTTTCATTTGATGCTCGATGAGTTTTTCTAATCAGTACTGACAATAAAAAGATTCTTGTTTTCAAGAACTTGTCATTTGTATAGTTTTTTTATATTGTAGTTGTTCTATTTTAATCAAATGTTAGCGTGATTTATATTTTTTTTCGCCTCGACATCATCTGCCCAGATGCGAAGTTAAGTGCG

*BIT61* MAR 3 and *BIT61* MAR 4:

AGCGTGATTTATATTTTTTTTCGCCTCGACATCATCTGCCCAGATGCGAAGTTAAGTGCGGGAGATTGAAATAAAGTGTTAGATGCTTTCTATATACTCATTCTAATGCCTGCTAATAACTCTTCGATAATTCGTTGATTTCTATCATTACTTTGAATGGTACTCAATATGCCAAAACACTGCAGTAATGTCAATTTATCTGTTTCTGTGACATTACTTTCTTCTTCTTGATTGAAAATGTAAAGTTGAAAACTTTTGCTGATACCATCaTTACTATTTACGAAGTTTTCATAACAAGGTAAGACTATTGAATCTCTAAAGCACATAAGCAAAAGAAACCCAATAGAATAATTACCATTAATGTGAGTCCGACTTTCATTGCCTGACTTTAAAACCGTAGAATTACCATCTTCTCTGATACTCTGATCCAACGGTAATAACACAGCGGCTAAATCATAATATACCTCTTGATAAAATATCCGCCATATAACGCCTAATCTTTTCAATGCGTTATTTATTGTGTCTTCATTAGAATAGTTAAAAACAATTTGGTTTTCTAAATTGTATAGGGAACTAACCAAAAATTCTTTCAAATCGCTGATAATTCTATTCATGCCGCTATGTGTGACTCTTGGATCTGTTTTTAAAATGCAATAGAATTCTAAAACTTCGTTAAAATTTTCTATTTTGGCCCTGAGTTTCCATATTCTTTGAGTTCTGAATAATTCTGCTACGTTACTACAAAATATATTCCAAATATTATTTGTTAGGGTTGTCAGTTCTTGGGAGTTGAAGTGTTTATAGTTAGCTTCCAGAGATGGTAAATTTCTTAAAAGGTCGTGTAACATTGAGACCGGATTCGGTGAGGCAGGATCAGTTCCAGAAAGCGGATCCTCAACGATTGTGTTGGGATTGACACCAGATGGATTAACATTCAATGAGTACACAGAAGTTCCTGAGTCCACAAGTTGCCCCGCATTAGTATGAATAAATTGTGACGGAGATTTATGTCTTCCGTAAGAGGAATGCAGAAATTTACTCAAGGAATTTGGAATTGCAGGCTCGATAGCATCTTGACCAATGTGCCTGTGGCTTTTATTTTGGAAAAAGTTCTTAATCACGTTTCGAAATGAATGTGAACTATTATGTAAGGTGCTTGAATCTGTCAAAAAGGAGGCAGGATCCGATGAAATGGTCGTATGTGCTCTGTTTCTCCTATTAAAAAAGCCAGAAGCCTTTCCTTTCATATACCTGCCGGAGGGCGTGGAGTGTAAACTTTTCTGAGTAATGGATATTGCATCAGAAGATAACTTTTTAGATAGATTGTTCGAGCACTCCACCGTGTCAAtGCTATGAGATTCCCCTATCTGAGAAGAACCAGATTTAGATGTTGCAGCTGAGGCATTGTCATTGCTCCTCAAGGAATGATTTGAGGCCACACTGACCTTCGATCGAGGATGATCTACTGTATGGAATATGGATTGGAAGCCAACGTTAGAAACACTAGGGCTAATGGGGTTCCTCGAGCCCTTCAAATTCTTGATGGAATAAACGTCATCATTTGATGTGTCTACAAGAGTTTGCCGGCTAATCTCGCTTGTAGTCTGAAAATTTTTTACTGGGGCTGTGGaTAATTGCACATTAAGGTATTGTTCGCTATTGACAGGGCGTTGTGTCGTAGTAGATGTTCTTTCTCGAgGGAGTATATCTTCTGCTGTCATAATGAAAGAACTTTATTTCTACAGAGGTCAATTGGCG

*IKS1* MAR 1and *kanMX* amplification primer 2:

CGTGAACATTGGTGATAAGCGAGAATATTTTCTATAAATTTGTAAAGTAAAAATAAATTACCTTGACAGTCTTGACGTGCGCAGCTCAGGGGCATGATGTGACTGTCGCCCGTACATTTAGCCCATACATCCCCATGTATAATCATTTGCATCCATACATTTTGATGGCCGCACGGCGCGAAGCAAAAATTACGGCTCCTCGCTGCAGACCTGCGAGCAGGGAAACGCTCCCCTCACAGACGCGTTGAATTGTCCCCACGCCGCGCCCCTGTAGAGAAATATAAAAGGTTAGGATTTGCCACTGAGGTTCTTCTTTCATATACTTCCTTTTAAAATCTTGCTAGGATACAGTTCTCACATCACATCCGAACATAAACAACCATGGGTAAGGAAAAGACTCACGTTTCGAGGCCGCGATTAAATTCCAACATGGATGCTGATTTATATGGGTATAAATGGGCTCGCGATAATGTCGGGCAATCAGGTGCGACAATCTATCGATTGTATGGGAAGCCCGATGCGCCAGAGTTGTTTCTGAAACATGGCAAAGGTAGCGTTGCCAATGATGTTACAGATGAGATGGTCAGACTAAACTGGCTGACGGAATTTATGCCTCTTCCGACCATCAAGCATTTTATCCGTACTCCTGATGATGCATGGTTACTCACCACTGCGATCCCCGGCAAAACAGCATTCCAGGTATTAGAAGAATATCCTGATTCAGGTGAAAATATTGTTGATGCGCTGGCAGTGTTCCTGCGCCGGTTGCATTCGATTCCTGTTTGTAATTGTCCTTTTAACAGCGATCGCGTATTTCGTCTCGCTCAGGCGCAATCACGAATGAATAACGGTTTGGTTGATGCGAGTGATTTTGATGACGAGCGTAATGGCTGGCCTGTTGAACAAGTCTGGAAAGAAATGCATAAGCTTTTGCCATTCTCACCGGATTCAGTCGTCACTCATGGTGATTTCTCACTTGATAACCTTATTTTTGACGAGGGGAAATTAATAGGTTGTATTGATGTTGGACGAGTCGGAATCGCAGACCGATACCAGGATCTTGCCATCCTATGGAACTGCCTCGGTGAGTTTTCTCCTTCATTACAGAAACGGCTTTTTCAAAAATATGGTATTGATAATCCTGATATGAATAAATTGCAGTTTCATTTGATGCTCGATGAGTTTTTCTAATCAGTACTGACAATAAAAAGATTCTTGTTTTCAAGAACTTGTCATTTGTATAGTTTTTTTATATTGTAGTTGTTCTATTTTAATCAAATGTTAGCGTGATTTATATTTTTTTTCGCCTCGACATCATCTGCCCAGATGCGAAGTTAAGTGCG

*IKS1* MAR 3 and *IKS1* MAR 4:

AGCGTGATTTATATTTTTTTTCGCCTCGACATCATCTGCCCAGATGCGAAGTTAAGTGCGCGTGCTATTTAGTTTGCAATGTGTACTTATTGTTAGTATATGTATTTTTTGCATGCGGCGATGAAGGCCAGTAAAACCAACGCTCTTGCATGCTTACCTCTTTCATCAGCTGGTGATTTAAAAACCATTCCCAACAATATCAGTGACATATAAGAGAGCCACGACCCCGTTTTGGTACACTTAAATATGATTATCGTTAATATCATAGAAACTAGTTTGTAACAAACCTGGATTGTTCTATTTAAAGCAGAATACTTTTGGAGGTTTTGGGTTGATACGGTGCTCAAATTTTCCTCTGGTGCTGGTAATGATAAAGTAACGTTGTCTCCGTCAATATAATCATCAGTAAAACTGTTGGTATTCTCgTTCACTTCCGAGATTGTACTGAAGTTCAAAGTGCTATCCACATTTTCTTTCCAGAATTTTTTGCCGGGTTTGGAATTTATTAGCATTTCATCTAACGTCTCCTCCACTGTCTTCGCTGTGGGTCTAGCATCATTATTcGGTTGTAAAAGAGCGTCCATTAGGTGAAATATTCTACGGTCTATTGGTTTCAATTTCATCGCTTGATGCTTTTCAATCATACCTTCAGTGTCGAACCTAAAATTTTTGATGCGTACCTTCAAATCAACTATATCCAACTGTGGTTCAAAGGGCAATTCACCGAAGACAATAAAATAGCAAATCATTCCTAGAGAATACATATCGGAAGCAAAGGTGTATTCATTGTATGTGTGACTCGATCTTGAAGGCAACGTAGAAGAAGAAACTGGTCTCCCCTGGATAATAAGtTCTGGTGCTGTAAACTCTAATGTTCCTGTGCAACCAGTACCCAATCTGGACTCTCCTTCCAACTGACTTTCCCCCAAATCGCCAATAACAATTGATGGGAAAAACTCATCTGAATTGTGTTCCCTGTCGTATACGTTGTCATTCTCATTGTCACTTTTAAATGGAGTCAATAATAAACAGTTGGATGGTTTCAAGTCCCTATGTATTAGGCCAATACTGTGCAGTTCGTGTAAACCTCTTGCAATATCCCTTATAATaaATACGAGCTGTTCCGTAGATAGGCCAACTTCTCCTGATTTACCGTGATTTTTCTTaCGAGTTCTAAATTTTTTTTTCCTCTCTTCAGGTGATTCGGTATCTGAGAACCTATTGAACACTTTTCTTAGAATACAGTCTTCTAAATTGCCGCCACTGCAATACTGTTGTAAAATGAAAATACAAGGAAtTTCTTCTTGTGAATCCGATTGGCTCCCATCAATGGATCTGACAAATCCAACCGAAGAATCCATTTCGAGCCACACATGATTATACGTAATCAAATTGGCACTTTTGTGAGTTAGCGAACTCAACGCTTTCACTTCCCGTATGCACTTATTAAACCATTCCATATCATTTCCAATAGGTATTTTTTTTAGTGCAAAAACACCTAATTCAGTATTACCAATTGTGTGTACAACTTTGTAAACAGAACCTCTTGCACCATTTCCCAGCAAAGACAATATTTTGAAAAATTTACGAAAGTATCCGGGGATAAAtAAATCATCTGGAATAAAGTATTTGTTTTTGCTGAACTGTCCCTGCTTATTGTTACTATCGTTTTGTAGGGCGTAGTGCCGATGATTTCTTTCCAATaACTGAAAATATCTTCTTGAAAAGCTTGAATTGGCAAATTCAAATCCAAGTGGCACAGTGTTCTCTGGAATACTGAGCCTACTGCTTTCTGATTCTACTCCAGCAGAGGCTCGACGATGAAGTTGTCTTCTGTTTATAATATCAGGGTTAATTTCCGTACCACATTGCGGGCACACATAGGAAGCAATAGGGCTTTTGTATTGATGAATCCCTGAAGGAAAGTCCAGTGCTGATAAAGATGCAGTCTGGTCTTCATTTAGTTCAGAGTCATcATTACTGTTGTCCTGTTGGAAAAATGATAAAGTTCCTGAGGTTGGATTgACAACCACCACTGATTTAGAATTAGGATCATCTAAAATCAGCGAACCTTCTTCATATGGTACTAAACTCATTTCTAAGTTCTACTTATGCTATtCCTCAAACTATATATCTAATAGTGTTTCTTCGGTTACCTAGTTGTCgCTCATAGGATCTATATATACAATGTCCTTCCCCCCCACCCCCTTTTCTGAAACGTATACAAAAAATAAAGCTTGAAATACGTTATTACGTAAAAAgAGTGTACTGTAAAAGCTTACAAAAAAGAGTTTGCGAAAAATTATAAATAAAAAGTAGTCTAGGTAAAATATGAAATGTTTCTAATTATGCATTTATGTGATCCTGAAAACCGCCCTTCTTACGTT

*RPS5* MAR 1and *RPS5* MAR 2:

GAAATAACCGACCATTCCAAAGATGTCTGACACCGAAGCTCCAGTTGAAGTTCAAGAAGATTTCGAAGTTGTTGAAGAATTCACCCCAGTCGTCTTGGCTACTCCAATTCCAGAAGAAGTCCAACAAGCTCAAACCGAGATTAAGTTGTTCAACAAATGGTCTTTTGAAGAAGTTGAAGTTAAGGATGCTTCTTTGGTTGACTACGTTCAAGTTAGACAACCAATCTTTGTTGCTCACACCGCTGGTCGTTACGCCAACAAGAGATTCAGAAAGGCTCAATGTCCAATCATTGAAAGATTGACCAACTCCTTGATGATGAACGGTAGAAACAACGGTAAGAAATTAAAGGCTGTTAGAATCATCAAGCACACTTTGGACATCATCAATGTCTTGACTGACCAAAACCCAATCCAAGTTGTTGTTGACGCTATCACCAACACTGGTCCAAGAGAAGACACCACCAGAGTCGGTGGTGGTGGTGCcGCTAGACGTCAAGCTGTCGATGTTTCTCCATTGAGAAGAGTTAACCAAGCTATTGCTTTGTTGACCATTGGTGCCAGAGAAGCTGCTTTCAGAAACATCAAGACCATTGCTGAAACTTTGGCCGAAGAATTGATCAATGCTGCTAAGGGTTCCTCTACTTCTTACGCTATCAAGAAGAAGGATGAATTGGAACGTGTTGCCAAGTCTAACCGTTAAGAAGCTAAAAAAgGTGAAAGATTTTCAATATTACATATGTTTTTCTTATTATTACTTTTATCTTCTTTGTACACTCTATTGTTTAAATTTATAACATAATACAATCTTATCTTTTGATGTAAAAAAAAATAGGACTATCAATTTTTAAATAATATATCTTACGCAAATCTATCAAGTAAATATAAATGCTATTGGTTGTACTTCCTCGTGTGTCAGATACTTTAACTGGGTACTATTTACATTGTCaCTAAACGTAACGTTTCCTTGAAATGATCTACACATGTTTCTAAGTCACTCTACATTTGCTGCTGCACCTTGACAGTCTTGACGTGCGCAGCTCAGGGGCATGATGTGACTGTCGCCCGTACATTTA

*KanMX* amplification primer 3 and *RPS5* MAR 4:

CCTTGACAGTCTTGACGTGCGCAGCTCAGGGGCATGATGTGACTGTCGCCCGTACATTTAGCCCATACATCCCCATGTATAATCATTTGCATCCATACATTTTGATGGCCGCACGGCGCGAAGCAAAAATTACGGCTCCTCGCTGCAGACCTGCGAGCAGGGAAACGCTCCCCTCACAGACGCGTTGAATTGTCCCCACGCCGCGCCCCTGTAGAGAAATATAAAAGGTTAGGATTTGCCACTGAGGTTCTTCTTTCATATACTTCCTTTTAAAATCTTGCTAGGATACAGTTCTCACATCACATCCGAACATAAACAACCATGGGTAAGGAAAAGACTCACGTTTCGAGGCCGCGATTAAATTCCAACATGGATGCTGATTTATATGGGTATAAATGGGCTCGCGATAATGTCGGGCAATCAGGTGCGACAATCTATCGATTGTATGGGAAGCCCGATGCGCCAGAGTTGTTTCTGAAACATGGCAAAGGTAGCGTTGCCAATGATGTTACAGATGAGATGGTCAGACTAAACTGGCTGACGGAATTTATGCCTCTTCCGACCATCAAGCATTTTATCCGTACTCCTGATGATGCATGGTTACTCACCACTGCGATCCCCGGCAAAACAGCATTCCAGGTATTAGAAGAATATCCTGATTCAGGTGAAAATATTGTTGATGCGCTGGCAGTGTTCCTGCGCCGGTTGCATTCGATTCCTGTTTGTAATTGTCCTTTTAACAGCGATCGCGTATTTCGTCTCGCTCAGGCGCAATCACGAATGAATAACGGTTTGGTTGATGCGAGTGATTTTGATGACGAGCGTAATGGCTGGCCTGTTGAACAAGTCTGGAAAGAAATGCATAAGCTTTTGCCATTCTCACCGGATTCAGTCGTCACTCATGGTGATTTCTCACTTGATAACCTTATTTTTGACGAGGGGAAATTAATAGGTTGTATTGATGTTGGACGAGTCGGAATCGCAGACCGATACCAGGATCTTGCCATCCTATGGAACTGCCTCGGTGAGTTTTCTCCTTCATTACAGAAACGGCTTTTTCAAAAATATGGTATTGATAATCCTGATATGAATAAATTGCAGTTTCATTTGATGCTCGATGAGTTTTTCTAATCAGTACTGACAATAAAAAGATTCTTGTTTTCAAGAACTTGTCATTTGTATAGTTTTTTTATATTGTAGTTGTTCTATTTTAATCAAATGTTAGCGTGATTTATATTTTTTTTCGCCTCGACATCATCTGCCCAGATGCGAAGTTAAGTGCGTTTAATGGCGGTTCATTTGCTTTTTAATTTTAGCGTCC

*YJR124C* MAR 1and *kanMX* amplification primer 2:

GGTACTATTTACATTGTCtCTAAACGTAACGTTTCCTTGAAATGATCTACACATGTTTCTCCTTGACAGTCTTGACGTGCGCAGCTCAGGGGCATGATGTGACTGTCGCCCGTACATTTAGCCCATACATCCCCATGTATAATCATTTGCATCCATACATTTTGATGGCCGCACGGCGCGAAGCAAAAATTACGGCTCCTCGCTGCAGACCTGCGAGCAGGGAAACGCTCCCCTCACAGACGCGTTGAATTGTCCCCACGCCGCGCCCCTGTAGAGAAATATAAAAGGTTAGGATTTGCCACTGAGGTTCTTCTTTCATATACTTCCTTTTAAAATCTTGCTAGGATACAGTTCTCACATCACATCCGAACATAAACAACCATGGGTAAGGAAAAGACTCACGTTTCGAGGCCGCGATTAAATTCCAACATGGATGCTGATTTATATGGGTATAAATGGGCTCGCGATAATGTCGGGCAATCAGGTGCGACAATCTATCGATTGTATGGGAAGCCCGATGCGCCAGAGTTGTTTCTGAAACATGGCAAAGGTAGCGTTGCCAATGATGTTACAGATGAGATGGTCAGACTAAACTGGCTGACGGAATTTATGCCTCTTCCGACCATCAAGCATTTTATCCGTACTCCTGATGATGCATGGTTACTCACCACTGCGATCCCCGGCAAAACAGCATTCCAGGTATTAGAAGAATATCCTGATTCAGGTGAAAATATTGTTGATGCGCTGGCAGTGTTCCTGCGCCGGTTGCATTCGATTCCTGTTTGTAATTGTCCTTTTAACAGCGATCGCGTATTTCGTCTCGCTCAGGCGCAATCACGAATGAATAACGGTTTGGTTGATGCGAGTGATTTTGATGACGAGCGTAATGGCTGGCCTGTTGAACAAGTCTGGAAAGAAATGCATAAGCTTTTGCCATTCTCACCGGATTCAGTCGTCACTCATGGTGATTTCTCACTTGATAACCTTATTTTTGACGAGGGGAAATTAATAGGTTGTATTGATGTTGGACGAGTCGGAATCGCAGACCGATACCAGGATCTTGCCATCCTATGGAACTGCCTCGGTGAGTTTTCTCCTTCATTACAGAAACGGCTTTTTCAAAAATATGGTATTGATAATCCTGATATGAATAAATTGCAGTTTCATTTGATGCTCGATGAGTTTTTCTAATCAGTACTGACAATAAAAAGATTCTTGTTTTCAAGAACTTGTCATTTGTATAGTTTTTTTATATTGTAGTTGTTCTATTTTAATCAAATGTTAGCGTGATTTATATTTTTTTTCGCCTCGACATCATCTGCCCAGATGCGAAGTTAAGTGCG

*YJR124C* MAR 3 and *YJR124C* MAR 4:

AGCGTGATTTATATTTTTTTTCGCCTCGACATCATCTGCCCAGATGCGAAGTTAAGTGCGAAGTCACTCTACATTTGCTGCTGCATTAATGGCGGTTCATTTGCTTTTTAATTTTAGCGTCCACTCCTAAAAACATGCATGCCAGTATTAGATCCGCCGTTATCACCAAGGACCCACTAATGATGTAACATAGCCATAAATAATTATTGTTAGCAAGTAcACCCGTGAATATTGGACCAACACACCGAGCAAATGTCTTTCCAATGTTGACCAtCCCCATAACTTTGGTTAAATCTCTTGGTTTGATGATATTTGTTAATAGAATTTGCCTTGGTGTAACGTCCATGGCAGTTGTTGCAAAATGCAGATTCAAAAACAAAATAGATAAGGGCAAGTAATTCTTGGCCATAGGGATGAGAATAGAAAATATCCCTGATGGAATTTGGACCAATAGTGTGGCTCTTACTGGGCCAAAAtATCTGGCAATTATTGATGATGGGATGGTGGAAGATGCCATAACCAGTTGCGTGATGAAAAATAAAGTACCCAACGCCAGAGATCCCATCAAAAATTGCTTACTATAGTAGTAAACCATCCAGCCACTTGTCATAAACCCGGACCCGAGAGAGTCGACCATGAAGATTACTAACAATTTCATCAAAACCGAAACGGTCTCCTTAGATAGTTTGTTGGATCTTTCTTCTGGGTGAGTTGCTTGTCTCATTAATGGTGCAGTTTCATCGTTAACGTCCAATGGTTCAGCTGTTTCTTCATTGCAATCTGTATGTTCATAATGCCCGTCCAATTCTGTAGCATCTGATAATAAGAGCATGATGACCATTTTGCAAAAGGCGAAGAAGGCATACAGTAAGAAAACTAATTTATAACATTGCAAATCAGTAGCAGCTAAgCCAGTTCTTTTCAAAAGATCTACAAATATACCACAAAcTATTGCACCTAGAGCGCTTCCAATTGTTCCAACCAAGGCGTGAATAGCATAAATCTCTGGTCTTGCATTATGAGGACTCAGATGTGCAATCATGGCCTCTTCTATAGATTTGAAAGGCCCGACTTCATCACTTGAAGGCGATATAACACCGAAGATAGCAAATACTAGCAAGAGGGTGAAATTTTCGCTAAAACTGAAAACCAACCCACTTAACAACATCATTGCACAACCATAAACTAGGACTCTTCTTCGGCCCCAGGAATCCGCATACCAAGTAAGAATGTAAGAGCAAATTACATCCCCTGCCAGTGTTAATGACATAAACAGCCCAATTTTATCCTCTGTCATATTGATGGCATTTAAAAAAAGCGTCAAAACTTGATTTGTTAAACCATATGATAGAAGTCTAAGAAAGACGGACGCCCATAGTAGCTTTATGTCTCTACTTGCACACTTGAATTTAACAAAAATTTCCGGTGCCATGGTTACGTTAACTTTAGAAATATTGGTAAATCCTTCTGCAATGAGCTTTTTCGA

*ENT3* MAR 1and *kanMX* amplification primer 2:

TTTCGTGAAATAGTTTGTGAATCAAGACAAACATATAAAGGAGAAATACCTTGACAGTCTTGACGTGCGCAGCTCAGGGGCATGATGTGACTGTCGCCCGTACATTTAGCCCATACATCCCCATGTATAATCATTTGCATCCATACATTTTGATGGCCGCACGGCGCGAAGCAAAAATTACGGCTCCTCGCTGCAGACCTGCGAGCAGGGAAACGCTCCCCTCACAGACGCGTTGAATTGTCCCCACGCCGCGCCCCTGTAGAGAAATATAAAAGGTTAGGATTTGCCACTGAGGTTCTTCTTTCATATACTTCCTTTTAAAATCTTGCTAGGATACAGTTCTCACATCACATCCGAACATAAACAACCATGGGTAAGGAAAAGACTCACGTTTCGAGGCCGCGATTAAATTCCAACATGGATGCTGATTTATATGGGTATAAATGGGCTCGCGATAATGTCGGGCAATCAGGTGCGACAATCTATCGATTGTATGGGAAGCCCGATGCGCCAGAGTTGTTTCTGAAACATGGCAAAGGTAGCGTTGCCAATGATGTTACAGATGAGATGGTCAGACTAAACTGGCTGACGGAATTTATGCCTCTTCCGACCATCAAGCATTTTATCCGTACTCCTGATGATGCATGGTTACTCACCACTGCGATCCCCGGCAAAACAGCATTCCAGGTATTAGAAGAATATCCTGATTCAGGTGAAAATATTGTTGATGCGCTGGCAGTGTTCCTGCGCCGGTTGCATTCGATTCCTGTTTGTAATTGTCCTTTTAACAGCGATCGCGTATTTCGTCTCGCTCAGGCGCAATCACGAATGAATAACGGTTTGGTTGATGCGAGTGATTTTGATGACGAGCGTAATGGCTGGCCTGTTGAACAAGTCTGGAAAGAAATGCATAAGCTTTTGCCATTCTCACCGGATTCAGTCGTCACTCATGGTGATTTCTCACTTGATAACCTTATTTTTGACGAGGGGAAATTAATAGGTTGTATTGATGTTGGACGAGTCGGAATCGCAGACCGATACCAGGATCTTGCCATCCTATGGAACTGCCTCGGTGAGTTTTCTCCTTCATTACAGAAACGGCTTTTTCAAAAATATGGTATTGATAATCCTGATATGAATAAATTGCAGTTTCATTTGATGCTCGATGAGTTTTTCTAATCAGTACTGACAATAAAAAGATTCTTGTTTTCAAGAACTTGTCATTTGTATAGTTTTTTTATATTGTAGTTGTTCTATTTTAATCAAATGTTAGCGTGATTTATATTTTTTTTCGCCTCGACATCATCTGCCCAGATGCGAAGTTAAGTGCG

*ENT3* MAR 3 and *ENT3* MAR 4:

AGCGTGATTTATATTTTTTTTCGCCTCGACATCATCTGCCCAGATGCGAAGTTAAGTGCGCAGTGGAGATTAGATAGTAATGTTCGTAATACCTGTTACAAAGTACTTTCTATTGTTATAAAACATTTACATATTGTGTAAACCAATAATATCACTCAAAAGGAAAGTAAATCGATTTCTTTGGATGATGTATGGTTATTATTGGTATTCTGTTCTTGTTGAACAGCTCCACCATGCATTTCACCAAATTCGTCATCATCATCTTGATCTGTACTTAGTGCAGTTGTGCTATTAGATACAGGATTAGAGGCAGCGGCTTTCGCTTGGGATGCTTTAGGAGCACTAGGTGCTTCTGCAGATGCCTTTGCAGTTGAAAAGAGAGAGCTAAAAGGATCTACTTTTTTAGGTTCCGCAATAGCTGGGGTAATCTTGCCTTGGTCGGTAGTGGAGCTGTTATAAAAAGGCATTGAACTCGTGGTGGCGGGCATACCCTCAATTGGGCTCGTGTTTAGCAAGTTAAACGAATTAGCAGGGTTGGTAACAGGGACGGCGCTTTGAAATTCTGAAAACTCGTCATCATCGTCCTCTTCTTCGTCCGCCTTCTTTTCATCTTCTTGTATGAGTTCTTTAGATGGTTTTGAAGACTCACTAGAAAAAAAGTCAACAAAGTCCTCTGGTTCTTGTTTTCCTTGCCCAGGAGTAGATGTGGCTCTCGAGGCATTATCGTTATATCCATTTTGACTAAAAGAACCATCTTCGTTATCTTCATTGTCACTATCGAAATCAGCACTCACGCTAATGCCGTGCACTTTCGTTGACGTGAAACCTGCCTTCGAATTCAAACTGCCATCAGCAGAGGCAGATCCTCCAGCAACGCCCTTGTACTTCTTTGCtGTTTCTCTTGCCTTTTTTCTTTCTGCGCGTATCTTATTATCGTCGCTCAATAATTCAATCAAAGCTTTGACCCTAGTTCTGACATTAATTCCTTGATCTCTTCCCTGGGAATCAATATAGTGAAAAGTTTCTAAAATCCTAATTAAGTTAATGCTATTCCTTGTATCATCGATAAACCTTTCAGAACCGTGTTTGATAAGGTAATCGAGCAATTGCAAGGCTTTATAAATTTGCCTCCACTCACTACCTGCCTTTTCAGTGAACCTTCTGAATATCATGGACAAAATTTCTTCTCTTTCCCTGAAATTGTAAGTTCCTTGAGAAATCTGGTCCATTAAAGTGGATGAGGCACCCCAAGGCTCGTTGTTTGTCGCTTCACGAACTTTCCCCTCCATCTCAGTATAATTGAAAACAACATTTTGAGCTTTACGAAAATATTTCTTGGCGTCATATAATGACATGTTGGCTAATGTATCCTCTAAACTCATTCTTCCTAACACTAATTAACGATGTGATCTCCTATCGACGTCCC

*VPS70* MAR 1and *kanMX* amplification primer 2:

GTATAAGAAATTGAAGCCATGTTTATAGAGTATATACAAAAAAGCCTCTCATGGTTATTACCTTGACAGTCTTGACGTGCGCAGCTCAGGGGCATGATGTGACTGTCGCCCGTACATTTAGCCCATACATCCCCATGTATAATCATTTGCATCCATACATTTTGATGGCCGCACGGCGCGAAGCAAAAATTACGGCTCCTCGCTGCAGACCTGCGAGCAGGGAAACGCTCCCCTCACAGACGCGTTGAATTGTCCCCACGCCGCGCCCCTGTAGAGAAATATAAAAGGTTAGGATTTGCCACTGAGGTTCTTCTTTCATATACTTCCTTTTAAAATCTTGCTAGGATACAGTTCTCACATCACATCCGAACATAAACAACCATGGGTAAGGAAAAGACTCACGTTTCGAGGCCGCGATTAAATTCCAACATGGATGCTGATTTATATGGGTATAAATGGGCTCGCGATAATGTCGGGCAATCAGGTGCGACAATCTATCGATTGTATGGGAAGCCCGATGCGCCAGAGTTGTTTCTGAAACATGGCAAAGGTAGCGTTGCCAATGATGTTACAGATGAGATGGTCAGACTAAACTGGCTGACGGAATTTATGCCTCTTCCGACCATCAAGCATTTTATCCGTACTCCTGATGATGCATGGTTACTCACCACTGCGATCCCCGGCAAAACAGCATTCCAGGTATTAGAAGAATATCCTGATTCAGGTGAAAATATTGTTGATGCGCTGGCAGTGTTCCTGCGCCGGTTGCATTCGATTCCTGTTTGTAATTGTCCTTTTAACAGCGATCGCGTATTTCGTCTCGCTCAGGCGCAATCACGAATGAATAACGGTTTGGTTGATGCGAGTGATTTTGATGACGAGCGTAATGGCTGGCCTGTTGAACAAGTCTGGAAAGAAATGCATAAGCTTTTGCCATTCTCACCGGATTCAGTCGTCACTCATGGTGATTTCTCACTTGATAACCTTATTTTTGACGAGGGGAAATTAATAGGTTGTATTGATGTTGGACGAGTCGGAATCGCAGACCGATACCAGGATCTTGCCATCCTATGGAACTGCCTCGGTGAGTTTTCTCCTTCATTACAGAAACGGCTTTTTCAAAAATATGGTATTGATAATCCTGATATGAATAAATTGCAGTTTCATTTGATGCTCGATGAGTTTTTCTAATCAGTACTGACAATAAAAAGATTCTTGTTTTCAAGAACTTGTCATTTGTATAGTTTTTTTATATTGTAGTTGTTCTATTTTAATCAAATGTTAGCGTGATTTATATTTTTTTTCGCCTCGACATCATCTGCCCAGATGCGAAGTTAAGTGCG

*VPS70* MAR 3 and *VPS70* MAR 4:

AGCGTGATTTATATTTTTTTTCGCCTCGACATCATCTGCCCAGATGCGAAGTTAAGTGCGTTCAAAGTCACTCTACGTATGCAAATAAAATAAATGTATATACTGAGGTGAGGTACTTAAATGCTTATTGAAGTAAATAGCGAACGTTGCTGAATTGGCTTAGCAAAATGGTTAACCACTGAATGACCTCGTTTCTATCAATATCAGCCAATCCTTCGTGAAGGCCGGGCAAAACTTCCCCGACAGATCCCTCAAACTTACTAGGTGCTAAAAGAGAGTACTTCATCCATTCCCTGTCTTTCAGTCCTCTGTGTGTAATAAATAATTGATCAATTTGCTTTGATTTGCTATTGGATCGTTGCAGTTTAATATAGCTCTTGATTTTTTTGTAAAAAGCCCAAACAGGTAAAGCTTCCCTACACTCTTTATAGAGAATAtCATTTTGTTGGTCAAATTGGATGCTATCTTCCTGTGTGGCAACTTTTAATAAGTCCAGAACCCTTTTTGCTAAGCTGTTCACTTCATCGTCCTGGGGAAATGCTGAAGATAGATTAGAGTGCCATGCGATATACCAGTTATATATTTTCTTCAGGTAAACATGTGTATTGAATCTTGCCAGTTCGTTTTCACTCAGCATCAGCGTTGTCAAACCTACAAACATGGCCATGGTGTTGTGTAACTTGTAGTCAGAATTGGTAAATTTTTCCAACCAAGTTGGGCTATCGAATACGGAGTTACTATGATAGACTGCGCCTGAAGTATCATTGGCATTAAACTGAAAATGTGCAGCGGGCACTCCAAGATGGTACTGAAATGAAGTGTAACTAGACAACCCATCAAGTAGAGAAATAGTGGCATTAGAAGTGTATTTCCAATGGTCGAACAATGACCAGTCTTCGTGCCCATTAAATTCCGTGAGCTTAGCAGCTTCGTATATCACGTCTTGTAAAAGTGGGTTGGCTTTACAGTGAAAATTTGTCCCAGAGATTGCATTATCTAGATTTAGGTATACCAAGGCCCTTCTCCTGAGAATCGCAGCATGAGCTTCTGCATAATCTGTAGATCCCAGAAGGCCGGATCGTTCACCATCCCAACTTATTAGTTTGATAGGACGCAGTGGCTTCCAACCATGCTTAAGTAATTTACTCATTCCTCGTGCAATTTCTAAAAGAATAGCGCTGCCACTATTTGCATCACCGGCGCTACTCGAGGCGAGCGAATCCCTATGAGCTCCGATAATAATCTCCCCCTCAGTGAATATACCAGGGATACTAACCTCTACACTACTCATTTCCTTGATGTTGTAAGTTAGCTCATTATGCAAATGGACTTTATCGATAGAGCTTGAAGGTCCAGTGAATGATCCAAAATCTTTTATATTACTACCGGGCCCAATTTGAAAACCCCTGCCATTCAATCTTTCTAAAATTGGTTGGACATCTCTAGCACTCATCGGCACCGATGGTATCCTCGGCACTCTCCCTACCGGTGACATATGTTCAGTGTCGGAATCCTTGGAGGGATACCCTGGAGTTGTCGGATCTCCTGGAGTGTCACTGAAATAGTTTACAGAATCTCTCtTAATATAACTTGGGTTTCTTGCTGGTCCATAAGGATAGTGTAAAAACCCATTTTCCTCAGTAACTTTACCATCGTCAAATGGGTCTGTATATATAATGACACTGGAAGCGCCATAAAGTTCTGCATTCTTTACCTTTAATCCAGGTAgTATTTTACCCGATCGTACGATGTGGATTTTATCTTCAATATCAATGTTTTTCTTCAAAAGTAGCTTGTAgTCACTGATGCTACCATAATTGCAAAACACGTATCGAGCAGTTACACTTCCATTTTTTGAATATTGATGGAAACCCTTTTGCCTTTTCCTAGCGTGAGAAGCAGGATCACCTTTAACTCTATCCTCGATCATGCTTGCTTCGTAGACCACTTTACCATTTTCTAAAaGAGCTACGTTAGTGTCTACTGGTTCACCTATCCATGGGTAATATTTTTCAACTTTGGGTTTGTACCCCATATCTAGAAACTCGTCCAAGGTATATTTAAACGTTGACGAATCACTTGCTCCGTTGCTCATGTACCCAGCATACTTGTATACATGTTCTTTCGCTCTATTTTCCTGTTGCAAGGAGTTCAAGTATATCCTATAAACCTCTGCATTCGTCAATCTGGAAGAGTGAAACCGCCGAAAGTCTCTTGATAAAGACGTCCTGGGAGCAAATGCGGCAACAAATCCCATGTACAAAAGGAGACTCGCTAGAATCAAGTACATAAACTTTTCCTTGTCCATATGTTCTTTCATTATTTCATAACCACTAACAATACTAGATATTGTATTACTTCTGCCTCTATTCCTGGATGTTAAAGTGAAAGAATCATTCATGCTTTCTTCATTGTCATCGCGAGTGGTCTCaCCACTATATTGTTGTAATCCAGTGGCTTCGGCCTCATTGTCATCAGGGTCTGCCATATTAACAACAGTTCTCTCCTTTAATTGACCTTCCTCTTTCTCTCTCTTTCTCTCTCTCTGTATCATTCTCATATATCTCTTACATTCTTAATTGTGCATGTTGAAATTGTTTTAATAAGTACGGCCCCTTGC

*RSF2* MAR 1and *kanMX* amplification primer 2:

TTAATAAGTACGGCCCCTTGCCTTGTCAAACCTTTTTACCCGGGCCATCAAACTCAAGCACCTTGACAGTCTTGACGTGCGCAGCTCAGGGGCATGATGTGACTGTCGCCCGTACATTTAGCCCATACATCCCCATGTATAATCATTTGCATCCATACATTTTGATGGCCGCACGGCGCGAAGCAAAAATTACGGCTCCTCGCTGCAGACCTGCGAGCAGGGAAACGCTCCCCTCACAGACGCGTTGAATTGTCCCCACGCCGCGCCCCTGTAGAGAAATATAAAAGGTTAGGATTTGCCACTGAGGTTCTTCTTTCATATACTTCCTTTTAAAATCTTGCTAGGATACAGTTCTCACATCACATCCGAACATAAACAACCATGGGTAAGGAAAAGACTCACGTTTCGAGGCCGCGATTAAATTCCAACATGGATGCTGATTTATATGGGTATAAATGGGCTCGCGATAATGTCGGGCAATCAGGTGCGACAATCTATCGATTGTATGGGAAGCCCGATGCGCCAGAGTTGTTTCTGAAACATGGCAAAGGTAGCGTTGCCAATGATGTTACAGATGAGATGGTCAGACTAAACTGGCTGACGGAATTTATGCCTCTTCCGACCATCAAGCATTTTATCCGTACTCCTGATGATGCATGGTTACTCACCACTGCGATCCCCGGCAAAACAGCATTCCAGGTATTAGAAGAATATCCTGATTCAGGTGAAAATATTGTTGATGCGCTGGCAGTGTTCCTGCGCCGGTTGCATTCGATTCCTGTTTGTAATTGTCCTTTTAACAGCGATCGCGTATTTCGTCTCGCTCAGGCGCAATCACGAATGAATAACGGTTTGGTTGATGCGAGTGATTTTGATGACGAGCGTAATGGCTGGCCTGTTGAACAAGTCTGGAAAGAAATGCATAAGCTTTTGCCATTCTCACCGGATTCAGTCGTCACTCATGGTGATTTCTCACTTGATAACCTTATTTTTGACGAGGGGAAATTAATAGGTTGTATTGATGTTGGACGAGTCGGAATCGCAGACCGATACCAGGATCTTGCCATCCTATGGAACTGCCTCGGTGAGTTTTCTCCTTCATTACAGAAACGGCTTTTTCAAAAATATGGTATTGATAATCCTGATATGAATAAATTGCAGTTTCATTTGATGCTCGATGAGTTTTTCTAATCAGTACTGACAATAAAAAGATTCTTGTTTTCAAGAACTTGTCATTTGTATAGTTTTTTTATATTGTAGTTGTTCTATTTTAATCAAATGTTAGCGTGATTTATATTTTTTTTCGCCTCGACATCATCTGCCCAGATGCGAAGTTAAGTGCG

*RSF2* MAR 3 and *RSF2*MAR 4:

AGCGTGATTTATATTTTTTTTCGCCTCGACATCATCTGCCCAGATGCGAAGTTAAGTGCGAACTCCGAATGGAACAATGCCTTTTTGAAGCCTGGTGGTATATTATGAAAGTTATGATAGTTCTAAGCTTTAATTCTGTAAATACTATAGTATAGAGACGGCCGCCATTATATATTTGTAAATtGGACTTATATTCTAGTGTTTCTTTTTTTCGTAACACTATAGATAGCTCTTGATTGCAAACCATGAGCGAACGATAATATTATAGGCCAAATTGGCGCATCACCtAATATTTGAACACCCAAATATAAACATTTGGAGGATAGACTTGCCGCTACAATTAGTTGAATTGTCTCATTTATATCGGTATTCGGGTCCATGGCCCTTTGTGCTTGTTCTTCATTTAGTGCTGACAAATCTAGGGCATCCTTGTCCTGCCATCTTAAGAAATCAGTGTACGATTTCAACACATCACACTCCTTCGGTATCAAGTTCATTTGTAATCTTCTCAAAATCCTTTCTGCTTTTAGCCATAAGACACGGTCCGAAAAATCCAATAATGCTGAGTTAGGGTTGTTGGCATTGTACCCGCGTGCCCAATCCTCTACACATTTCATGTATTCCGAAATCACTAATACTGCAGTGAACATACATGTGGTCGCAAATACAGGAGTATTGAAAATTCTGCGCTTGCCCcGTTTAATGTACGTAAAAAATGAAGTCCACGCATCCACCATATTCAGTGCATACTCGGTAGCTTCCCTCAATGAACCCATGTCATAGCAAACTTCCTCCAAAGCTTTATTCATATTGGACCAATCTTTCAACCAGATTTTCTCAATAACATGTGCCAGGTCCAAACACCGTCTCATTTTGGCAAACAAATATACCGGAATTATTAACCTCATTGCTGGATTGGCGTTTATTGTTGACATGCTATTCTCGGTAGGTGTCAAGATGCCACCATTTTTCAAATAAAGGTTTTCCCAGTATTTTAACATTGTATCGATCCGTTGTCTGGAAGTCATCTTCCACTCAATATCGTCtAACTCAATGTTATTGCTATTGGTGTTGTTATTACTGATCCTTGCGTTATTTCTTTCAATAAGTATTTTCTCATGGATAGATATCAACAATGATAGACATGTACTTAACGAAACCCTAGCATTcCCGTAAAAAAAACTATCGCCTGTAGAAAGAAAtCTCAAACAATTTTCATATGCCTCGTTACCATTAGACAATTCAATCAACGAAAATTTCGAATCTAACGTTATTTTGTATTGACATAATAGGTCCGACCATTCATCGGAATTTCGGCACTGATATAATTCTTCTTTATAGCATGGAACACCACATTTTAAATCGACTGAATGAAAACAGCAATCAGCACCTACCAGTGAAGAAAATAAGTTAGATATAAGTAATACCGCATGGCAAGTCCTGATACGTGACTGCGCCAATACAAAATATTGGTAGTTTTTGTTCATTTGATTCGGCGCGTTGTATTGCTCTCTTATTTTTGAAAACATATGAGGACTGTTTTCATATTCCATAATATGATCACTCTCAATAGGCGGCTGGCATAGGGACTCTAGGGGCAAATTTAAACGAGAGGCCTTCAAGAGACGAATAATAGTCGTCAACTGACCATGCATCCCCTTGATCACGGCTGTgTTTTTATTGAAAATACAGTAGAACGTTAGCAATACTAATGTCTGTATAACCCATAACTCTGTCGTCTCCGGGTTTTTCTCACTAgCTTTCAAGCTTTTCCTAATTTGGGTGCTAGCTGCATTAGCTAACACTTTTGCATGCGTtGAATGAAACCCATACAATGCTCCGACCATGGAGATAGATAATAACAAGGGATAACTGTCCATATTAGGTATGATAGAAGGAAGATGAATAAATGAAAAAAATGGATGGAATTCTTCCTTATAGTAAGTCACATAATCGTTCAATTGACTAGTTGTGGGGAAGGCTCCTGAATCGATATTATTGTCGGCTAGCATCATACTCCTTAGtTCATTGGTAAAAAGCAgTTGAGATACTGAAGGaACAGAAAACGGAGATAACAAATCCGAAGGAGTAAGAACAGGCAGCTGTTTCTGTGTTGCATTAGAATCAGTTTCGCCGGGGAAAGACAAAGTGAGAACATCGTCGTTCACATGAGAATTATTTTGCTCTGTCCCGTTTAATACCTTGGAAATATCCAATTGCCTTGAGCTGAAAAAATTGGATAGATCGTAGTCCATCATGACGCTATCACGACGCCTCTTATGAGAACTATGGGAAATAGGGTCAGAATAGGAAGGCTCtTTcAGTTGGTTTTGTGGTGTACTTGGTAAATCTGTGCTTCCATTCAAACTCAAGTTTAAGCGAGGTGATAAAGAAGCTTTTTCATGATTTAATGAGGTAGCAGAATGGTTTGGAGGTGACTTGcTTGGTATAGATGACCTTGATGAAGGTGGAGAATAAATAAACCCAATGTCATTGAAATGgTTGAAGTTGATCTTGAAGGTAGATTTTGGATCAGAaTTGTTAATAAACTCTGATAACCACTTGTCATCTATGGCAGCAGGATCGATATTACTATCCTTATCATTGGGCTCCATTGATGGTTCCAGTAAATTATTTGTGCtTGGATTGAAATTGATCGTGGAAGAGGAgTCTGAAACAGTTGTTGGTGGGTTTTTAGGAGATAGTGACATAAGCGGCTGCTCATTGCTCATTTGCCTATAAGTTTGATTTGCCAAATTACTGTAACTATTAGAAGCtGTAGTAGAATTAGACGGTATAGTATTCGGGAAGCTTGACGGAAGGTTAAATTCAGAAACTGTCGGTTTATCATGACTGAAGCAGCCCAAATCAAATGGCGAATGGTGGCTAGACATGTATCCTCCCGCACCTGCATGTGCAGAGTTCATTGTCAAAAGATCGGTTAAAGATGGACCGCTACTTAAGAGATACACTCCAGGTGTAGCAACTtTACTTCCATTGTTGCTGTTCACTATGGTACTAGAGGTGGAATTTTGCTGTTGTTGTTGTTGTTGTTGTTGtTGTCCGGAGGGGTCATTGTTAAATAGATTTAAGTTAAACTTGATAGCTTGCGGTGGTTCGTCCAGGGTTAACGGAGGTAAATCCAATTCTGACACGCTTTCCATGAGCTGCTTCGCGGTCATTTGTGGAGTgGAAAAaCCGACTTGATGCGGAGCGTCTTCAACGAGCTCGAAATTGGTAATTGAATGGTGGGGGCTATTGCTACTAGACATGTAAGTCATTGCACTGGATGCAGAGAAAGACGCATGTCTTTTCGTCTTATGCATGGAAGAGGGAGTGTTCaCCCTGTCGATAACAGCATCGCTTGATGCAGAGTGAACTGGCACGCCTTTCGTGCTGAACTTGAACTCTCGAAATtCTAGGGAGCCTGCCTTACTAGGAGGAGTTTTCTCTCGCTTATCGcTaATTACTGGGGaCGAGACGGGAAGTTCTACATTATTTGATTTGGCCAAGGCAACCACGGCCTCTTTCAAcTCTTCAGATGTCTTACCAGtAAGGTTCTTCGGGGTGGGTAGAATAGTCTCTTTATTTCCaGCTATTTTAATGATATGAAGGTCGGTTGGATCATCCGCCGCCACGGAATGGCGCCGCTTGGAGGCAAAAGAAGAAGTCGAATTTGGTGCTGGCGTgATTCGCCGTGGATCCCCCGTACCTACAAGAGCAGCATGAAGTTTTTGCTGATGCCTGAGCACTAAATCTCTACGAGCAAAACACCTACCGCAAAAGATACAAAGATACGGTTTCTCACGGGTATGCGAATGCTGATGTCTCTTCAAATGCTCCTGCCTGACAAAACCCCTAGTGCATGTGGGACACAAGAACGGTCTTGGCTTATCAGTCTTAATAATTCGAGATTTTTTAGGAATGGGCAGAATACCTTGACCAGCGGGtTTAGCGAAATTAGATTGATTACCGTTCACAAACATACTTGGCTCCGGCACTAATAAAAAATCTTTATCTGCCCTATCTATTCTAGTATCaCCAATGTCTTTTAGCTTCTTCGCCCTAAAGTTTCTTTTTGGTGCAAGAGTCTTCGAGAAAAACGTGTGTTGAGAGAGGCAATGTTTAACGAAGAAAAAAAACTCTATGGAAATCCAAGAAGTCCTTGTTTCTTTGTTTTCGTTTCTAATATATGCAGGGTCAAGCGGGAAAAAGAAAGACAGACGGAAAAGTGCCCAGCAACACGGAACAAAATTGTCCAGATTTATTCGAGCGGCCGCGGTTcGTATGCATAATGCAGGCGCCCCTCGTCCAAATGCGAACGGTTCCATCCGAAACAGGCCATTTCCCAAAggTTATTTCTCTTAATgGAAGTTGCTCGCGTAGTTACCTATTGTCGATGCCGCATACGCAGTTACCTTATCCGTTGCCCTACGGAGAATTTCCGCGATGCAGCACCGTGCGGCGAGATCACAATTTTCCTTGTACACTCTACTCTGCCCCTGGAGTGGTTCACCCCCGTCTGTGCGTATTTTCGTGTATGATGATATAAGGTCGTTACTACCGTTGATTCAAAAACAGTTGCTTCTTCCATATATTGTAGCACGAACAACTATGGCCGGCCGCAAATTGCGACAATTACACCTAACGCAGCATTAAGGTCGCAGGAAATGTTATCTGCCGAGCTGTTTATCTCTGTTTTCCAAGATTTTTTCAACCAGCCCATGAGTCTGCTGTTGGCGGCAACCCAGTTACAGATAATGGTGCATGTGATAAGCTTCGcACCACTATTCGTAGTTTGTTACATCTTGTAAGCCAGTATATATATTTGTTAGATTGTGCCCTAACGTTCCAGTAATTGcAGGGTAACCGAGGTTTCCGTGGAAAAAAACCGTGTATAAGCAAGAGGTTCCGAGCGAAAAAAAAAAGTATAAAAGAAAGCACAGGCAATGAGATTTGTTTTTCGCTTTTCCTTCTTATTTGTTAATTCTATTTTTGAAGTTTCCTTCCGAAAAAAAGTAACTTTGTCCTAAAGTACTAATCCACCGCATTAGACAGTACG
